# Chiral phosphoric acid-catalyzed transfer hydrogenation of 3,3-difluoro-3*H*-indoles

**DOI:** 10.3762/bjoc.20.20

**Published:** 2024-02-01

**Authors:** Yumei Wang, Guangzhu Wang, Yanping Zhu, Kaiwu Dong

**Affiliations:** 1 School of Pharmacy, Key Laboratory of Molecular Pharmacology and Drug Evaluation, Ministry of Education, Collaborative Innovation Center of Advanced Drug Delivery System and Biotech Drugs in Universities of Shandong, Yantai University, Shandong, Yantai, 264005, P. R. China; 2 Chang-Kung Chuang Institute & Shanghai Key Laboratory of Green Chemistry and Chemical Processes, School of Chemistry and Molecular Engineering, East China Normal University, Shanghai 200062, P. R. China

**Keywords:** asymmetric organocatalysis, chiral Brønsted acid, 3,3-difluoroindoline, Hantzsch ester, transfer hydrogenation

## Abstract

A convenient and efficient method for the synthesis of optically active difluoro-substituted indoline derivatives starting from the corresponding 3*H*-indoles by chiral phosphoric acid-catalyzed transfer hydrogenation was developed. Using Hantzsch ester as the hydrogen source under mild reaction conditions, the target products can be obtained with excellent yield and enantioselectivity.

## Introduction

**Figure 1:**
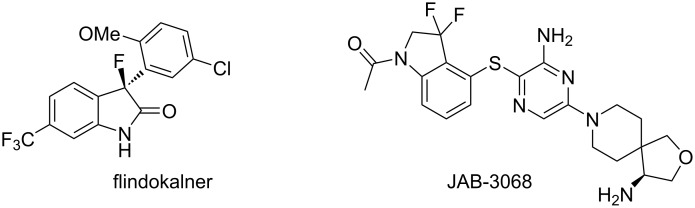
Structures of bioactive fluorinated indole derivatives.

The introduction of fluoro atoms into organic molecules can alter their lipophilicity, solubility, metabolic stability, and increase drug activity by affecting drug receptor interactions [1]. Therefore, replacing hydrogen with one or more fluoro atoms has beneficial effects on therapeutic efficacy and pharmacological activity [2]. For example, flindokalner is a potassium channel opener (Figure 1) [3]. JAB-3068 is a promising SHP2 inhibitor that has entered phase II clinical trials for the treatment of solid tumors and has been approved by the FDA as a rare drug for treating esophageal cancer (Figure 1) [4]. Among the fluoroalkyl moieties, the geminal difluoromethylene group has showcased its beneficial properties as an isostere of polar functional groups [5–6].

**Scheme 1:**
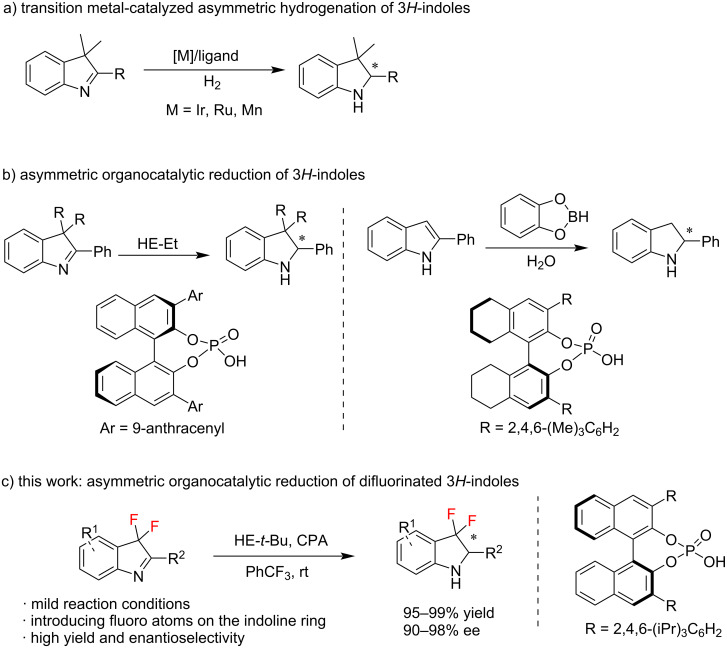
Synthesis of chiral indolines via asymmetric reduction.

Chiral indoline is an important member of the class of nitrogen-containing heterocyclic compounds that often exhibits various pharmaceutical activities and exists in many natural products [7–8]. The enantioselective synthesis of chiral indolines has received great attention in organic synthesis. Various methods [9], including reductive hydrogenation [10–11], kinetic resolution [12–14], functionalization of indole [15], and de novo construction of chiral 2-substituted indolines, have been developed [16–20]. In recent years, the metal-catalyzed asymmetric hydrogenation of indoles to synthesize chiral indolines has been widely studied (Scheme 1a) [21–22]. Representative examples include Ir- or Ru-catalyzed asymmetric hydrogenation of 2,3,3-trisubstituted 3*H*-indole [23–24]. Generally, these methods employ precious metals and/or relatively strict reaction conditions (up to 150 bar H_2_). In 2022, Liu’s group reported an asymmetric hydrogenation of 3*H*-indoles catalyzed by a chiral Mn complex, which showed good yield and enantioselectivity [25]. In addition to metal catalysis for the enantioselective reduction, asymmetric organocatalysis using chiral phosphoric acids has also been studied (Scheme 1b) [26–28]. In 2010, Magnus Rueping and his co-workers developped an enantioselective Brønsted acid-catalyzed transfer hydrogenation of 3*H*-indoles [29]. In 2020, Song and Yu successfully applied a new chiral Brønsted acid, synthesized in situ from a chiral boron phosphate complex with water, for asymmetric indole reduction (Scheme 1b) [30]. The mild reaction conditions, low catalyst loading, and high enantioselectivity rendered this transformation an attractive approach to synthesize optically active indolines. However, these asymmetric reduction studies focused on alkyl or aryl-substituted 3*H*-indoles whereas the synthesis of chiral difluorinated indole derivatives could have potential applications in pharmaceutical chemistry. Herein, an organocatalyzed transfer hydrogenation of 3,3-difluoro-3*H*-indoles to obtain fluorinated 3*H*-indolines was developed (Scheme 1c). With this method, a variety of chiral 3,3-difluoroindolines were synthesized in high yield and enantioselectivity under mild reaction conditions.

## Results and Discussion

We conducted a preliminary exploration of the reaction using 3,3-difluoro-2-(phenylethynyl)-3*H*-indole (**1a**) as the model substrate, Hantzsch ester (HE-Et) as the hydrogen source, and BINOL-derived chiral phosphoric acids (**CPA**) as the catalyst (Table 1). With chiral phosphoric acid **CPA-1**, the transfer hydrogenation reaction proceeded well in PhCF_3_ at room temperature and the target product **2a** was obtained in 98% yield with 20% ee after 12 h (Table 1, entry 1). Then, the effect of steric hindrance of the **CPA** catalyst and solvents on the stereochemistry of this transfer hydrogenation were investigated in detail. Among various 3,3’-disubstituted **CPA** catalysts (Table 1, entries 2–6), chiral phosphoric acid **CPA-6** containing 2,4,6-triisopropylphenyl-substituents at the 3,3’-positions of the binaphthyl skeleton performed best giving the target product in 99% yield with 91% ee (Table 1, entry 6). This suggested, that the steric hindrance of the **CPA** catalyst at the 3,3’-position is important for achieving high selectivity. Also, an obvious solvent effect on the enantioselectivity was observed (Table 1, entries 7–10). Very low ee values of product **2a** were detected when the reaction was performed in DMSO or MeOH (Table 1, entries 7 and 8), while using DCE or toluene as the solvent the enantioselectivity dropped significantly (Table 1, entries 9 and 10).

**Table 1:** Reaction optimization studies.^a^

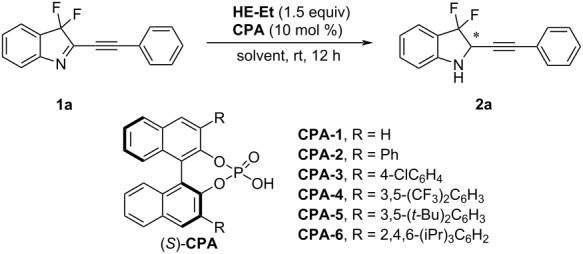				

entry	chiral phosphoric acid	solvent	yield (%)	ee (%)

1	**CPA-1**	PhCF_3_	98	20
2	**CPA-2**	PhCF_3_	99	24
3	**CPA-3**	PhCF_3_	99	67
4	**CPA-4**	PhCF_3_	99	40
5	**CPA-5**	PhCF_3_	99	76
6	**CPA-6**	PhCF_3_	99	91
7	**CPA-6**	DMSO	99	7
8	**CPA-6**	MeOH	99	7
9	**CPA-6**	DCE	95	82
10	**CPA-6**	toluene	99	85

*^a^*Reaction conditions: **1a** (0.1 mmol, 1.0 equiv), Hantzsch diethyl ester (1.5 equiv), **CPA** (10 mol %), solvent (1 mL), rt, under N_2_ atmosphere, 12 h. The yield was determined by ^19^F NMR spectroscopy and the ee value was determined by chiral HPLC. DMSO: dimethyl suifoxide; DCE: 1,2-dichloroethane.

To further improve the enantioselectivity of this **CPA**-catalyzed transfer hydrogenation, we next explored the effect of the alcohol part of Hantzsch esters (Table 2). The experimental results showed that the ee value of product **2a** increased as the steric hinderance of Hantzsch ester raised (Table 2, entries 1–3) and switching from ethyl to *tert*-butyl esters the desired product was obtained in excellent yield and enantioselectivity (Table 2, entry 3). Subsequently, we investigated the effect of the amounts of **HE-*t*-Bu** and chiral phosphoric acid on the reaction outcome. When the amount of **HE-*t*-Bu** was decreased, the reaction yield dropped (Table 2, entry 4). On the other hand, reducing the amount of chiral phosphoric acid to 1 mol % or the reaction time to 3 hours, still good reaction results were observed (Table 2, entries 5 and 6). However, the enantioselectivity decreased when the reaction temperature was reduced to 0 °C (Table 2, entry 7).

**Table 2:** The effect of Hantzsch esters and other reaction parameters.^a^

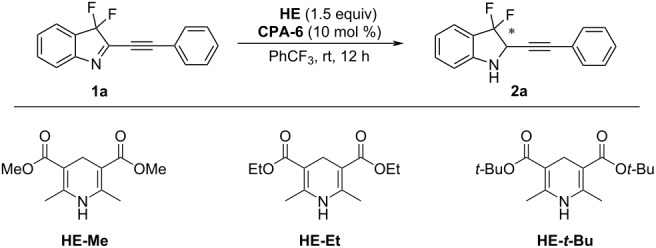			

entry	Hantzsch ester	yield (%)^b^	ee (%)

1	**HE-Me** (1.5 equiv)	99	83
2	**HE-Et** (1.5 equiv)	99	91
3	**HE-*t*-Bu** (1.5 equiv)	99	96
4	**HE-*t*-Bu** (1.0 equiv)	88	96
5^b^	**HE-*t*-Bu** (1.5 equiv)	99	96
6^b,c^	**HE-*t*-Bu** (1.5 equiv)	99	96
7^c,d^	**HE-*t*-Bu** (1.5 equiv)	99	85

^a^Reaction conditions: **1a** (0.1 mmol, 1.0 equiv), Hantzsch ester (1.5 equiv), **CPA-6** (1 mol %), PhCF_3_ (1 mL), rt, under N_2_ atmosphere,12 h. The yield was determined by ^19^F NMR spectroscopy and the ee values were determined by chiral HPLC. ^b^1 mol % of **CPA-6** was used. ^c^The reaction time was 3 h. ^d^The reaction temperature was 0 °C.

**Scheme 2:**
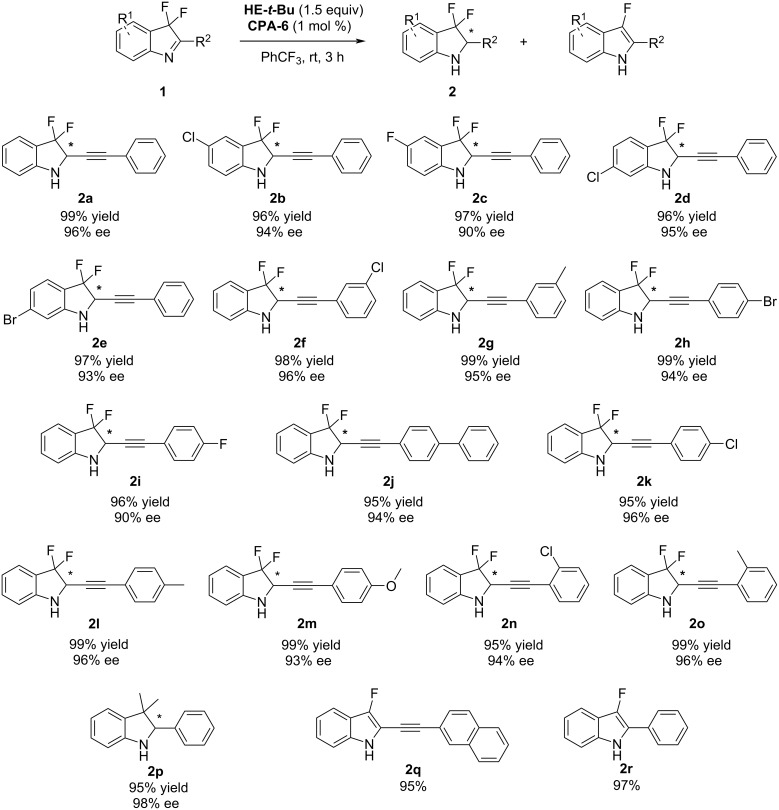
Substrate scope of 3,3-difluoro-3*H*-indoles.

With the optimal reaction conditions in hand, the substrate range of 2-alkynyl-3,3-difluoro-3*H*-indoles **1** for this transfer hydrogenation reaction was investigated (Scheme 2). Fluoro-, chloro-, and bromo-substituted 3,3-difluoro-2-(phenylethynyl)-3*H*-indoles were well tolerated, providing the chiral indolines **2b**–**e** in high yields and ee values. Various 2-alkynyl-3,3-difluoro-3*H*-indoles bearing electron-donating and electron-withdrawing groups at the *meta*- (**2f** and **2g**), *para*- (**2h** and **2m**) or *ortho*- (**2n** and **2o**) position of the aryl ring smoothly underwent this asymmetric reduction, affording the desired indolines in 95–99% yield and 90–96% ee within 3 hours. Replacing the 3,3-difluoro substituents by two methyl groups in the starting indole as well as the alkyne part by a phenyl group, the reaction still gave good results (**2p**). However, when using 3,3-difluoro-2-(naphthalen-2-ylethynyl)-3*H*-indole or 3,3-difluoro-2-phenyl-3*H*-indole as the substrate, the generated indoles underwent fast HF elimination/aromatization and finally gave indole derivatives (**2q** and **2r**) in almost quantitative yields.

**Scheme 3:**
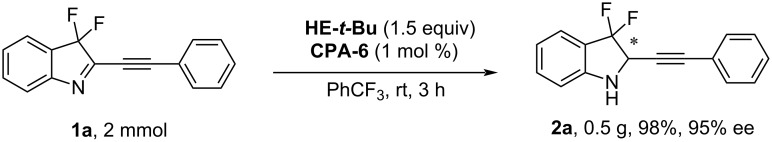
Experiment at 2 mmol scale.

To examine the efficiency and practicability of this approach, a 2 mmol scale experiment of the asymmetric transfer hydrogenation of **1a** was carried out (Scheme 3). Under the standard reaction conditions, 0.5 gram (98% yield) of chiral difluorinated indoline **2a** was obtained with 95% ee.

**Figure 2:**
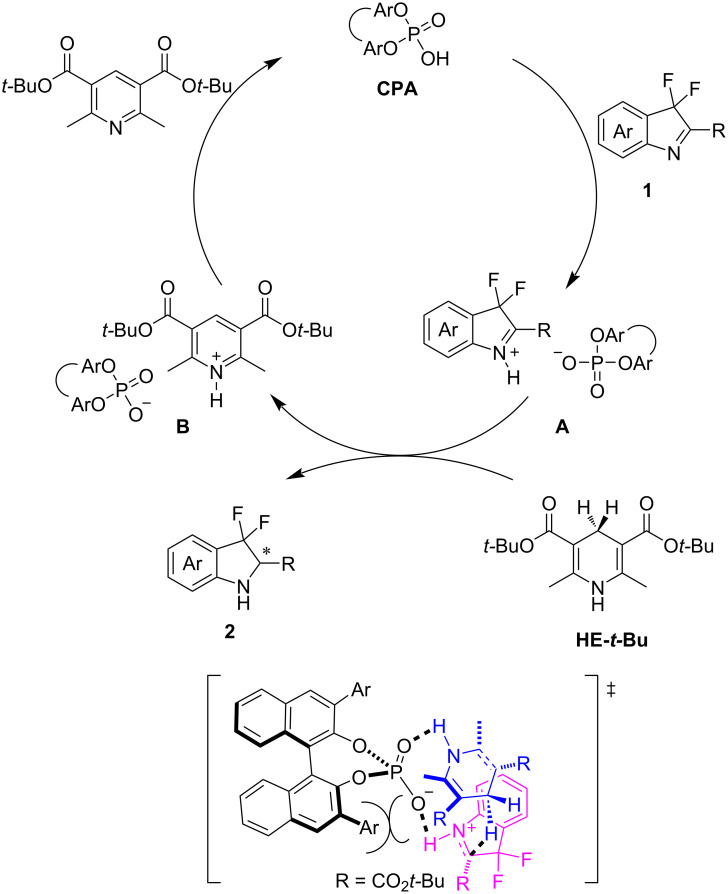
Proposed mechanism for the transfer hydrogenation reaction.

Based on previous studies [31], a mechanism of the **CPA**-catalyzed transfer hydrogenation reaction was proposed (Figure 2). The activation of 3,3-difluoro-substituted 3*H*-indole **1** by protonation through the Brønsted acid generates the iminium **A**. Subsequent hydrogen transfer from the Hantzsch ester gives the chiral amine **2** and pyridinium salt **B**. The **CPA** catalyst is regenerated from salt **B** through proton transfer. We deduced that the steric repulsion between the bulky 2,4,6-triisopropylphenyl-substitutents in the chiral phosphoric acid **CPA-6** and the carboxylic ester group of the Hantzsch ester hydrogen donor contribute to the high enantioselectivity of the reaction. The role of fluorine and alkyne in the reaction should be close to the *gem*-dimethyl moiety and the phenyl group in the previous research [32].

## Conclusion

In summary, we developed a convenient method for the synthesis of chiral difluoroindoline compounds for the first time. With a chiral phosphoric acid as a Brønsted acid catalyst and Hantzsch ester as the hydrogen source, a series of 3,3-difluoro-substituted 3*H*-indoles underwent asymmetric transfer hydrogenation under mild reaction conditions, giving the target products with excellent yields and optical purity.

## Experimental

General procedure: a 4 mL sample bottle was charged with 3,3-difluoro-substituted 3*H*-indole **1** (0.1 mmol, 1.0 equiv), Hantzsch ester (**HE-*t*-Bu**, 42.0 mg, 0.15 mmol, 1.5 equiv), and chiral phosphoric acid (**CPA-6**, 0.75 mg, 0.001 mmol, 1 mol %). Then, PhCF_3_ (1 mL) was added in a glove box under N_2_ atmosphere and the reaction mixture was stirred at room temperature for 3 h. After concentrating the mixture, the residue was purified by column chromatography on silica gel using a mixture of petroleum ether/ethyl acetate 30:1 (v/v) as the eluent to afford products **2**. The yields were determined by ^19^F NMR spectroscopy and the ee values were determined by chiral HPLC.

## Supporting Information

File 1 Full experimental details and characterization data of all compounds.
