# Supplementary material for: Chiral phosphoric acid-catalyzed transfer hydrogenation of 3,3-difluoro-3H-indoles
Source: Beilstein J Org Chem. 2024 Feb 1;20:205–11. doi: 10.3762/bjoc.20.20 (PMC10840539; doi:10.3762/bjoc.20.20)

BEILSTEIN JOURNAL OF ORGANIC CHEMISTRY

Supporting Information

for

Chiral phosphoric acid-catalyzed transfer hydrogenation of 3,3-difluoro-3*H*-indoles

Yumei Wang, Guangzhu Wang, Yanping Zhu and Kaiwu Dong

Beilstein J. Org. Chem. **2024,** *20,* 205–211. doi:10.3762/bjoc.20.20

Full experimental details and characterization data of all compounds

License and Terms: This is a supporting information file under the terms of the Creative Commons Attribution License (https://creativecommons.org/licenses/by/4.0). Please note that the reuse, redistribution and reproduction in particular requires that the author(s) and source are credited and that individual graphics may be subject to special legal provisions.
The license is subject to the *Beilstein Journal of Organic Chemistry* terms and conditions: (https://www.beilstein-journals.org/bjoc/terms)

Table of contents

1. General information S2

2. General experimental procedures for 3,3-difluoro-substituted 3*H*-indoles S3

3. General experimental procedure for asymmetric reduction..................................S11

4. References S31

5. NMR spectra copies S32

1. General information

All oxygen- and moisture-sensitive manipulations were carried out under an inert N_2 atmosphere using standard Schlenk techniques or glovebox. DMSO, MeOH, toluene, and DCE were distilled from CaH_2 prior to use. All other chemicals and solvents were used as received. ^{1}H NMR, ^{13}C NMR, and ^{19}F NMR spectra were recorded on Bruker DRX500 and Varian 600 NMR spectrometers at ambient temperature with $CDCl_3$ as solvent. ^{13}C shifts were obtained with ^{1}H decoupling. Chemical shifts and coupling constants are listed in ppm and Hz, respectively. High-resolution mass spectra (HRMS) were recorded on a Bruker microTof by using ESI method. The mass analyzer for the HRMS measurements was "time-of-flight" type. Optical rotation was determined using $CHCl_3$ and as the solvent on INESA WZZ-3. Enantiomeric ratios were determined by chiral HPLC (SHIMADZU LC-20) with *n*-hexane and iPrOH as solvents. Melting points were recorded using the SGWX micromelting point apparatus.

2. General experimental procedures of preparing 3,3-difluoro-substituted *3H*-indole

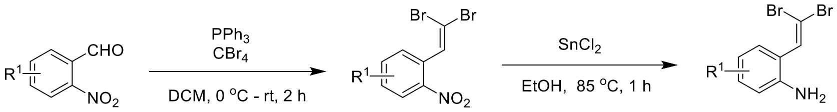

2-Nitrobenzaldehyde (30.0 mmol, 1.0 equiv) was dissolved in DCM (100.0 mL), and then PPh_3 (23.6 g, 90.0 mmol, 3.0 equiv) was added and the resulting solution was cooled to 0 °C in an ice bath. CBr_4 (13.3 g, 45.0 mmol, 1.5 equiv) was added in several portions, and the solution was stirred at 0 °C until the reaction was complete by TLC (2 h). Diatomaceous earth filtration, solvent removal under reduced pressure, and direct use of crude products.

To an EtOH (100.0 mL) solution of 1-(2,2-dibromovinyl)-2-nitrobenzene (30.0 mmol, 1.0 equiv) was added tin(II) chloride (28.4 g, 150.0 mmol, 5.0 equiv). The reaction was refluxed for 1 h. The cooled mixture was filtered through a pad of celite after neutralization with a saturated potassium carbonate solution to pH 10. The organic layer was separated and the aqueous layer was extracted with AcOEt. The combined organic layer was dried over $MgSO_4$. After the removal of solvent, the residue was purified by column chromatography using petroleum ether/ethyl acetate 30: 1 (v/v) as the eluent to afford the corresponding aniline.[1]

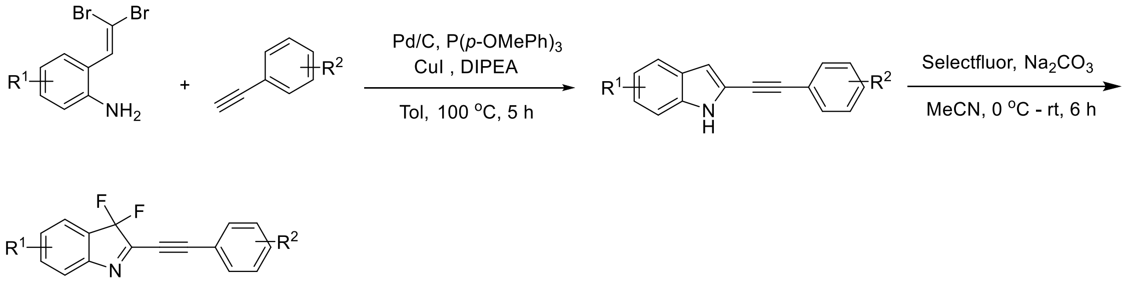

A 100 mL round-bottomed flask was charged with 10% Pd/C (265.0 mg, 0.25 mmol, 2.5 mol %) and tris(*p*-methoxyphenyl)phosphine (3.5 g, 1.0 mmol, 10 mol %), and the flask was purged with argon for at least 10 min. A second separate flask was charged with 2-(2,2-dibromovinyl)aniline (10.0 mmol, 1.0 equiv) and CuI (190.4 mg, 1.0 mmol, 10 mol %), and the flask was purged with argon for 10 min, followed by addition of toluene (30.0 mL), phenylacetylene (15.0 mmol, 1.5 equiv), and iPr_2NH (2.0 g, 20.0

mmol, 2.0 equiv). After the mixture in the second flask became homogenous, it was cannulated into the first flask, and the resulting mixture was heated at 100 °C for 5 h. The reaction mixture was diluted with EtOAc (50 mL × 3) and H_2O (50 mL). The combined organic solution was dried over anhydrous $MgSO_4$. After the removal of solvent, the residue was purified by column chromatography using petroleum ether/EtOAc (30: 1, v/v) as the eluent to afford the corresponding indoles.[2]

To a solution of the 2-alkynylindole (5.0 mmol, 1.0 equiv) in CH_3CN (30.0 mL) was added Na_2CO_3 (529.9 mg, 5.0 mmol, 1.0 equiv) and Selectfluor (3.9 g, 11.0 mmol, 2.2 equiv) at 0 ºC. The mixture was stirred at this temperature for 6 h until the full consumption of 2-alkynylindole. When the reaction was complete, DCM (100.0 mL) was added and the mixture was washed with H_2O (3 × 20 mL) and brine (3 × 15 mL). The organic extracts were dried over Na_2SO_4, and the solvent was concentrated in vacuo. The resulting residue was directly subjected to column chromatography using petroleum ether/EtOAc 30: 1 (v/v) as the eluent to afford 2-alkynyl-3,3-difluoro-3*H*-indoles.[3]

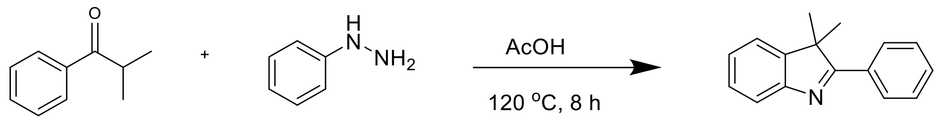

A 250-mL round-bottomed flask was charged with 2-methyl-1-phenylpropan-1-one (1.5 g, 10.0 mmol, 1.0 equiv), AcOH (50.0 mL) and phenylhydrazine (1.1 g, 15.0 mmol, 1.5 equiv). The resulting mixture was heated to 120 ºC for 16 h. The reaction mixture was poured onto saturated aqueous Na_2CO_3 and the crude product was extracted with DCM (3 × 100 mL). The combined organic layers were dried with Na_2SO_4, filtered, concentrated. The resulting residue was directly subjected to column chromatography using petroleum ether/EtOAc 30: 1 (v/v) as the eluent to afford 3,3-dimethyl-2-phenyl-3*H*-indole in 89% yield.[4]

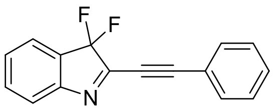

3,3-Difluoro-2-(phenylethynyl)-3*H*-indole (1a): Yellow solid, mp 76-78 ℃, 1.13 g, 89% yield. (CAS: 2242812-89-9).[3] **^{1}H NMR** (600 MHz, $CDCl_3$) δ 7.68 –7.66 (m, 2H), 7.54 – 7.51 (m, 3H), 7.50 – 7.46 (m, 1H), 7.43 – 7.40 (m, 2H), 7.35 – 7.33 (m, 1H). **^{13}C NMR** (151 MHz, $CDCl_3$) δ 157.2 (t, $J_{C\text{-}F}$ = 27.5 Hz),152.6 (t, $J_{C\text{-}F}$ = 8.9 Hz), 133.3, 132.7, 130.6, 128.5, 128.4, 127.2 (t, $J_{C\text{-}F}$ = 23.7 Hz), 123.3, 122.5, 120.5 (t, $J_{C\text{-}F}$ = 255.0 Hz), 120.5, 103.3, 80.7. **^{19}F NMR** (565 MHz, $CDCl_3$) δ -122.43.

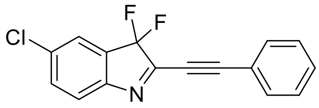

5-Chloro-3,3-difluoro-2-(phenylethynyl)-3*H*-indole (1b): Yellow solid, mp 100-102 ℃, 1.26 g, 88% yield. (CAS:2271165-31-0).[5] **^{1}H NMR** (600 MHz, $CDCl_3$) δ 7.64 (d, J = 7.2 Hz, 2H), 7.48 – 7.39 (m, 6H). **^{13}C NMR** (151 MHz, $CDCl_3$) δ 157.3 (t, $J_{C\text{-}F}$ = 27.0 Hz), 151.0 (t, $J_{C\text{-}F}$ = 8.4 Hz), 134.5, 133.2, 132.8, 130.8, 128.8 (t, $J_{C\text{-}F}$ = 24.0 Hz), 128.6, 124.1, 123.3, 120.2, 120.0 (t, $J_{C\text{-}F}$ = 256.5 Hz), 104.1, 80.5. **^{19}F NMR** (565 MHz, $CDCl_3$) δ -121.93.

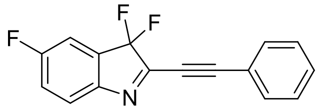

3,3,5-Trifluoro-2-(phenylethynyl)-3*H*-indole (1c): Yellow solid, mp 105-107 ℃, 1.08 g, 80% yield. (CAS:2242813-00-7)[3]. **^{1}H NMR** (600 MHz, $CDCl_3$) δ 7.63 (d, J = 7.2 Hz, 2H), 7.45 – 7.43 (m, 2H), 7.40 – 7.37 (m, 2H), 7.22 (d, J = 6.6 Hz,1H), 7.17 – 7.14 (m,1H). **^{13}C NMR** (151 MHz, $CDCl_3$) δ 162.6 (d, $J_{C\text{-}F}$ = 249.6 Hz), 157.1 (td, $J_{C\text{-}F}$ = 26.9, 4.5 Hz), 148.4 (td, $J_{C\text{-}F}$ = 8.4, 3.2 Hz), 132.7, 130.7, 129.1 (td, $J_{C\text{-}F}$ = 24.0, 8.9 Hz), 128.5, 123.6 (d, $J_{C\text{-}F}$ = 8.3 Hz), 120.3, 119.8 (t, $J_{C\text{-}F}$ = 255.0 Hz), 119.5 (d, $J_{C\text{-}F}$ = 23.4 Hz), 111.9 (d, $J_{C\text{-}F}$ = 26.3 Hz), 103.4, 80.4. **^{19}F NMR** (565 MHz, $CDCl_3$) δ -111.18, -122.29.

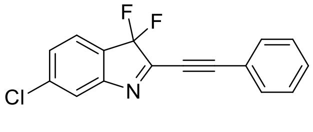

6-Chloro-3,3-difluoro-2-(phenylethynyl)-3*H*-indole (1d): Yellow solid, mp 106-

108 ℃, 1.16 g, 81% yield. (CAS:2253620-16-3).[3] **^{1}H NMR** (600 MHz, $CDCl_3$) δ 7.68 – 7.66 (m, 3H), 7.50 – 7.47 (m, 2H), 7.43 – 7.38 (m, 3H). **^{13}C NMR** (151 MHz, $CDCl_3$) δ 158.6 (t, $J_{C\text{-}F}$ = 27.2 Hz), 154.0 (t, $J_{C\text{-}F}$ = 8.7 Hz), 133.0, 131.2, 131.0, 128.7, 127.4, 126.1 (t, $J_{C\text{-}F}$ = 23.9 Hz), 126.0, 124.4, 120.2, 120.0 (t, $J_{C\text{-}F}$ = 255.7 Hz), 104.6, 80.6. **^{19}F NMR** (565 MHz, $CDCl_3$) δ -121.79.

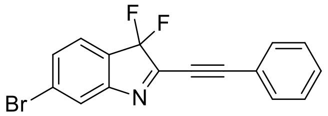

6-Bromo-3,3-difluoro-2-(phenylethynyl)-3*H*-indole (1e): Yellow solid, mp 84-86 ℃, 1.15 g, 76% yield. **^{1}H NMR** (600 MHz, $CDCl_3$) δ 7.68 – 7.66 (m, 2H), 7.50 – 7.40 (m, 5H), 7.31 (dd, *J* = 7.8, 1.8 Hz, 1H). **^{13}C NMR** (150 MHz, $CDCl_3$) δ 158.7 (t, $J_{C\text{-}F}$ = 27.3 Hz), 154.0 (t, $J_{C\text{-}F}$ = 9.0 Hz), 139.4, 132.9, 130.9, 128.7, 128.2, 125.6 (t, $J_{C\text{-}F}$ = 24.1 Hz), 124.1, 123.2, 120.2, 119.8 (t, $J_{C\text{-}F}$ = 255.3 Hz), 104.6, 80.6. **^{19}F NMR** (565 MHz, $CDCl_3$) δ -121.60. HRMS (ESI) m/z: $[M+H]^+$ Calcd for $C_{17}H_{11}{}^{79}BrF_2N$, 331.9981; Found 331.9965.

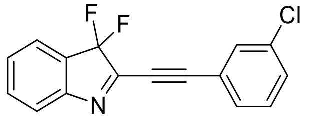

2-((3-Chlorophenyl)ethynyl)-3,3-difluoro-*3*H-indole (1f): Yellow solid, mp 70-72 ℃, 1.22 g, 85% yield. (CAS: 2242812-93-5).[3] **^{1}H NMR** (600 MHz, $CDCl_3$) δ 7.65 (t, *J* = 1.8 Hz, 1H), 7.56 – 7.50 (m, 4H), 7.46 – 7.44 (m, 1H), 7.37 – 7.34 (m, 2H). **^{13}C NMR** (151 MHz, $CDCl_3$) δ 157.0 (t, $J_{C\text{-}F}$ = 27.3 Hz), 152.5 (t, $J_{C\text{-}F}$ = 8.7 Hz), 134.5, 133.4, 132.4, 130.9, 130.8, 129.8, 128.7, 127.2 (t, $J_{C\text{-}F}$ = 24.0 Hz), 123.4, 122.7, 122.1, 120.4 (t, $J_{C\text{-}F}$ = 255.0 Hz), 101.1, 81,4. **^{19}F NMR** (565 MHz, $CDCl_3$) δ -122.66.

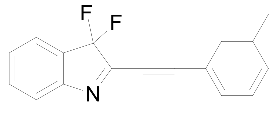

3,3-Difluoro-2-(*m*-tolylethynyl)-3*H*-indole (1g): Yellow solid, mp 52-53 ℃, 1.13 g, 85% yield. **^{1}H NMR** (600 MHz, $CDCl_3$) δ 7.53 – 7.47 (m, 5H), 7.35 – 7.27 (m, 3H), 2.38 (s, 3H). **^{13}C NMR** (151 MHz, $CDCl_3$) δ 157.0 (t, $J_{C\text{-}F}$= 27.0 Hz), 152.4 (t, $J_{C\text{-}F}$= 8.9 Hz), 138.1, 133.1, 132.9, 131.4, 129.6, 128.2, 128.1, 127.0 (t, $J_{C\text{-}F}$ = 23.7 Hz), 123.1, 122.2, 120.3 (t, $J_{C\text{-}F}$ = 255.0 Hz), 120.0, 103.5, 80.3, 20.7. **^{19}F NMR** (565 MHz, $CDCl_3$) δ -122.36. HRMS (ESI) m/z: $[M+Na]^+$ Calcd for $C_{17}H_{11}F_2NNa$, 290.0752; Found

290.0750.

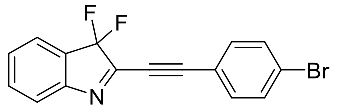

2-((4-Bromophenyl)ethynyl)-3,3-difluoro-3*H*-indole (1h): Yellow solid, mp 108-110 ℃, 1.21 g, 73% yield. **^{1}H NMR** (500 MHz, $CDCl_3$) δ 7.53 – 7.46 (m, 7H), 7.33 – 7.30 (m, 1H). **^{13}C NMR** (125 MHz, $CDCl_3$) δ 157.0 (t, $J_{C\text{-}F}$ = 27.3 Hz), 152.5 (t, $J_{C\text{-}F}$ =8.9 Hz), 133.9, 133.4, 131.9, 128.5, 127.1 (t, $J_{C\text{-}F}$ = 27.6 Hz), 125.4, 123.3, 122.6, 120.4 (t, $J_{C\text{-}F}$ = 255.1 Hz), 119.3, 101.8, 81.6. **^{19}F NMR** (565 MHz, $CDCl_3$) δ -122.63. HRMS (ESI) m/z: $[M+H]^+$ Calcd for $C_{16}H_9{}^{79}BrF_2N$, 331.9881; Found 331.9873.

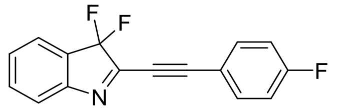

3,3-Difluoro-2-((4-fluorophenyl)ethynyl)-3*H*-indole (1i): Yellow solid, mp 104-106 ℃, 1.09 g, 81% yield. (CAS:2242812-91-3).[3] **^{1}H NMR** (600 MHz, $CDCl_3$) δ 7.67 – 7.63 (m, 2H), 7.52 – 7.48 (m, 3H), 7.34 – 7.31 (m, 1H), 7.12 – 7.08 (m, 2H). **^{13}C NMR** (151 MHz, $CDCl_3$) δ 163.9 (d, $J_{C\text{-}F}$= 252.6 Hz), 157.1 (t, $J_{C\text{-}F}$ = 27.3 Hz), 152.6 (t, $J_{C\text{-}F}$ = 8.9 Hz), 135.0 (d, $J_{C\text{-}F}$ = 9.2 Hz), 133.4, 128.5, 127.2 (t, $J_{C\text{-}F}$ = 23.4 Hz), 123.4, 122.5, 120.4 (t, $J_{C\text{-}F}$ = 255.1 Hz), 116.6 (d, $J_{C\text{-}F}$ = 3.3 Hz), 116.1 (t, $J_{C\text{-}F}$ = 22.2 Hz), 102.1, 80.6. **^{19}F NMR** (565 MHz, $CDCl_3$) δ -106.17, -122.43.

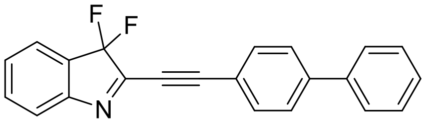

2-([1,1'-Biphenyl]-4-ylethynyl)-3,3-difluoro-3*H*-indole (1j): Yellow solid, mp 129-131 ℃, 1.41 g, 86% yield. **^{1}H NMR** (600 MHz, $CDCl_3$) δ 7.70 - 7.69 (m, 2H), 7.61 - 7.57 (m, 4H), 7.51 - 7.42 (m, 5H), 7.37 -7.35 (m, 1H), 7.30 – 7.28 (m, 1H). **^{13}C NMR** (151 MHz, $CDCl_3$) δ 157.2 (t, $J_{C\text{-}F}$ = 27.2 Hz), 152.6 (t, $J_{C\text{-}F}$ = 8.7 Hz), 143.3, 139.6, 133.3, 133.2, 128.9, 128.4, 128.1, 127.2 (t, $J_{C\text{-}F}$ = 23.7 Hz), 127.1, 127.0, 123.3, 122.5, 120.4(t, $J_{C\text{-}F}$ = 255.0 Hz), 119.1, 103.4, 81.5. **^{19}F NMR** (565 MHz, $CDCl_3$) δ -122.31. HRMS (ESI) m/z: $[M+H]^+$ Calcd for $C_{22}H_{14}F_2N$,330.1085; Found 330.1089

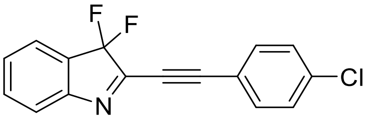

2-((4-Chlorophenyl)ethynyl)-3,3-difluoro-3*H*-indole (1k): Yellow solid, mp 108-110 ℃, 1.12 g, 78% yield. (CAS:2242812-92-4).[3] **^{1}H NMR** (600 MHz, $CDCl_3$) δ 7.59 – 7.56 (m, 2H), 7.52 – 7.48 (m, 3H), 7.39 – 7.36 (m, 2H), 7.34 – 7.31 (m, 1H). **^{13}C NMR** (151 MHz, $CDCl_3$) δ 157.0 (t, $J_{C\text{-}F}$= 27.6 Hz), 152.5 (t, $J_{C\text{-}F}$ = 8.9 Hz), 137.0, 133.9, 133.4, 129.0, 128.6, 127.2 (t, $J_{C\text{-}F}$ = 23.8 Hz), 123.4, 122.6, 120.4 (t, $J_{C\text{-}F}$ = 255.0 Hz), 118.9, 101.8, 81.5. **^{19}F NMR** (565 MHz, $CDCl_3$) δ -122.53.

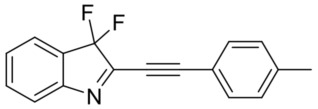

3,3-Difluoro-2-(*p*-tolylethynyl)-3*H*-indole (1l): Yellow solid, mp 99-101 ℃, 1.07 g, 80% yield. (CAS:2242812-90-2).[3] **^{1}H NMR** (600 MHz, $CDCl_3$) δ 7.56 (d, J = 8.4 Hz, 2H), 7.53 – 7.50 (m, 3H), 7.35 – 7.31 (m, 1H), 7.22 (d, J = 8.4 Hz, 2H), 2.41(s, 3H). **^{13}C NMR** (151 MHz, $CDCl_3$) δ 157.3 (t, $J_{C\text{-}F}$ = 27.3 Hz), 152.7 (t, $J_{C\text{-}F}$ = 8.7 Hz), 141.4, 133.3, 132.7, 129.3, 128.3, 127.2 (t, $J_{C\text{-}F}$ = 23.9 Hz), 123.3, 122.4, 120.4 (t, $J_{C\text{-}F}$ = 254.9 Hz), 117.3, 103.9, 80.5, 21.6. **^{19}F NMR** (565 MHz, $CDCl_3$) δ -122.23.

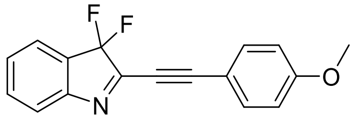

3,3-Difluoro-2-((4-methoxyphenyl)ethynyl)-3*H*-indole(1m): Yellow solid, mp 119-121 ℃, 1.06 g, 75% yield. **^{1}H NMR** (600 MHz, $CDCl_3$) δ 7.61 – 7.58 (m, 2H), 7.51 – 7.47 (m, 3H), 7.31 – 7.28 (m, 1H), 6.91 – 6.90 (m, 2H), 3.82 (s, 3H). **^{13}C NMR** (151 MHz, $CDCl_3$) δ 161.6, 157.3 (t, $J_{C\text{-}F}$ = 27.3 Hz), 152.8 (t, $J_{C\text{-}F}$ = 9.2 Hz), 134.7, 133.3, 128.1, 127.2 (t, $J_{C\text{-}F}$ = 23.4 Hz), 123.3, 122.3, 120.5 (t, $J_{C\text{-}F}$ = 255.0 Hz), 114.3, 112.3, 104.4, 80.4, 55.3. **^{19}F NMR** (565 MHz, $CDCl_3$) δ -121.84. HRMS (ESI) m/z: $[M+H]^+$ Calcd for $C_{17}H_{12}F_2NO$, 284.0881; Found 284.0885.

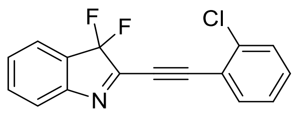

2-((2-Chlorophenyl)ethynyl)-3,3-difluoro-3*H*-indole (1n): Yellow solid, mp 120-121 ℃, 1.18 g, 82% yield. (CAS: 2242812-94-6).[3] **^{1}H NMR** (600 MHz, $CDCl_3$) δ 7.66 – 7.64 (dd, J = 7.8, 1.8 Hz, 1H), 7.53 – 7.48 (m, 3H), 7.46 – 7.45 (m, 1H), 7.39 – 7.36 (m, 1H), 7,35 – 7.32 (m, 1H), 7.29 – 7.27 (m, 1H). **^{13}C NMR** (151 MHz, $CDCl_3$) δ 157.0 (t, $J_{C\text{-}F}$ = 27.8 Hz), 152.5 (t, $J_{C\text{-}F}$ = 8.6 Hz), 137.2, 134.3, 133.4, 131.6, 129.6,

128.6, 127.3 (t, J_{C-F} = 23.6 Hz), 126.6, 123.4, 122.7, 120.7, 120.4 (t, J_{C-F} = 255.1 Hz), 99.4, 85.0. **^{19}F NMR** (565 MHz, $CDCl_3$) δ -122.59.

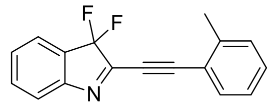

3,3-Difluoro-2-(*o*-tolylethynyl)-3*H*-indole (1o): Yellow solid, mp 95-97 ℃, 1.17 g, 88% yield. **^{1}H NMR** (600 MHz, $CDCl_3$) δ 7.61 (d, J = 7.8 Hz, 1H), 7.52 – 7.48 (m, 3H), 7.36 – 7.30 (m, 2H),7.27 (d, J = 7.8 Hz, 1H), 7.21 (t, J = 7.8 Hz, 1H), 2.56 (s, 3H). **^{13}C NMR** (150 MHz, $CDCl_3$) δ 157.4 (t, J_{C-F} =27.3 Hz), 152.8 (t, J_{C-F} = 8.7 Hz), 142.1, 133.4, 133.2, 130.8, 129.8, 128.3, 127.4 (t, J_{C-F} = 23.7 Hz), 125.8, 123., 122.5, 120.5 (t, J_{C-F} = 255.0 Hz), 120.3, 102.9, 84.5, 20.5. **^{19}F NMR** (565 MHz, $CDCl_3$) δ -122.18. HRMS (ESI) m/z: $[M+Na]^+$ Calcd for $C_{17}H_{11}F_2NNa$, 290.0752; Found 290.0753.

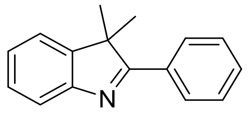

3,3-Dimethyl-2-phenylindoline (1p): Yellow oil, 1.9 g, 89% yield. (CAS:6636-32-4).[4] **^{1}H NMR** (600 MHz, $CDCl_3$) δ 8.15 – 8.14 (m, 2H), 7.70 (d, J = 7.8 Hz, 1H), 7.49 – 7.48 (m, 3H), 7.38 – 7.33 (m, 2H), 7.28 - 7.26 (m, 1H), 1.59 (s, 6H). **^{13}C NMR** (150 MHz, $CDCl_3$) δ 183.2, 153.0, 147.6, 133.3, 130.5, 128.6, 128.3, 127.7, 125.8, 120.9, 120.9, 53.5, 24.7.

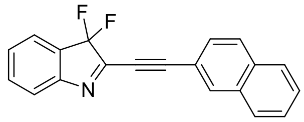

3,3-Difluoro-2-(naphthalen-2-ylethynyl)-3*H*-indole (1q): Yellow solid, mp 104-106 ℃, 1.15 g, 76% yield. (CAS: 2242812-95-7).[3] **^{1}H NMR** (600 MHz, $CDCl_3$) δ 8.23 (s, 1H), 7.87 - 7.84 (m, 3H), 7.66 -7.65 (m, 1H), 7.58 – 7.50 (m, 5H), 7.35 - 7.33 (m, 1H). **^{13}C NMR** (150 MHz, $CDCl_3$) δ 157.4 (t, J_{C-F} = 27.3 Hz), 152.8 (t, J_{C-F} = 8.7 Hz), 134.1, 133.9, 133.4, 132.7, 128.5, 128.4, 128.3, 128.2, 128.0, 127.9, 127.4 (t, J_{C-F} = 23.6 Hz), 127.0, 123.4, 122.6, 120.5 (t, J_{C-F} = 255.2 Hz), 117.7, 103.9, 81.1. **^{19}F NMR** (565 MHz, CDCl3) δ -122.27

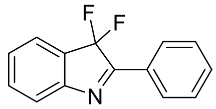

3,3-Difluoro-2-phenyl-3*H*-indole (1r): Yellow solid, mp 79-81 ℃, 0.99 g, 86% yield. (CAS: 1322089-48-4).[6] **^{1}H NMR** (600 MHz, $CDCl_3$) δ 8.20 (d, J = 7.8 Hz, 2H), 7.53 – 7.44 (m, 6H), 7.25 – 7.23 (m, 1H). **^{13}C NMR** (150 MHz, $CDCl_3$) δ 169.1 (t, J_{C-F} = 24.6 Hz), 152.5 (t, J_{C-F} = 9.6 Hz), 133.2, 132.4, 128.8 (t, J_{C-F} = 24.0 Hz), 128.8, 128.5, 127.4, 123.0 (t, J_{C-F} = 254.7 Hz), 122.9, 121.9. **^{19}F NMR** (565 MHz, $CDCl_3$) δ -116.39.

3. General experimental procedure for asymmetric reduction

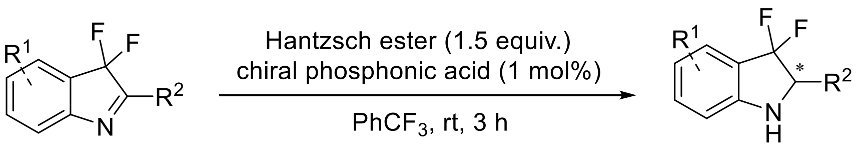

A 4-mL sample bottle was charged with 3,3-difluoro substituted 3*H*-indole **1** (0.1 mmol, 1.0 equiv), Hantzsch ester (**HE-*t*-Bu**42.0 mg, 0.15 mmol, 1.5 equiv), chiral phosphonic acid (**CPA-6**, 0.75 mg, 0.001 mmol, 1.0 mol %). Then, $PhCF_3$ (1 mL) was added in the glove box under N_2 atmosphere. The reaction was stirred at room temperature for 3 h. After concentrating the mixture, the residue was purified by column chromatography on silica gel using the mixture of petroleum ether/ethyl acetate 30:1 (v/v) as the eluent to afford products **2**. The yields were determined by ^{19}F NMR spectroscopy and the ee values were determined by chiral HPLC.

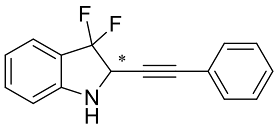

3,3-Difluoro-2-(phenylethynyl)indoline (2a): Yellow solid, mp 66-67 ℃, 99% yield, 96% ee. $[\alpha]_D^{25}$ = -53.867 (*c* = 0.5, $CHCl_3$). **^{1}H NMR** (600 MHz, $CDCl_3$) δ 7.49 – 7.45 (m, 3H), 7.34 – 7.28 (m, 4H), 6.93 – 6.91 (m, 1H), 6.78 (d, *J* = 6.0 Hz, 1H), 4.89 – 4.85 (m, 1H), 4.26 (s, 1H). **^{13}C NMR** (151 MHz, $CDCl_3$) δ 149.9 (t, J_{C-F} = 6.5 Hz), 133.0, 131.9, 128.8, 128.3, 125.0 (t, J_{C-F} = 250.5 Hz), 124.1, 121.9, 121.0 (t, J_{C-F} = 25.2 Hz), 120.4, 112.1, 87.4, 81.5 (t, J_{C-F} = 6.6 Hz), 57.7 (dd, J_{C-F} = 36.0, 11.0 Hz). **^{19}F NMR** (565 MHz, $CDCl_3$) δ -87.83 (d, *J* = 247.5 Hz), -89.83 (d, *J* = 246.9 Hz). HRMS (ESI) m/z: $[M+H]^+$ Calcd for $C_{16}H_{12}F_2N$, 256.0932; Found 256.0914. The enantiomeric ratio was determined by HPLC analysis on Daicel Chiralpak OD-3 column. *n*-Hexane/*i*-PrOH = 98:2, flow rate = 1.0 mL/min., λ = 254 nm, t_R = 24.7 min. (minor), 28.9 min. (major).

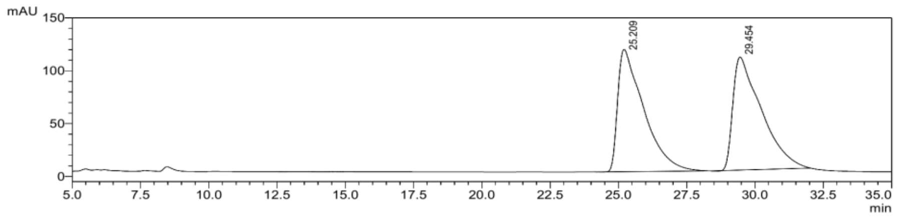

PDA Ch1 240nm

Peak#	Ret. Time	Height	Area	Area%
1	25.209	115765	7678663	49.642
2	29.454	106929	7789444	50.358
总计		222693	15468107	100.000

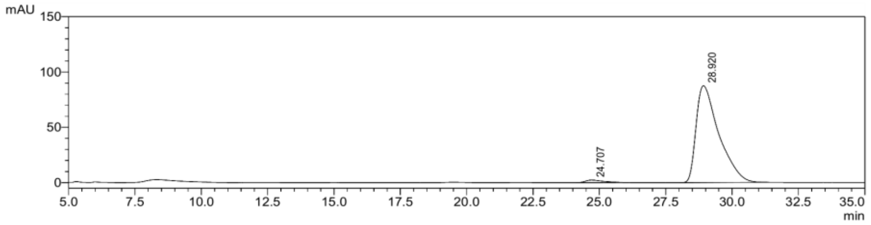

PDA Ch1 240nm

Peak#	Ret. Time	Height	Area	Area%
1	24.707	2331	111571	2.089
2	28.920	87534	5229133	97.911
总计		89865	5340704	100.000

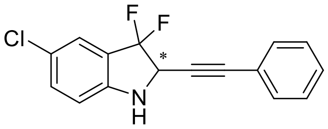

5-Chloro-3,3-difluoro-2-(phenylethynyl)indoline (2b): Yellow solid, mp 90-92 ℃, 96% yield, 94% ee. $[\alpha]_D^{25}$ = -82.267 (c = 0.5, $CHCl_3$)**.** ^{1}**H NMR** (600 MHz, $CDCl_3$) δ 7.46 – 7.45 (m, 3H), 7.34 – 7.28 (m, 4H), 6.73 – 6.71 (m, 1H), 4.93 – 4.88 (m, 1H), 4.27 (s, 1H). ^{13}**C NMR** (151 MHz, $CDCl_3$) δ 148.4 (t, $J_{C\text{-}F}$ = 6.6 Hz),133.0, 131.9, 129.0, 128.3, 125.1, 124.2 (t, $J_{C\text{-}F}$ = 251.7 Hz), 124.2, 122.4 (t, $J_{C\text{-}F}$ = 25.4 Hz), 121.7, 113.2, 87.8, 80.9 (t, $J_{C\text{-}F}$ = 7.1 Hz), 58.1 (dd, $J_{C\text{-}F}$ = 35.9, 11.1 Hz). ^{19}**F NMR** (565 MHz, $CDCl_3$) -87.98 (d, J = 247.5 Hz), -90.50 (J = 248.0 Hz). HRMS (ESI) m/z: $[M+H]^+$ Calcd for $C_{16}H_{11}{}^{35}ClF_2N$, 290.0543; Found 290.0544. The enantiomeric ratio was determined by HPLC analysis on Daicel Chiralpak OD-3 column. n-Hexane/i-PrOH = 98:2, flow rate = 1.0 mL/min., λ = 254 nm, t_R = 31.3 min. (minor), 53.5 min. (major).

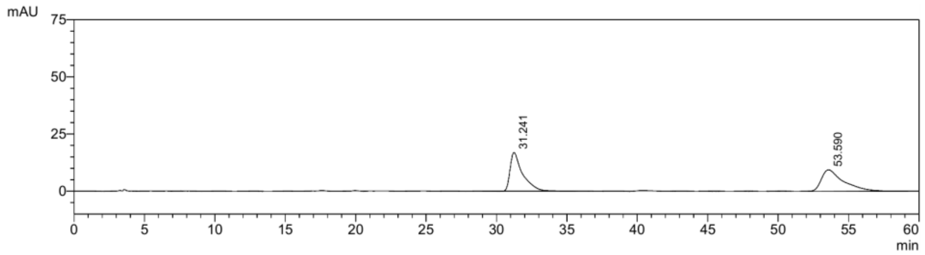

PDA Ch1 250nm

Peak#	Ret. Time	Height	Area	Area%
1	31.241	16882	1028073	49.944
2	53.590	9405	1030386	50.056
总计		26288	2058459	100.000

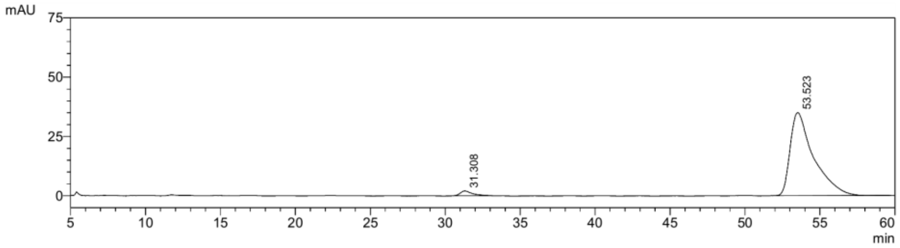

PDA Ch1 250nm

Peak#	Ret. Time	Height	Area	Area%
1	31.308	2028	118176	3.009
2	53.523	35000	3808647	96.991
总计		37027	3926822	100.000

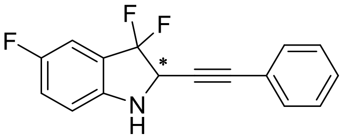

3,3,5-Trifluoro-2-(phenylethynyl)indoline (2c): Yellow solid, mp 57-59 ℃, 97% yield, 90% ee. $[\alpha]_D^{25}$ = -42.733 (c = 0.5, $CHCl_3$)**.** **^{1}H NMR** (600 MHz, $CDCl_3$) δ 7.47 – 7.45 (m, 2H), 7.34 – 7.29 (m, 3H), 7.19 – 7.17 (m, 1H), 7.08 – 7.05 (m, 1H), 6.75 – 6.73 (m, 1H), 4.93 – 4.88 (m, 1H), 4.17 (s, 1H). **^{13}C NMR** (151 MHz, $CDCl_3$) δ 157.4 (t, $J_{C\text{-}F}$ = 238.1 Hz), 146.0 (t, $J_{C\text{-}F}$ = 6.9 Hz), 131.9, 128.9, 128.3, 124.6 (t, $J_{C\text{-}F}$ = 251.0 Hz,), 122.1 (td, $J_{C\text{-}F}$ =25.2, 8.0 Hz), 121.8, 120.2 (d, $J_{C\text{-}F}$ = 23.7 Hz), 113.3 (d, $J_{C\text{-}F}$ = 7.8 Hz), 110.7 (t, $J_{C\text{-}F}$ = 24.5 Hz), 87.7, 81.1 (t, $J_{C\text{-}F}$ = 6.6 Hz), 58.3 (dd, $J_{C\text{-}F}$ =35.6, 11.0 Hz), **^{19}F NMR** (565 MHz, $CDCl_3$) δ -88.57 (d, J = 246.9 Hz), -90.36 (d, J = 248.6 Hz), -122.82. HRMS (ESI) m/z: $[M+H]^+$ Calcd for $C_{16}H_{11}F_3N$, 274.0838; Found 274.0843. The enantiomeric ratio was determined by HPLC analysis on Daicel Chiralpak OD-3 column. n-Hexane/i-PrOH = 98:2, flow rate = 1.0 mL/min., λ = 254 nm, t_R = 30.7 min. (minor), 45.9 min. (major).

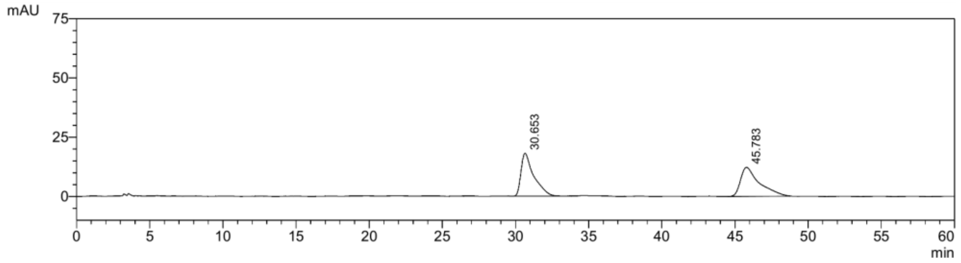

PDA Ch1 239nm

Peak#	Ret. Time	Height	Area	Area%
1	30.653	18062	1093859	49.928
2	45.783	12249	1097016	50.072
总计		30311	2190876	100.000

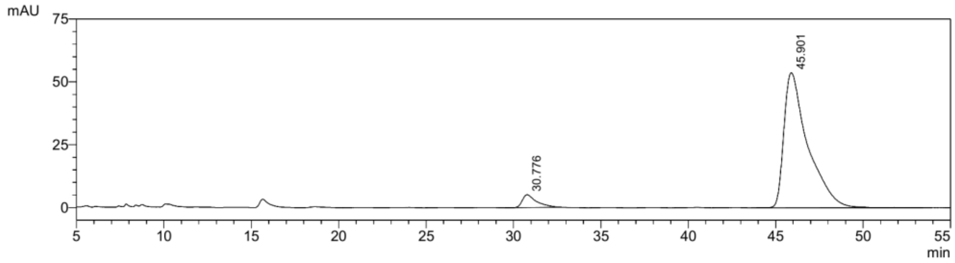

PDA Ch1 240nm

Peak#	Ret. Time	Height	Area	Area%
1	30.776	5101	289209	5.364
2	45.901	53766	5102290	94.636
总计		58866	5391499	100.000

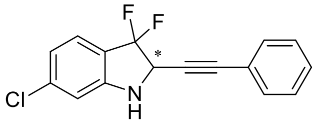

6-Chloro-3,3-difluoro-2-(phenylethynyl)indoline (2d): Yellow solid, mp 51-53 ℃, 96% yield, 95% ee. $[\alpha]_D^{25}$ = -68.037 (c = 0.5, $CHCl_3$)**.** **^{1}H NMR** (600 MHz, $CDCl_3$) δ 7.46 – 7.45 (m, 2H), 7.34 – 7.29 (m, 4H), 7.05 – 7.03 (m, 1H), 6.94 – 6.93 (m, 1H). 4.91 – 4.87(m, 1H). **^{13}C NMR** (151 MHz, $CDCl_3$) δ 151.0 (t, $J_{C\text{-}F}$ = 6.2 Hz), 131.9, 128.9, 128.3, 127.23, 125.4, 124.2 (t, $J_{C\text{-}F}$ = 251.4 Hz), 123.4, 121.7, 119.9 (t, $J_{C\text{-}F}$ = 25.7 Hz), 114.9, 87.8, 80.8 (t, $J_{C\text{-}F}$ = 7.2 Hz), 57.9 (dd, $J_{C\text{-}F}$ =36.6, 11.9 Hz). **^{19}F NMR** (565 MHz, $CDCl_3$) δ -87.37 (d, J = 248.0 Hz), -90.71 (d, J = 248.0 Hz). HRMS (ESI) m/z: $[M+H]^+$ Calcd for $C_{16}H_{11}{}^{35}ClF_2N$, 290.0543; Found 290.0530. The enantiomeric ratio was determined by HPLC analysis on Daicel Chiralpak OD-3 column. n-Hexane/i-PrOH = 98:2, flow rate = 1.0 mL/min., λ = 254 nm, t_R = 25.1 min. (minor), 53.3 min. (major).

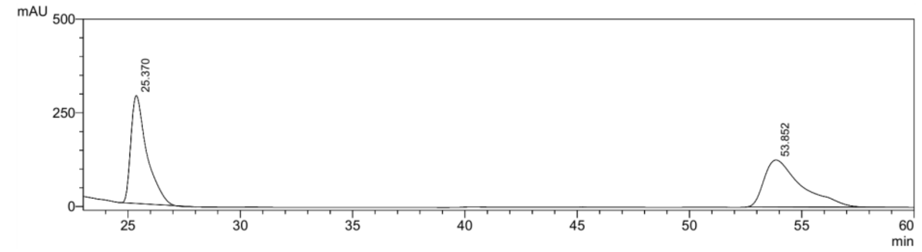

PDA Ch1 239nm

Peak#	Ret. Time	Height	Area	Area%
1	25.370	287809	14208365	49.621
2	53.852	125218	14425193	50.379
总计		413027	28633558	100.000

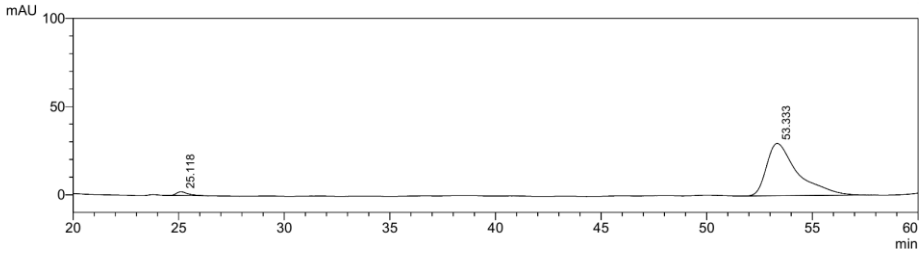

PDA Ch1 254nm

Peak#	Ret. Time	Height	Area	Area%
1	25.118	2105	79224	2.543
2	53.333	29676	3035749	97.457
总计		31780	3114973	100.000

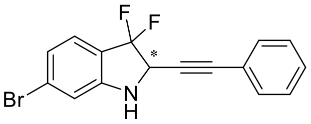

6-Bromo-3,3-difluoro-2-(phenylethynyl)indoline (2e): Yellow solid, mp 41-43 ℃, 97% yield, 93% ee. $[\alpha]_D^{25}$ = -73.840 (c = 0.5, $CHCl_3$). **^{1}H NMR** (600 MHz, $CDCl_3$) δ 7.46 – 7.45 (m, 2H), 7.38 (d, J = 7.8 Hz, 1H), 7.33 – 7.29 (m, 3H), 6.87 (dd, J = 7.8, 1.8 Hz, 1H), 6.76 (d, J = 1.8 Hz, 1H), 4.92 – 4.88 (m, 1H), 3.97 (s, 1H). **^{13}C NMR** (151 MHz, $CDCl_3$) δ 150.9 (t, $J_{C\text{-}F}$ = 6.2 Hz), 138.9, 131.9, 129.0, 128.3, 125.2, 124.2 (t, $J_{C\text{-}F}$ = 250.8 Hz), 121.7, 120.6, 119.4 (t, $J_{C\text{-}F}$ = 25.8 Hz), 112.0, 87.8, 80.9, 57.9 (dd, $J_{C\text{-}F}$ = 36.5, 12.0 Hz). **^{19}F NMR** (565 MHz, $CDCl_3$) δ -87.23 (d, J = 248.0 Hz), -90.42 (d, J = 248.0 Hz). (ESI) m/z: $[M+H]^+$ Calcd for $C_{16}H_{11}{}^{79}BrF_2N$, 334.0037; Found 334.0032. The enantiomeric ratio was determined by HPLC analysis on Daicel Chiralpak OD-3 column. n-Hexane/i-PrOH = 98:2, flow rate = 1.0 mL/min., λ = 254 nm, t_R = 23.6 min. (minor), 48.6 min. (major).

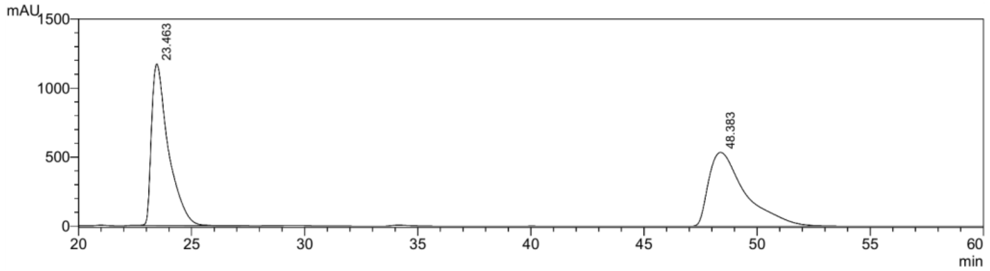

PDA Ch1 241nm

Peak#	Ret. Time	Height	Area	Area%
1	23.463	1171261	60739308	50.307
2	48.383	534238	59998748	49.693
总计		1705499	120738056	100.000

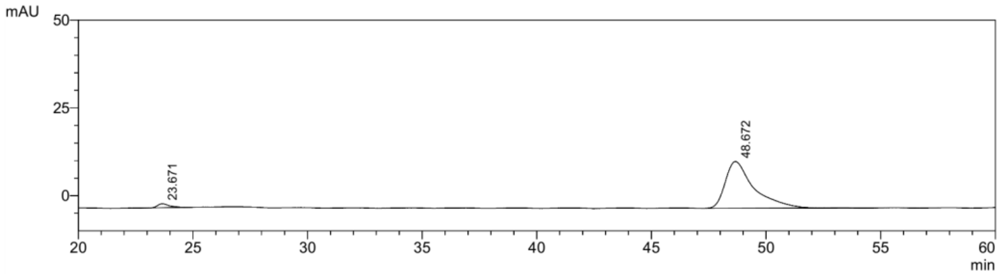

PDA Ch1 254nm

Peak#	Ret. Time	Height	Area	Area%
1	23.671	1117	39844	3.315
2	48.672	13329	1162011	96.685
总计		14446	1201855	100.000

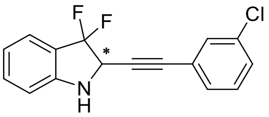

2-((3-Chlorophenyl)ethynyl)-3,3-difluoroindoline (2f): Yellow solid, mp 70-72 ℃, 98% yield, 96% ee. $[\alpha]_D^{25}$ = -67.600 (c = 0.5, $CHCl_3$). **^{1}H NMR** (600 MHz, $CDCl_3$) δ 7.48 – 7.47 (m, 1H), 7.43 (t, J = 1.8 Hz, 1H), 7.34 – 7.28 (m, 3H), 7.23 – 7.20 (m ,1H), 6.92 (t, J = 7.8 Hz, 1H), 6.78 (d, J = 7.8 Hz, 1H), 4.89 – 4.84 (m, 1H), 4.27 (s, 1H). **^{13}C NMR** (151 MHz, $CDCl_3$) δ 149.8 (t, $J_{C\text{-}F}$ = 6.3 Hz), 134.1, 133.0, 131.7, 130.0, 129.5, 129.1, 125.0 (t, $J_{C\text{-}F}$ = 250.5 Hz), 124.1, 123.6, 120.9 (t, $J_{C\text{-}F}$ = 24.9 Hz), 120.5, 112.1, 85.9, 82.8 (t, $J_{C\text{-}F}$ = 6.5 Hz), 57.5 (dd, $J_{C\text{-}F}$ = 35.7, 10.7 Hz). **^{19}F NMR** (565 MHz, $CDCl_3$) δ -87.57 (d, J = 246.9 Hz), -89.70 (d, J = 246.9 Hz). HRMS (ESI) m/z: $[M+H]^+$ Calcd for $C_{16}H_{11}{}^{35}ClF_2N$, 290.0543; Found 290.0540. The enantiomeric ratio was determined by HPLC analysis on Daicel Chiralpak OD-3 column. n-Hexane/i-PrOH = 98:2, flow rate = 0.5 mL/min., λ = 254 nm, t_R = 40.7 min. (minor), 43.3 min. (major).

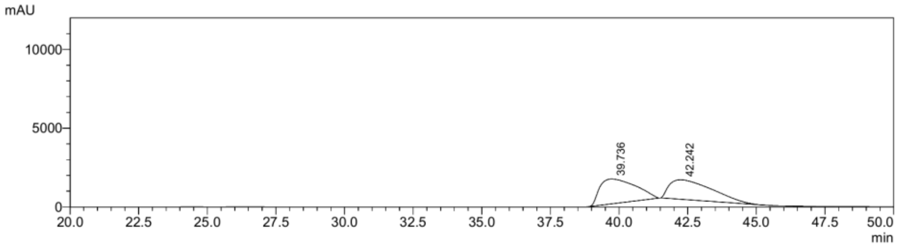

PDA Ch1 254nm

Peak#	Ret. Time	Height	Area	Area%
1	39.736	1575517	141512542	50.762
2	42.242	1229269	137261457	49.238
总计		2804786	278773999	100.000

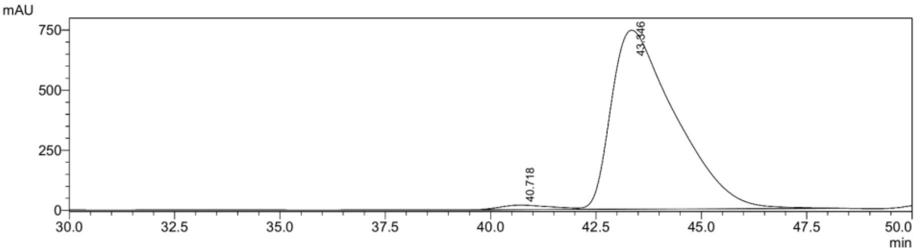

PDA Ch1 241nm

Peak#	Ret. Time	Height	Area	Area%
1	40.718	18923	1617226	2.002
2	43.346	745654	79144457	97.998
总计		764577	80761683	100.000

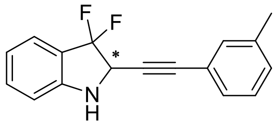

3,3-Difluoro-2-(*m*-tolylethynyl)indoline (2g): Yellow solid, mp 62-64 ℃, 99% yield, 95% ee. $[\alpha]_D^{25}$ = -64.600 (*c* = 0.5, $CHCl_3$)**.** **^{1}H NMR** (600 MHz, $CDCl_3$) δ 7.48 (d, *J* = 7.2 Hz, 1H), 7.33 (t, *J* = 8.4 Hz, 1H), 7.27 – 7.26 (m, 2H), 7.18 (t, *J* = 7.8 Hz, 1H), 7.13 (d, *J* = 7.2 Hz, 1H), 6.92 (t, *J* = 7.2 Hz, 1H), 6.84 (d, *J* = 8.4 Hz, 1H), 4.89 – 4.85 (m, 1H), 4.25 (s, 1H), 2.30 (s, 3H). **^{13}C NMR** (151 MHz, $CDCl_3$) δ 150.0 (t, $J_{C\text{-}F}$ = 6.3 Hz), 138.0, 132.9, 132.5, 129.7, 129.0, 128.2, 125.0 (t, $J_{C\text{-}F}$ =250.5 Hz), 124.1, 121.8, 121.1 (t, $J_{C\text{-}F}$ = 25.2 Hz), 120.4, 112.1, 87.6, 81.1 (t, $J_{C\text{-}F}$ = 6.8 Hz), 57.7 (dd, $J_{C\text{-}F}$ = 35.9, 11.1 Hz), 21.1. **^{19}F NMR** (565 MHz, $CDCl_3$) δ -87.81 (d, *J* = 246.9 Hz), -89.96 (d, *J* = 246.9 Hz). HRMS (ESI) m/z: $[M+H]^+$ Calcd for $C_{17}H_{14}F_2N$, 270.1089; Found 270.1093. The enantiomeric ratio was determined by HPLC analysis on Daicel Chiralpak OD-3 column. *n*-Hexane/*i*-PrOH = 98:2, flow rate = 1.0 mL/min., λ = 254 nm, t_R = 24.6 min. (minor), 27.4 min. (major).

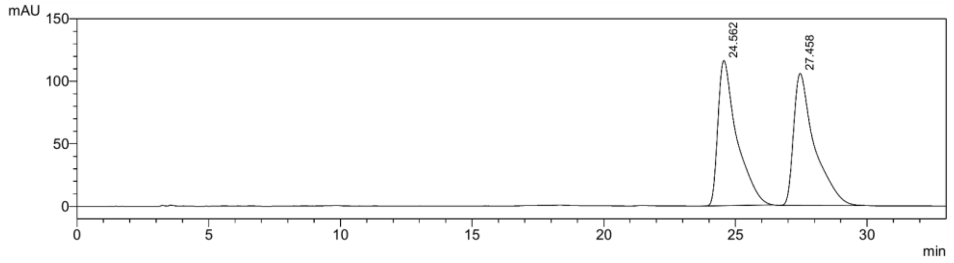

PDA Ch1 242nm

Peak#	Ret. Time	Height	Area	Area%
1	24.562	116108	5796907	49.971
2	27.458	105557	5803747	50.029
总计		221665	11600653	100.000

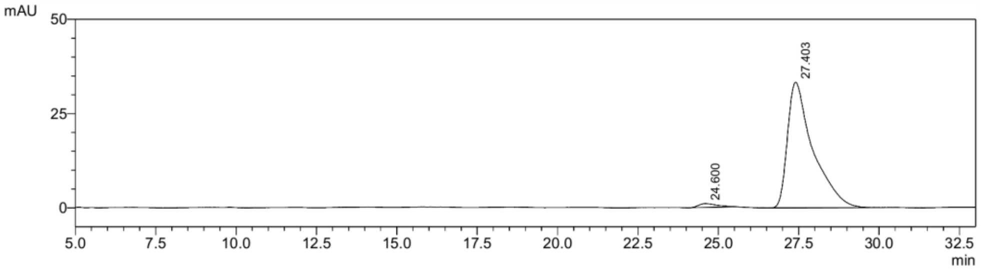

PDA Ch1 242nm

Peak#	Ret. Time	Height	Area	Area%
1	24.600	1022	40524	2.165
2	27.403	33334	1831037	97.835
总计		34356	1871561	100.000

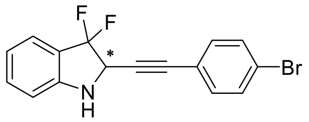

2-((4-Bromophenyl)ethynyl)-3,3-difluoroindoline (2h): Yellow solid, mp 56-58 ℃, 99% yield, 94% ee. $[\alpha]_D^{25}$ = -62.733 (*c* = 0.5, $CHCl_3$)**.** **^{1}H NMR** (600 MHz, $CDCl_3$) δ 7.49 – 7.47 (m, 1H), 7.44 – 7.42 (m, 2H), 7.35 – 7.29 (m, 3H), 6.94 - 6.91 (m, 1H), 6.79 – 6.78 (m, 1H), 4.88 – 4.84 (m, 1H), 4.27 (s, 1H). **^{13}C NMR** (151 MHz, $CDCl_3$) δ 149.9 (t, $J_{C\text{-}F}$ = 6.6 Hz), 133.3, 133.0, 131.6, 124.9 (t, $J_{C\text{-}F}$ = 250.8 Hz), 124.1, 123.2, 120.9 (t, $J_{C\text{-}F}$ = 25.2 Hz), 120.9, 120.5, 112.1, 86.4, 82.7 (t, $J_{C\text{-}F}$ = 6.6 Hz), 57.6 (dd, $J_{C\text{-}F}$ =36, 11.0 Hz). **^{19}F NMR** (565 MHz, $CDCl_3$) δ -87.69 (d, *J* = 246.9 Hz), -89.67 (d, *J* = 247.5 Hz). HRMS (ESI) m/z: $[M+H]^+$ Calcd for $C_{16}H_{11}{}^{79}BrF_2N$, 334.0037; Found 334.0035. The enantiomeric ratio was determined by HPLC analysis on Daicel Chiralpak OD-3 column. *n*-Hexane/*i*-PrOH = 98:2, flow rate = 1.0 mL/min., λ = 254 nm, t_R = 20.9 min. (minor), 24.7 min. (major).

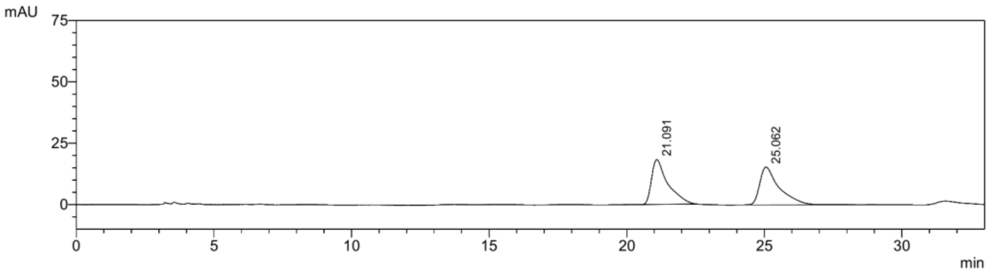

PDA Ch1 240nm

Peak#	Ret. Time	Height	Area	Area%
1	21.091	18274	751440	49.620
2	25.062	15377	762946	50.380
总计		33651	1514386	100.000

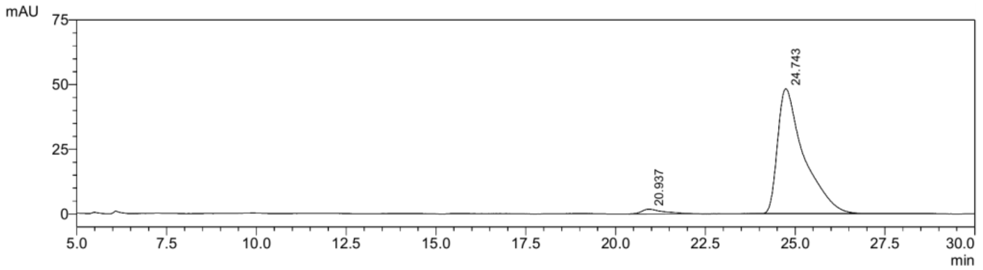

PDA Ch1 250nm

Peak#	Ret. Time	Height	Area	Area%
1	20.937	1797	75372	2.965
2	24.743	48307	2466281	97.035
总计		50104	2541653	100.000

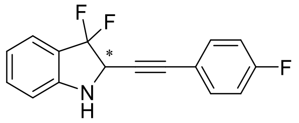

3,3-Difluoro-2-((4-fluorophenyl)ethynyl)indoline (2i): Yellow solid, mp 62-64 ℃, 96% yield. 90% ee. $[\alpha]_D^{25}$ = -62.067 (*c* = 0.5, $CHCl_3$)**.** **^{1}H NMR** (600 MHz, $CDCl_3$) δ 7.49 – 7.48 (m, 1H), 7.45 – 7.43 (m, 2H), 7.35 – 7.33 (m, 1H), 7.01 – 6.98 (m 2H), 6.95 – 6.92 (m, 1H), 6.79 (d, *J* = 8.4 Hz, 1H), 4.89 – 4.85 (m, 1H), 4.27 (s, 1H). **^{13}C NMR** (151 MHz, $CDCl_3$) δ 162.8 (d, $J_{C\text{-}F}$ = 248.9 Hz), 149.9 (t, $J_{C\text{-}F}$ = 6.4 Hz), 133.9 (d, $J_{C\text{-}F}$ = 8.6 Hz), 133.0, 125.0 (t, $J_{C\text{-}F}$ =250.6 Hz), 124.1, 121.0 (t, $J_{C\text{-}F}$ = 25.4 Hz), 120.5, 118.1 (d, $J_{C\text{-}F}$ = 3.8 Hz), 115.6 (d, $J_{C\text{-}F}$ = 22.1 Hz), 112.1, 86.4, 81.2 (t, $J_{C\text{-}F}$ = 6.9 Hz), 57.6 (dd, $J_{C\text{-}F}$ = 36.0, 9.3 Hz). **^{19}F NMR** (565 MHz, $CDCl_3$) δ -87.92 (d, *J* = 246.9 Hz), -89.72 (d, *J* = 248.6 Hz), -109.96. HRMS (ESI) m/z: $[M+H]^+$ Calcd for $C_{16}H_{11}F_3N$, 274.0838; Found 274.0841. The enantiomeric ratio was determined by HPLC analysis on Daicel Chiralpak OD-3 column. *n*-Hexane/*i*-PrOH = 99:1, flow rate = 1.0 mL/min., λ = 254 nm, t_R = 26.6 min. (minor), 30.0 min. (major).

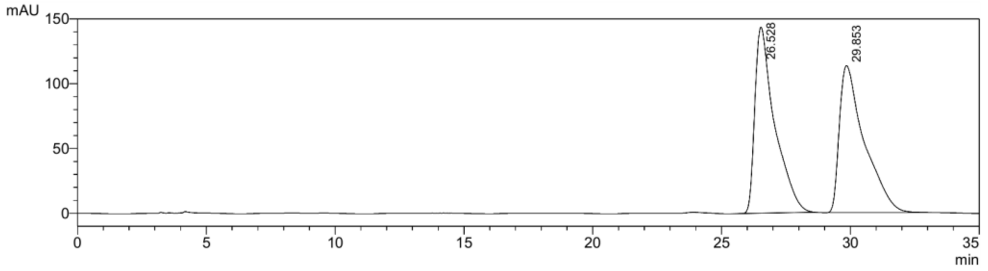

PDA Ch1 240nm

Peak#	Ret. Time	Height	Area	Area%
1	26.528	143665	7699813	50.257
2	29.853	113419	7621129	49.743
总计		257084	15320942	100.000

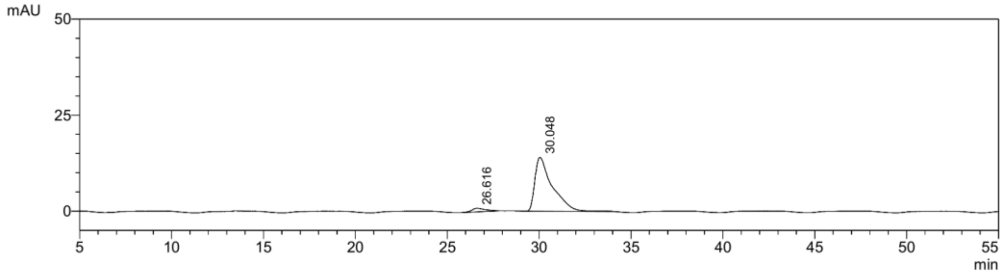

PDA Ch1 254nm

Peak#	Ret. Time	Height	Area	Area%
1	26.616	957	47460	4.846
2	30.048	14052	931827	95.154
总计		15010	979287	100.000

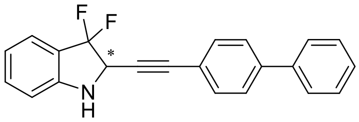

2-([1,1'-Biphenyl]-4-ylethynyl)-3,3-difluoroindoline (2j): Yellow solid, mp 118-119 ℃, 95% yield, 94% ee. $[\alpha]_D^{25}$ = -69.267 (c = 0.5, $CHCl_3$)**.** ^{1}**H NMR** (600 MHz, $CDCl_3$) δ 7.57 – 7.49 (m, 7H), 7.44 – 7.41 (m, 2H), 7.36 – 7.34 (m, 2H), 6.94 – 6.92 (m, 1H), 6.79 (d, J =7.8 Hz, 1H), 4.93 – 4.88 (m, 1H), 4.27 (s, 1H). ^{13}**C NMR** (151 MHz, $CDCl_3$) δ 150.0 (t, $J_{C\text{-}F}$ = 6.3 Hz), 141.5, 140.1, 133.0, 132.3, 128.8, 127.7, 127.0, 126.9, 125.0 (t, $J_{C\text{-}F}$ = 250.4 Hz), 124.1, 121.1 (t, $J_{C\text{-}F}$ = 25.2 Hz), 120.8, 120.4, 112.1, 87.3, 82.1 (t, $J_{C\text{-}F}$ = 7.4 Hz), 57.7 (dd, $J_{C\text{-}F}$ = 36.0, 11.0 Hz). ^{19}**F NMR** (565 MHz, $CDCl_3$) δ -87.76 (d, J = 246.9 Hz), -89.76 (d, J = 247.5 Hz). HRMS (ESI) m/z: $[M+H]^+$ Calcd for $C_{22}H_{16}F_2N$,332.1245; Found 332.1235. The enantiomeric ratio was determined by HPLC analysis on Daicel Chiralpak OD-3 column. n-Hexane/i-PrOH = 98:2, flow rate = 1.0 mL/min., λ = 254 nm, t_R = 32.6 min. (minor), 41.0 min. (major).

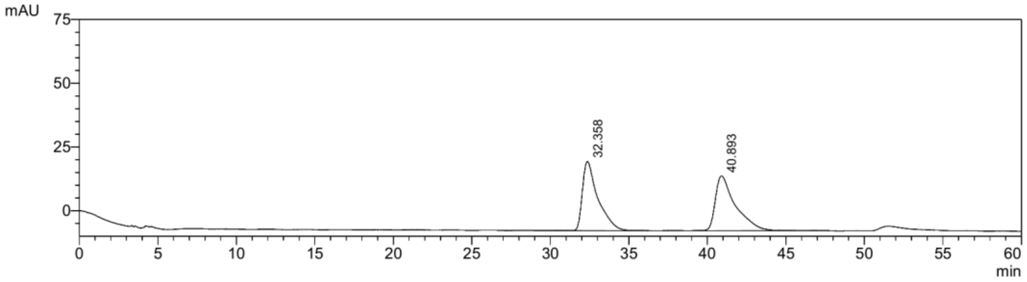

PDA Ch1 274nm

Peak#	Ret. Time	Height	Area	Area%
1	32.358	27116	1873843	49.402
2	40.893	21516	1919202	50.598
总计		48631	3793046	100.000

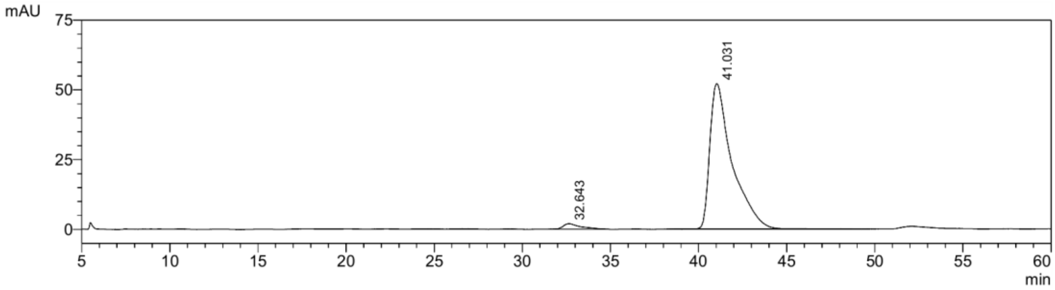

PDA Ch1 273nm

Peak#	Ret. Time	Height	Area	Area%
1	32.643	1951	136960	2.896
2	41.031	52110	4592193	97.104
总计		54061	4729153	100.000

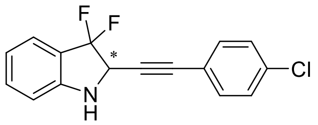

2-((4-Chlorophenyl)ethynyl)-3,3-difluoroindoline (2k): Yellow solid, mp 63-65 ℃, 95% yield, 96% ee. $[\alpha]_D^{25}$ = -76.067 (c = 0.5, $CHCl_3$)**.** ^{1}**H NMR** (600 MHz, $CDCl_3$) δ 7.48 (d, J = 7.8 Hz, 1H), 7.39 – 7.36 (m, 2H), 7.35 – 7.32 (m, 1H), 7.28 – 7.26 (m, 2H), 6.94 – 6.92 (m, 1H), 6.78 (d, J =8.4 Hz,1H), 4.89 – 4.84 (m, 1H), 4.27 (s, 1H). ^{13}**C NMR** (151 MHz, $CDCl_3$) δ 149.9 (t, $J_{C\text{-}F}$ = 6.6 Hz),134.9, 133.1, 133.0, 128.6, 124.9 (t, $J_{C\text{-}F}$ = 250.4 Hz),124.1, 120.9 (t, $J_{C\text{-}F}$ = 25.2 Hz), 120.5, 120.4, 112.1, 86.3, 82.5, 57.6 (dd, $J_{C\text{-}F}$ = 35.9, 10.5 Hz). ^{19}**F NMR** (565 MHz, $CDCl_3$) δ -87.74 (d, J = 246.9 Hz), -89.64 (d, J = 246.9 Hz). HRMS (ESI) m/z: $[M+H]^+$ Calcd for $C_{16}H_{11}{}^{35}ClF_2N$, 290.0543; Found 290.0548. The enantiomeric ratio was determined by HPLC analysis on Daicel Chiralpak OD-3 column. n-Hexane/i-PrOH = 98:2, flow rate = 1.0 mL/min., λ = 254 nm, t_R = 19.7 min. (minor), 22.7 min. (major).

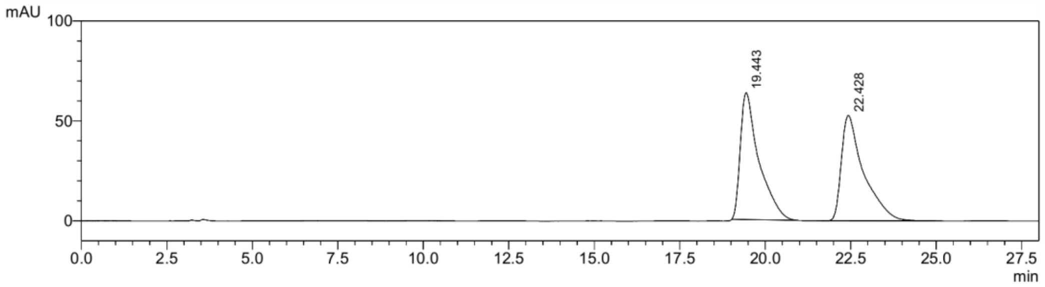

PDA Ch1 247nm

Peak#	Ret. Time	Height	Area	Area%
1	19.443	63541	2406906	50.221
2	22.428	52720	2385682	49.779
总计		116262	4792588	100.000

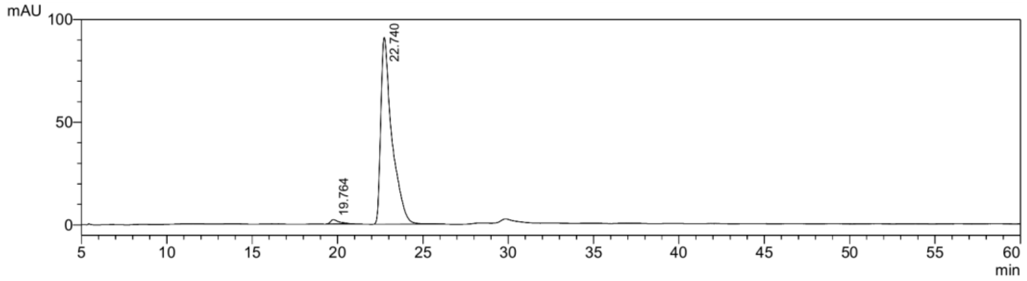

PDA Ch1 247nm

Peak#	Ret. Time	Height	Area	Area%
1	19.764	2121	77993	1.844
2	22.740	91002	4150494	98.156
总计		93123	4228487	100.000

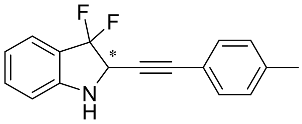

3,3-Difluoro-2-(*p*-tolylethynyl)indoline (2l): Yellow solid, mp 95-97 ℃, 99% yield, 96% ee. $[\alpha]_D^{25}$ = -69.867 (c = 0.5, $CHCl_3$). **^{1}H NMR** (600 MHz, $CDCl_3$) δ 7.48 (d, J = 7.2 Hz, 1H), 7.36 – 7.31 (m, 3H), 7.10 (d, J = 7.2 Hz, 2H), 6.93 – 6.91 (m, 1H), 6.78 (d, J = 7.8 Hz, 1H), 4.89 – 4.85 (m, 1H), 2,33 (s, 3H). **^{13}C NMR** (151 MHz, $CDCl_3$) δ 150.0 (t, $J_{C\text{-}F}$ = 6.5 Hz), 139.0, 132.9, 131.8, 129.0, 125.1 (t, $J_{C\text{-}F}$ = 250.0 Hz), 124.1, 121.1 (t, $J_{C\text{-}F}$ = 25.2 Hz), 120.4, 118.9, 112.1, 87.6, 80.8 (t, $J_{C\text{-}F}$ = 6.8 Hz), 57.7 (dd, $J_{C\text{-}F}$ = 36.2, 11.0 Hz), 21.5. ^{19}F NMR (565 MHz, $CDCl_3$) δ -87.93 (d, J = 246.9 Hz), -89.86 (d, J = 246.9 Hz). HRMS (ESI) m/z: $[M+H]^+$ Calcd for $C_{17}H_{14}F_2N$, 270.1089; Found 270.1096. The enantiomeric ratio was determined by HPLC analysis on Daicel Chiralpak OD-3 column. *n*-Hexane/*i*-PrOH = 99:1, flow rate = 1.0 mL/min., λ = 254 nm, t_R = 40.9 min. (minor), 45.0 min. (major).

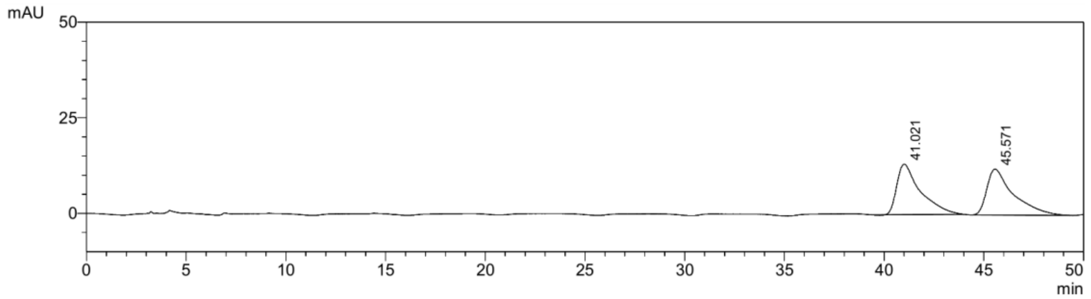

PDA Ch1 244nm

Peak#	Ret. Time	Height	Area	Area%
1	41.021	13215	1091324	49.406
2	45.571	12021	1117546	50.594
总计		25235	2208870	100.000

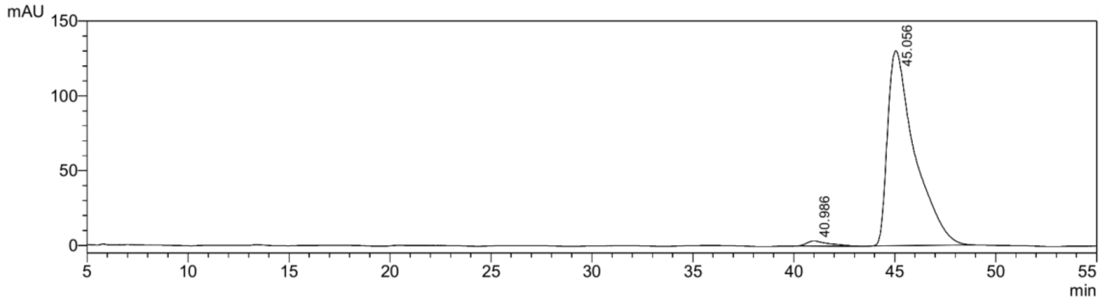

PDA Ch1 244nm

Peak#	Ret. Time	Height	Area	Area%
1	40.986	3397	261972	2.100
2	45.056	130319	12213048	97.900
总计		133716	12475020	100.000

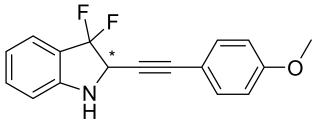

3,3-Difluoro-2-((4-methoxyphenyl)ethynyl)indoline (2m): Yellow solid, mp 96-98 ℃, 99% yield, 93% ee. $[\alpha]_D^{25}$ = -44.133 (*c* = 0.5, $CHCl_3$)**.** **^{1}H NMR** (600 MHz, $CDCl_3$) δ 7.47 (d, *J* = 8.4 Hz, 1H), 7.39 (d, *J* = 8.4 Hz, 2H),7.35 (t, *J* = 7.2 Hz, 2H),6.91 (t, *J* = 7.2 Hz,1H), 6.82 - 6.81 (m, 2H), 6.77 (d, *J* = 8.4 Hz, 1H), 4.88 – 4.84 (m,1H), 4.27 (s, 1H), 3.77 (s, 3H). **^{13}C NMR** (151 MHz, $CDCl_3$) δ 159.9, 150.0 (t, $J_{C\text{-}F}$ = 6.5 Hz), 133.4, 132.9, 125.0 (t, $J_{C\text{-}F}$ = 250.4 Hz), 124.1, 121.0 (t, $J_{C\text{-}F}$ = 25.2 Hz), 120.3, 113.9, 114.0, 112.0, 87.4, 80.1 (t, $J_{C\text{-}F}$ = 7.1 Hz), 57.7 (dd, $J_{C\text{-}F}$ =35, 10.8 Hz), 55.2. **^{19}F NMR** (565 MHz, $CDCl_3$) δ -88.11 (d, *J* = 246.9 Hz), -89.76 (d, *J* = 246.9 Hz). HRMS (ESI) m/z: $[M+H]^+$ Calcd for $C_{17}H_{14}F_2NO$,286.1038; Found 286.1039. The enantiomeric ratio was determined by HPLC analysis on Daicel Chiralpak OD-3

column. *n*-Hexane/*i*-PrOH = 95:5, flow rate = 1.0 mL/min., λ = 254 nm, t_R = 27.3 min. (minor), 31.7 min. (major).

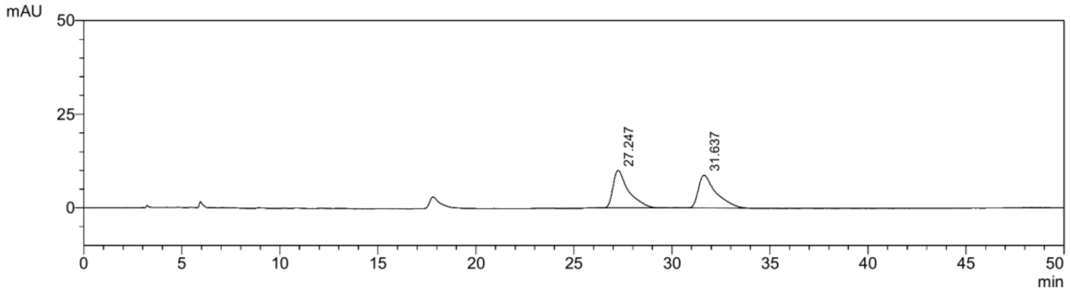

PDA Ch1 254nm

Peak#	Ret. Time	Height	Area	Area%
1	27.247	10013	544702	49.839
2	31.637	8754	548219	50.161
总计		18766	1092921	100.000

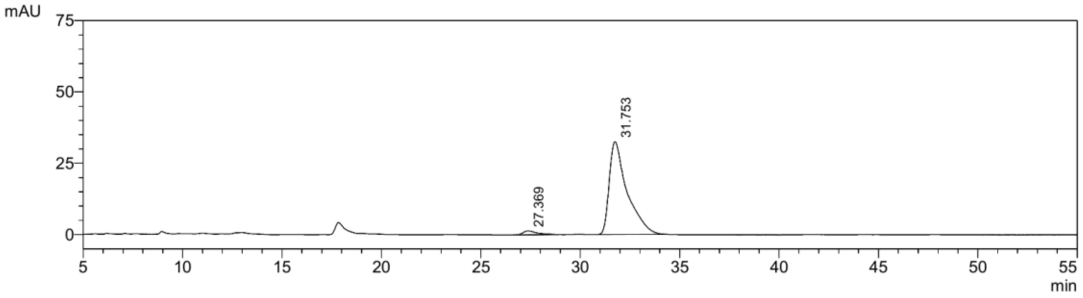

PDA Ch1 254nm

Peak#	Ret. Time	Height	Area	Area%
1	27.369	1381	68461	3.244
2	31.753	32515	2041881	96.756
总计		33896	2110342	100.000

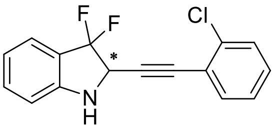

2-((2-Chlorophenyl)ethynyl)-3,3-difluoroindoline (2n): Yellow solid, mp 54-54 ℃, 95% yield, 94% ee. $[\alpha]_D^{25}$ = -57.067 (*c* = 0.5, $CHCl_3$). **^{1}H NMR** (600 MHz, $CDCl_3$) δ 7.50 – 7.47 (m, 2H), 7.38 – 7.32 (m, 2H), 7.26 – 7.23 (m, 1H), 7.20 – 7.17 (m, 1H), 6.94 – 6.92 (m, 1H), 6.80 (d, *J* = 6.4 Hz, 1H), 4.96 – 4.91 (m, 1H), 4.31 (s, 1H). **^{13}C NMR** (151 MHz, CDCl3) δ 149.9 (t, $J_{C\text{-}F}$ = 6.9 Hz), 136.2, 133.7, 133.0, 129.9, 129.3, 126.4, 125.0 (t, $J_{C\text{-}F}$ = 251.3 Hz), 124.1, 122.0, 121.0 (t, $J_{C\text{-}F}$ = 25.7 Hz),120.5, 112.1, 86.8 (t, $J_{C\text{-}F}$ = 7.1 Hz), 84.1, 57.7 (dd., $J_{C\text{-}F}$ = 35.9, 11.1 Hz). **^{19}F NMR** (565 MHz, $CDCl_3$) δ -87.49 (d, *J* = 246.9 Hz), -89.64 (d, *J* = 247.5 Hz). HRMS (ESI) m/z: $[M+H]^+$ Calcd for $C_{16}H_{11}{}^{35}ClF_2N$, 290.0543; Found 290.0547. The enantiomeric ratio was

determined by HPLC analysis on Daicel Chiralpak OD-3 column. *n*-Hexane/*i*-PrOH = 98:2, flow rate = 1.0 mL/min., λ = 254 nm, t_R = 22.1 min. (minor), 25.5 min. (major).

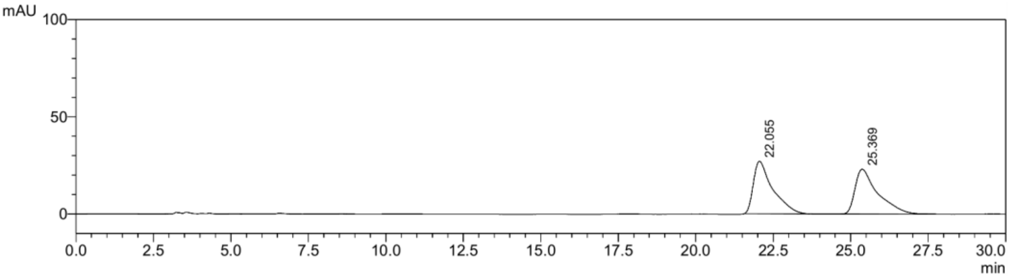

PDA Ch1 240nm

Peak#	Ret. Time	Height	Area	Area%
1	22.055	26969	1169868	49.769
2	25.369	23096	1180738	50.231
总计		50065	2350605	100.000

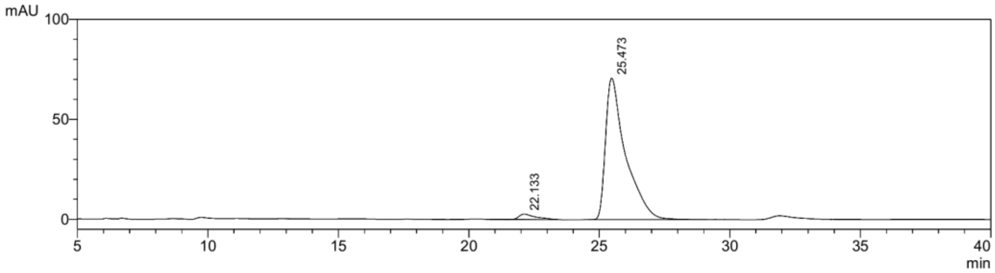

PDA Ch1 243nm

Peak#	Ret. Time	Height	Area	Area%
1	22.133	2708	113707	2.923
2	25.473	70816	3775939	97.077
总计		73524	3889646	100.000

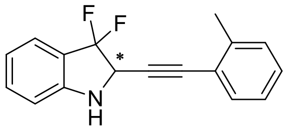

3,3-Difluoro-2-(*o*-tolylethynyl)indoline (2o): Yellow solid, mp 56-57 ℃, 99% yield. 96% ee. $[\alpha]_D^{25}$ = -64.933 (*c* = 0.5, $CHCl_3$)**.** **^{1}H NMR** (600 MHz, $CDCl_3$) δ 7.49 (d, *J*= 7.8 Hz,1H), 7.42 (d, *J* = 7.2 Hz,1H), 7.35 – 7.32 (m, 1H), 7.23 – 7.17 (m, 2H), 7.13 – 7.10 (m, 1H), 6.93 (t, *J* = 7.8 Hz,1H), 6.79 (d, *J* = 7.2 Hz,1H), 4.94 – 4.89 (m,1H), 4.26 (s, 1H), 2.42 (s, 3H). **^{13}C NMR** (151 MHz, $CDCl_3$) δ 150.0 (t, $J_{C\text{-}F}$ = 6.6 Hz), 140.7, 132.9, 132.1, 129.4, 128.8, 125.5, 125.1 (t, $J_{C\text{-}F}$ = 250.5 Hz), 124.1, 121.8, 121.1(t, $J_{C\text{-}F}$ = 25.4 Hz), 120.4, 112.1, 86.3, 85.3 (t, $J_{C\text{-}F}$ = 6.6 Hz), 57.8 (dd, $J_{C\text{-}F}$ = 35.9, 10.7 Hz), 20.5. **^{19}F NMR** (565 MHz, $CDCl_3$) δ -88.27 (d, *J* = 251.4 Hz), -89.74 (d, *J* = 253.1 Hz). HRMS (ESI) m/z: $[M+H]^+$ Calcd for $C_{17}H_{14}F_2N$, 270.1089; Found 270.1097. The enantiomeric ratio was determined by HPLC analysis on Daicel Chiralpak OD-3

column. *n*-Hexane/*i*-PrOH = 98:2, flow rate = 1.0 mL/min., λ = 254 nm, t_R = 20.1 min. (minor), 23.9 min. (major).

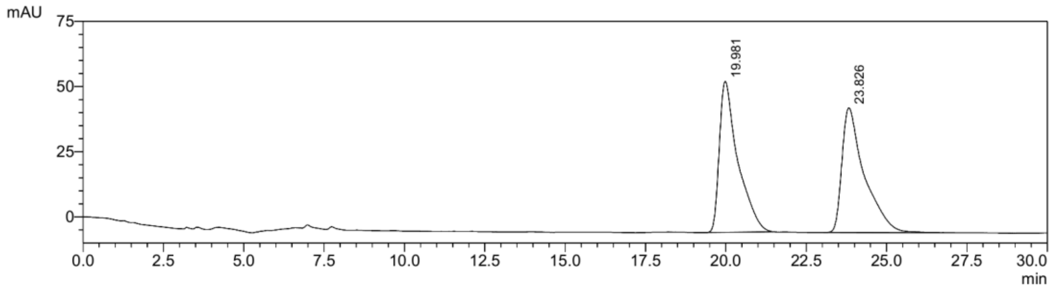

PDA Ch1 242nm

Peak#	Ret. Time	Height	Area	Area%
1	19.981	57915	2317401	50.008
2	23.826	47833	2316656	49.992
总计		105748	4634057	100.000

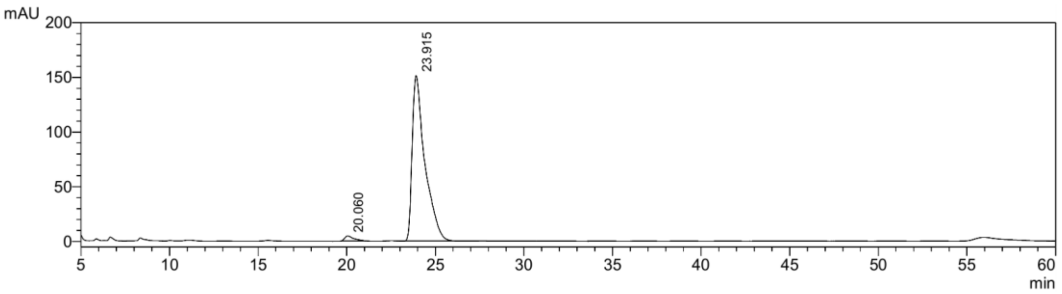

PDA Ch1 242nm

Peak#	Ret. Time	Height	Area	Area%
1	20.060	4480	158436	2.061
2	23.915	151108	7529638	97.939
总计		155588	7688075	100.000

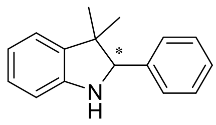

3,3-Dimethyl-2-phenylindoline (2p): Yellow solid, mp 95-97 ℃, 95% yield, 98% ee. (CAS: 1247138-74-4).[7] $[\alpha]_D^{25}$ = 50.467 (*c* = 0.5, $CHCl_3$)**. ^{1}H NMR** (600 MHz, CDCl$_3$) δ 7.40 (d, *J* = 7.2 Hz, 2H),7.30 (t, *J* = 7.8 Hz, 2H), 7.27 – 7.24 (m, 1H), 7.07 – 7.00 (m, 2H), 6.76 – 6.73 (m, 1H), 6.64 – 6.63 (d, *J* = 7.8 Hz, 1H), 4.70 (s, 3H), 4.05 (s, 1H), 1.58 (s, 3H), 0.89 (s, 3H). **^{13}C NMR** (151 MHz, $CDCl_3$) δ 149.2, 139.9, 137.9, 127.9, 127.4, 127.3, 127.2, 122.3, 118.8, 109.0, 74.4, 45.2, 26.4, 24.4. The enantiomeric ratio was determined by HPLC analysis on Daicel Chiralpak OD-3 column. *n*-Hexane/*i*-PrOH = 98:2, flow rate = 1.0 mL/min., λ = 254 nm, t_R = 12.5 min. (minor), 35.8 min. (major)

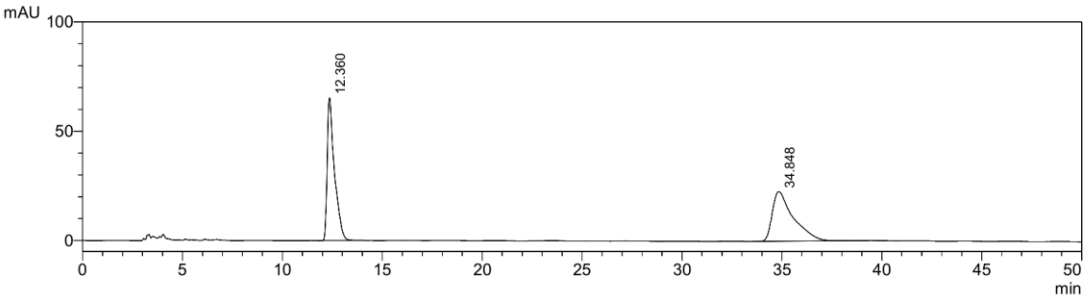

PDA Ch1 239nm

Peak#	Ret. Time	Height	Area	Area%
1	12.360	65289	1657007	49.892
2	34.848	22771	1664151	50.108
总计		88061	3321158	100.000

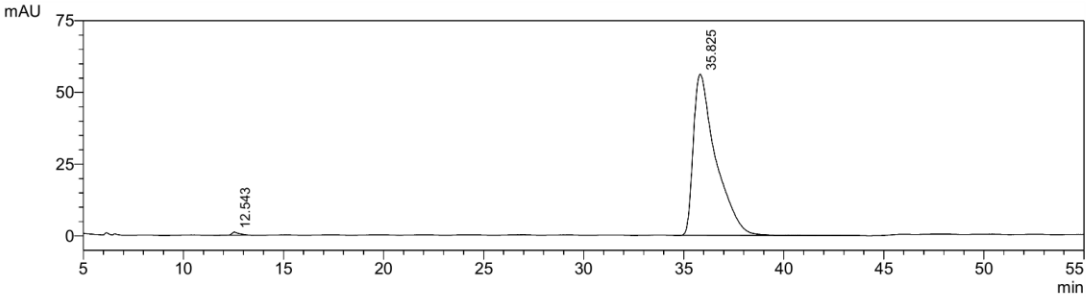

PDA Ch1 238nm

Peak#	Ret. Time	Height	Area	Area%
1	12.543	1092	27994	0.637
2	35.825	56199	4369158	99.363
总计		57291	4397152	100.000

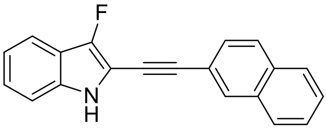

3-Fluoro-2-(naphthalen-2-ylethynyl)-1*H*-indole (2q): Yellow solid. mp 92-94 ℃, 95% yield. **^{1}H NMR** (600 MHz, $CDCl_3$) δ 8.08 (s, 1H), 7.82 (d, J = 7.8 Hz,2H), 7.77 (s, 1H), 7.63 – 7.58 (m, 2H), 7.52 – 7.50 (m, 2H), 7.27 – 7.25 (m, 2H), 7.17 – 7.15 (m, 1H). **^{13}C NMR** (151 MHz, $CDCl_3$) δ 148.0 (d, $J_{C\text{-}F}$ = 252.5 Hz), 133.0, 132.9, 132.7, 128.2, 127.9, 127.9, 127.8, 127.0, 126.8, 124.7, 120.6, 119.7, 117.2, 117.1, 116.6, 116.5, 111.3, 103.4 (d, $J_{C\text{-}F}$ = 23.4 Hz), 97.6. **^{19}F NMR** (565 MHz, $CDCl_3$) δ -163.74. HRMS (ESI) m/z: $[M+H]^+$ Calcd for $C_{20}H_{13}FN$, 286.1027; Found 286.1032.

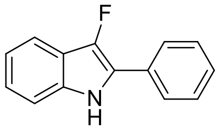

3-Fluoro-2-phenyl-1*H*-indole (2r): Yellow solid. mp 85-87 ℃, 97% yield. (CAS:

1914069-35-4)[8]. **^{1}H NMR** (600 MHz, $CDCl_3$) δ 7.86 (s, 1H), 7.76 (d, *J* = 7.8 Hz, 2H), 7.67 (d, *J* = 7.8 Hz, 1H), 7.50 (t, *J* = 7.8 Hz, 2H), 7.37 – 7.35 (m, 2H), 7.27 – 7.25(m, ,1H), 7.20 – 7.18 (m, ,1H). **^{13}C NMR** (151 MHz, $CDCl_3$) δ 142.4 (d, $J_{C\text{-}F}$ = 246.6 Hz), 132.4 (d, $J_{C\text{-}F}$ = 6.5 Hz), 132.0(d, $J_{C\text{-}F}$ = 5.0 Hz), 129.0, 127.4, 125.3 (d, $J_{C\text{-}F}$ = 5.3 Hz), 123.4, 120.3, 119.5 (d, $J_{C\text{-}F}$ = 18.6 Hz), 118.6 (d, $J_{C\text{-}F}$ = 16.4 Hz), 116.8 (d, $J_{C\text{-}F}$ = 2.9Hz), 111.3. **^{19}F NMR** (565 MHz, $CDCl_3$) δ -170.37.

We have attempted the derivatization of alkyne **2a** via hydrogenation or [3 + 2]-cycloaddition with azidotrimethylsilane. Neither of them succeeded.

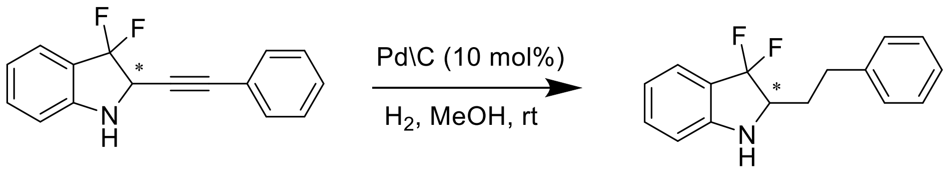

2a

A 100-mL Schlenk flask was charged with compound **2a** (25 mg, 0.1 mmol, 1.0 equiv), Pd/C (5 wt %, 5 mg, 10 mol %), MeOH (2.0 mL). The reaction flask was charged with 1 bar H_2. After reaction for either 8 or 36 hours, there was no target product detected. Only reactant and a series of side products including defluorinated product 3-fluoro-2-(phenylethynyl)-1*H*-indole were observed by ^{19}F NMR of the crude reaction mixture.

^{19}**F NMR** (565 MHz, $CDCl_3$) δ -87.81 (d, J = 247.5 Hz), -89.93 (d, J = 246.9 Hz), -164.03.

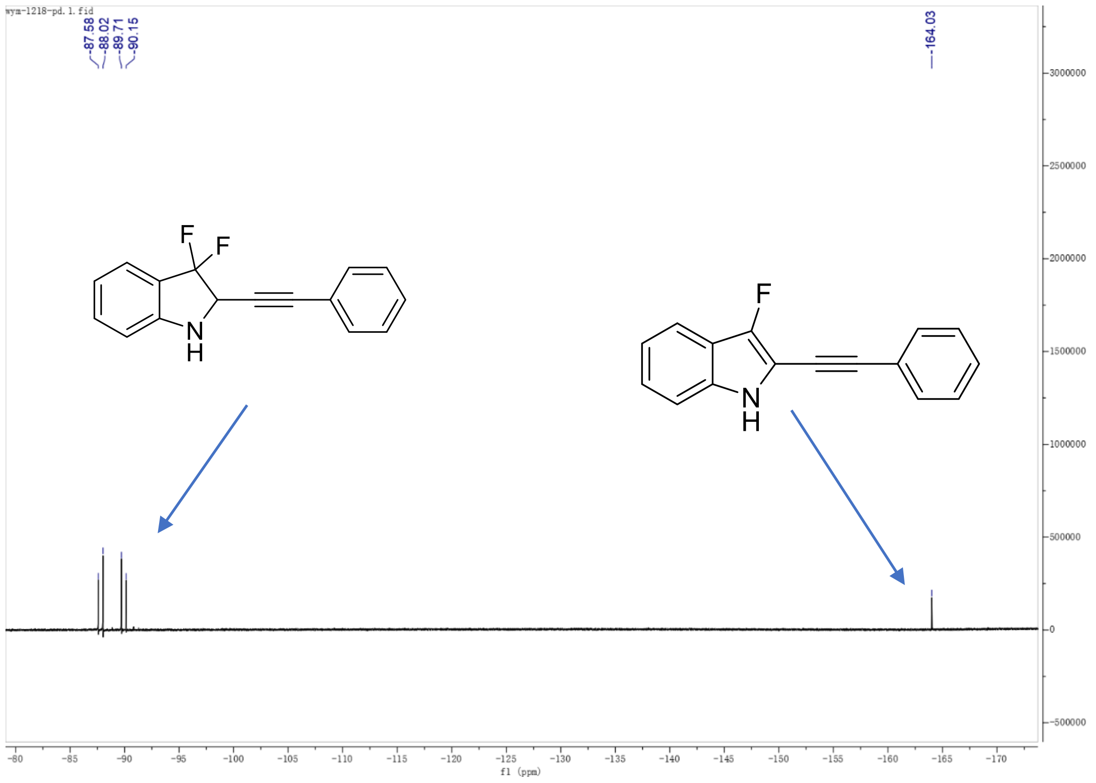

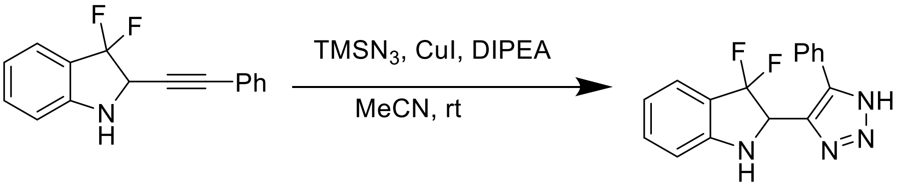

2a

Into a round-bottomed flask were added compound **2a** (25 mg, 0.1 mmol, 1.0 equiv), MeCN (1.6 mL), DIPEA (259 mg, 2.0 mmol, 20.0 equiv), and $TMSN_3$ (35 mg, 0.3 mmol, 3.0 equiv). After being stirred for 5 minutes, CuI (19 mg, 0.1 mmol, 1.0 equiv) was then added into reaction mixture. After reaction for 0.5 h, the reaction mixture was monitored by ^{19}F NMR. There was no target product observed. Only starting material **2a** and a series of side products including defluorinated product 3-fluoro-2-(phenylethynyl)-1*H*-indole were observed on ^{19}F NMR of crude reaction mixture.

^{19}F NMR (565 MHz, $CDCl_3$) δ-87.81 (d, *J* = 247.5 Hz), -89.93 (d, *J* = 246.9 Hz), -122.44, -164.03

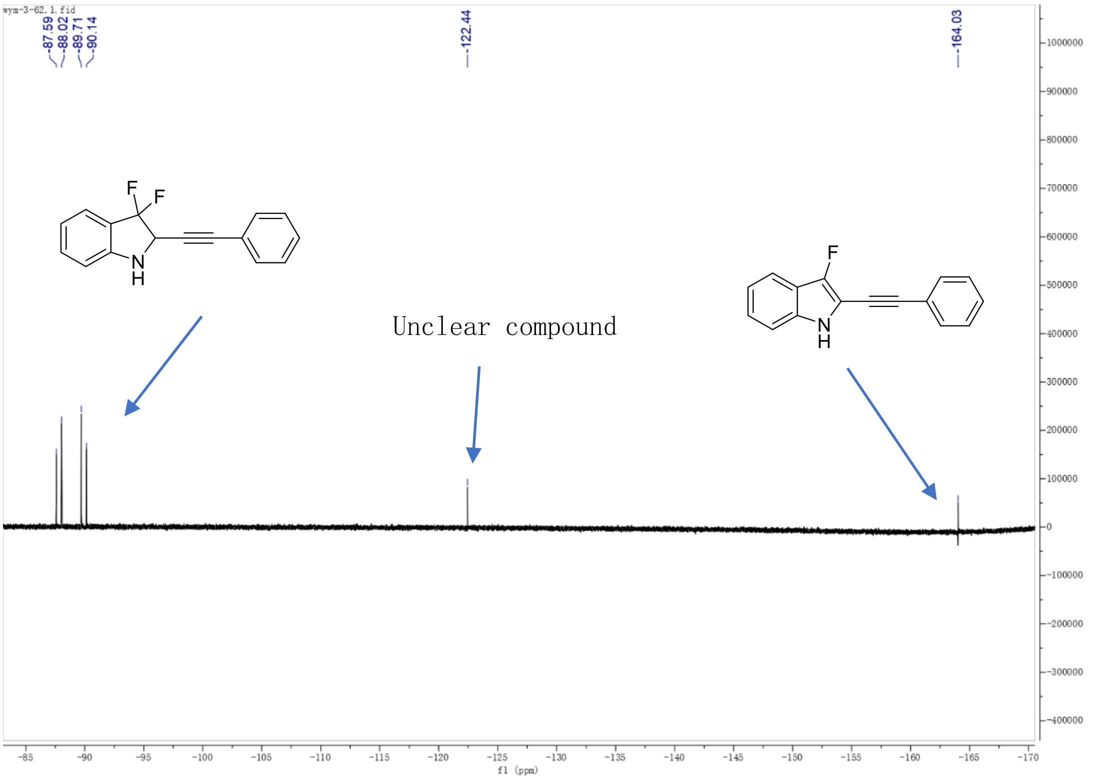

4. References

1. Fang, Y.-Q.; Lautens, M. A. *J. Org. Chem*. **2008**, *73*, 538–549. doi:10.1021/jo701987r.

2. Iioka R, Yorozu K, Sakai Y. *Eur. J. Org. Chem*. **2021**, *10*, 1553-1558. doi: 10.1002/ejoc.202100021.

3. Mao, X.- Y.; Lin, X.-T.; Yang, M.; Chen, G.-S.; Liu, Y.-L. *Adv. Synth. Catal*. **2018**, *360*, 3643-3648. doi:10.1002/adsc.201800716.

4. Chen, Q.-P.; Zhu, Y.-L.; Shi, X.-J.; Huang, R.-F.; Jiang, C.; Zhang, K.;Liu, G.-H.. *Chem Sci*, **2023**, *14*, 1715-1723. doi: /10.1039/d2sc06340a.

5. Luo, J.; Fang, Y.-B,; Mao, X.-Y.; Yang, M.; Zhao, Y.-L.; Liu, Y.-L.;Chen, G.-S.; Ji, Y.-F. *Adv. Synth. Cata*l. **2019**, *361*, 1408–1413. doi: 10.1002/adsc.201801524.

6. Lin, R.-Y.; Ding, S.-T.; Shi, Z.-S.; Jiao, N. *Org. Lett.* **2011**, *13*, 4498–4501. doi: 10.1021/ol201896p.

7. Borrmann R, Knop N, Rueping M. *Chem.A Eur. J*. **2017**, 798-801. doi: 10.1002/chem.201605450.

8. Sancheti, S.-P.; Mondal, D.-J.; Patil, N.-T. *ACS Catal*. **2023**, *13*, 4391-4397. doi: 10.1021/acscatal.3c00088.

5. NMR spectra copies

^{1}H NMR spectra of **1a**

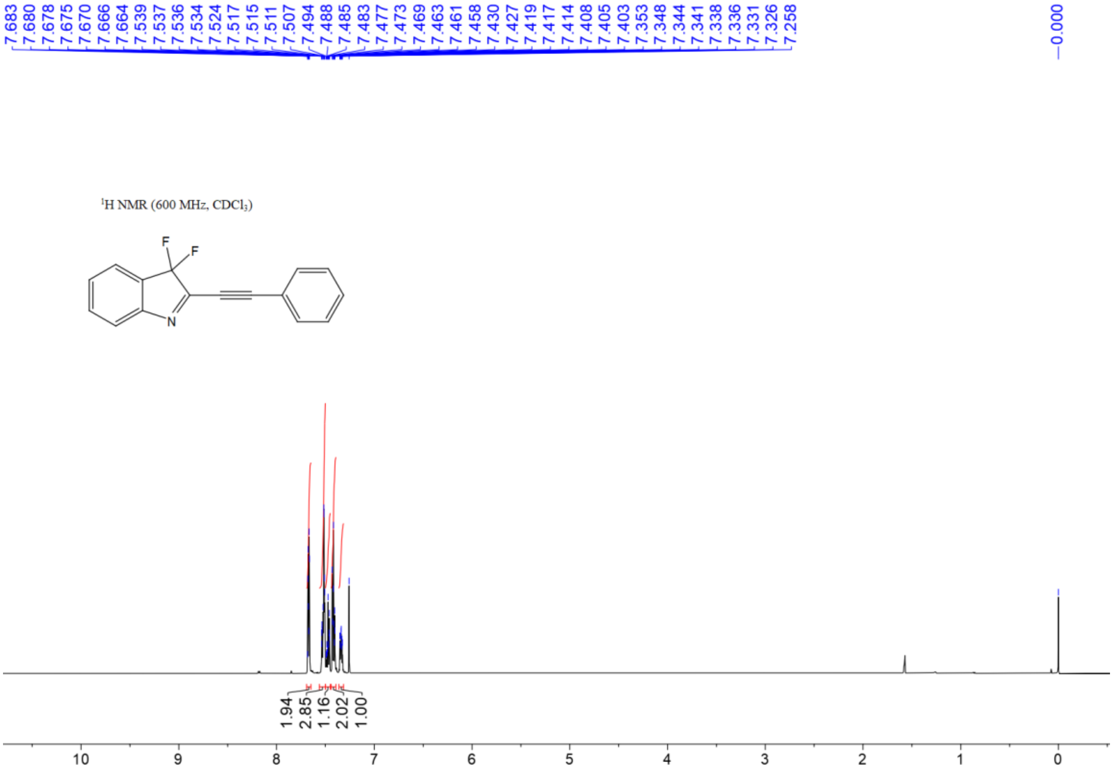

^{13}C NMR spectra of **1a**

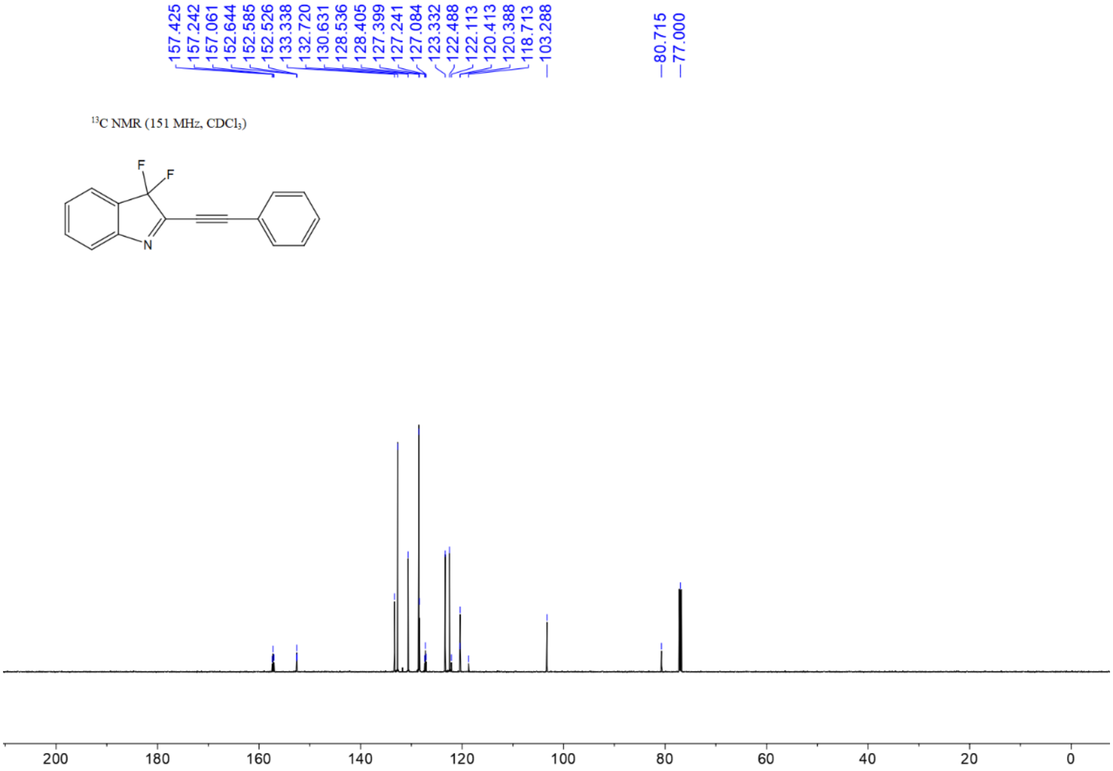

^{19}F NMR spectra of **1a**

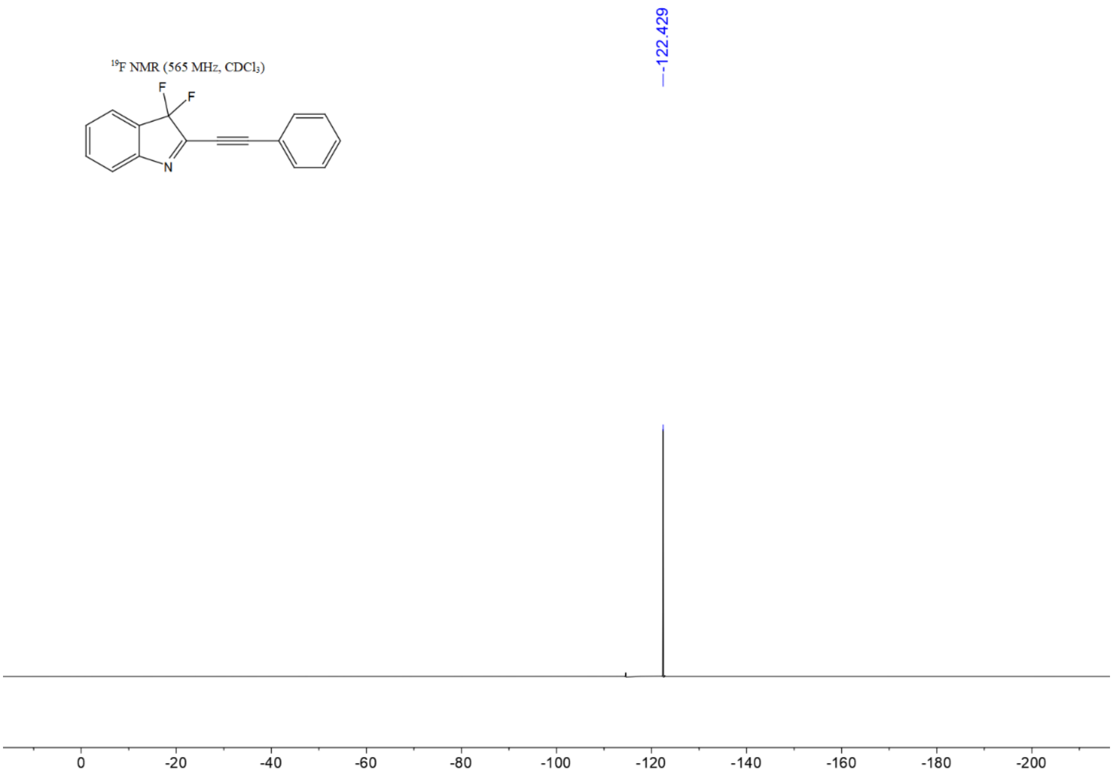

^{1}H NMR spectra of **1b**

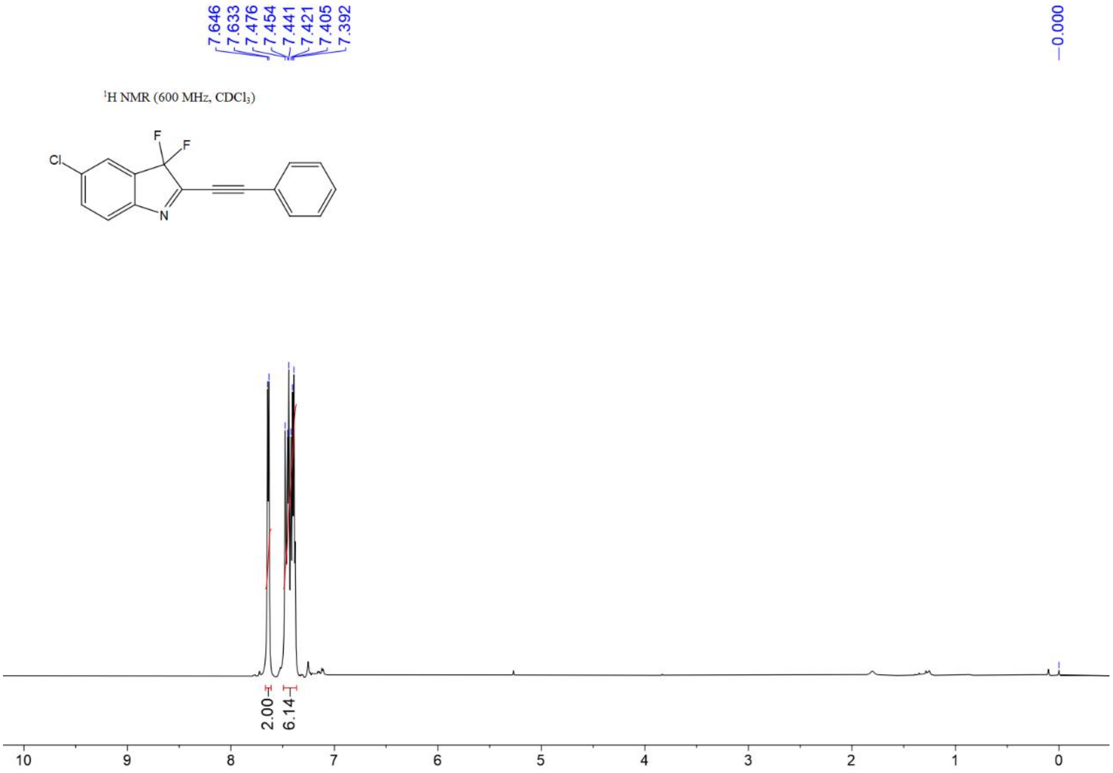

^{13}C NMR spectra of **1b**

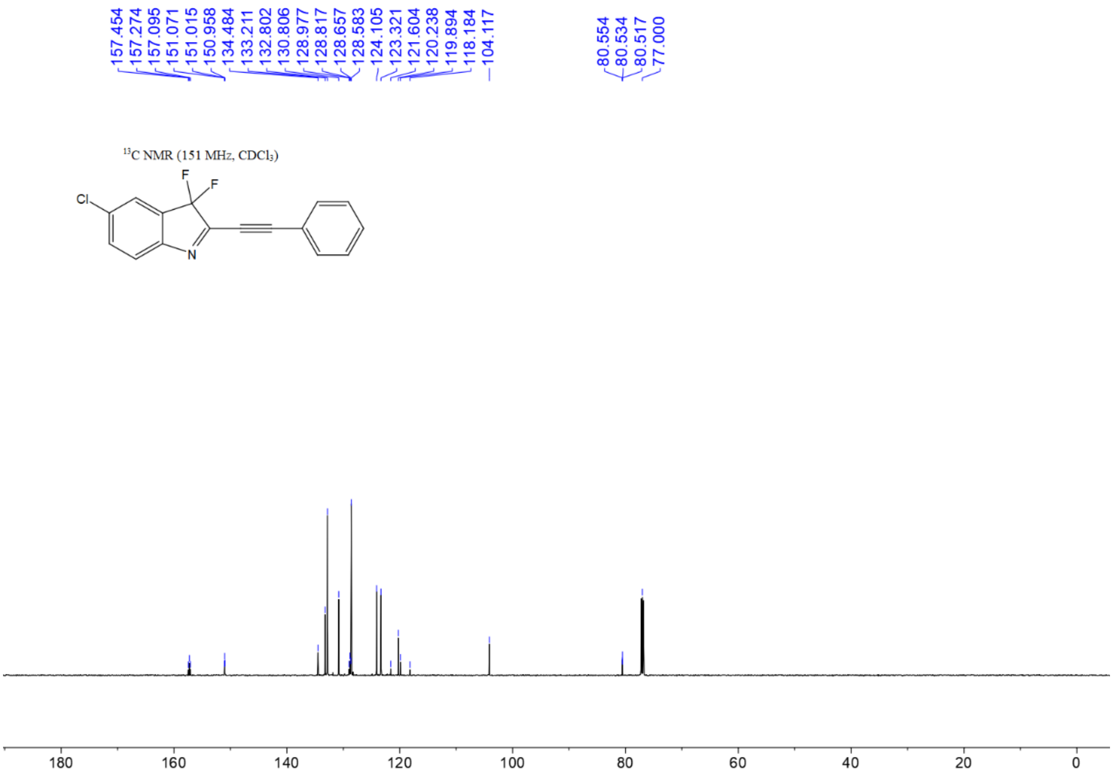

^{19}F NMR spectra of **1b**

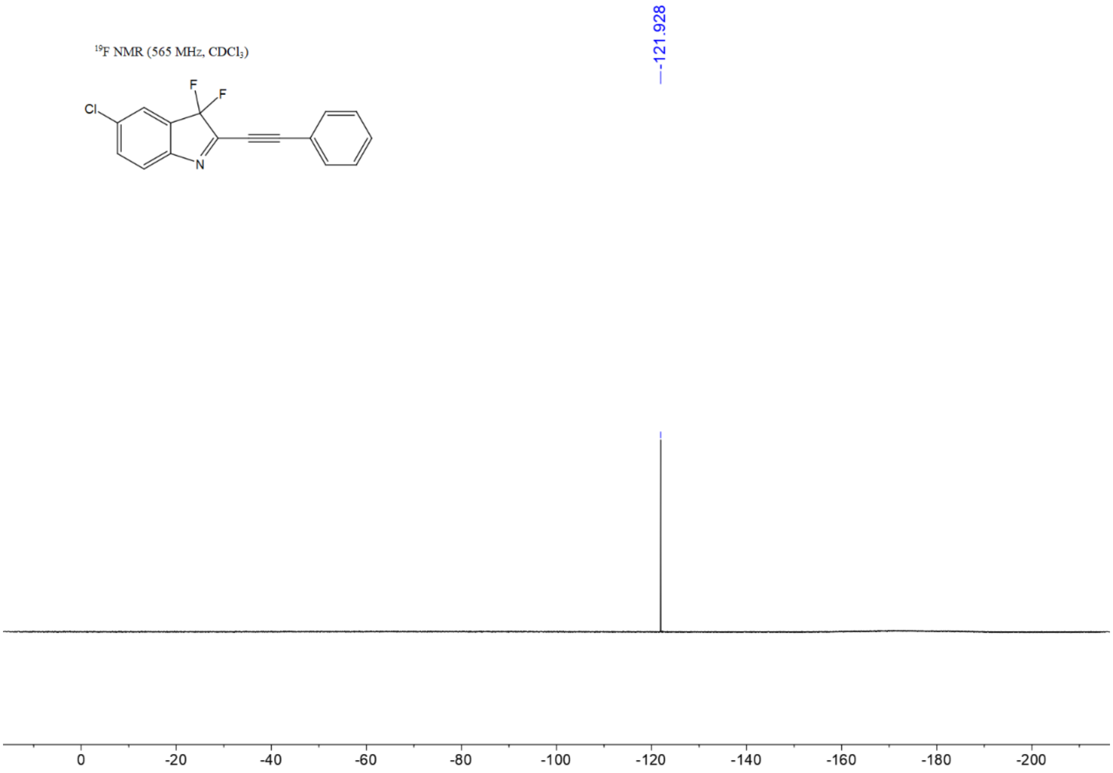

^{1}H NMR spectra of **1c**

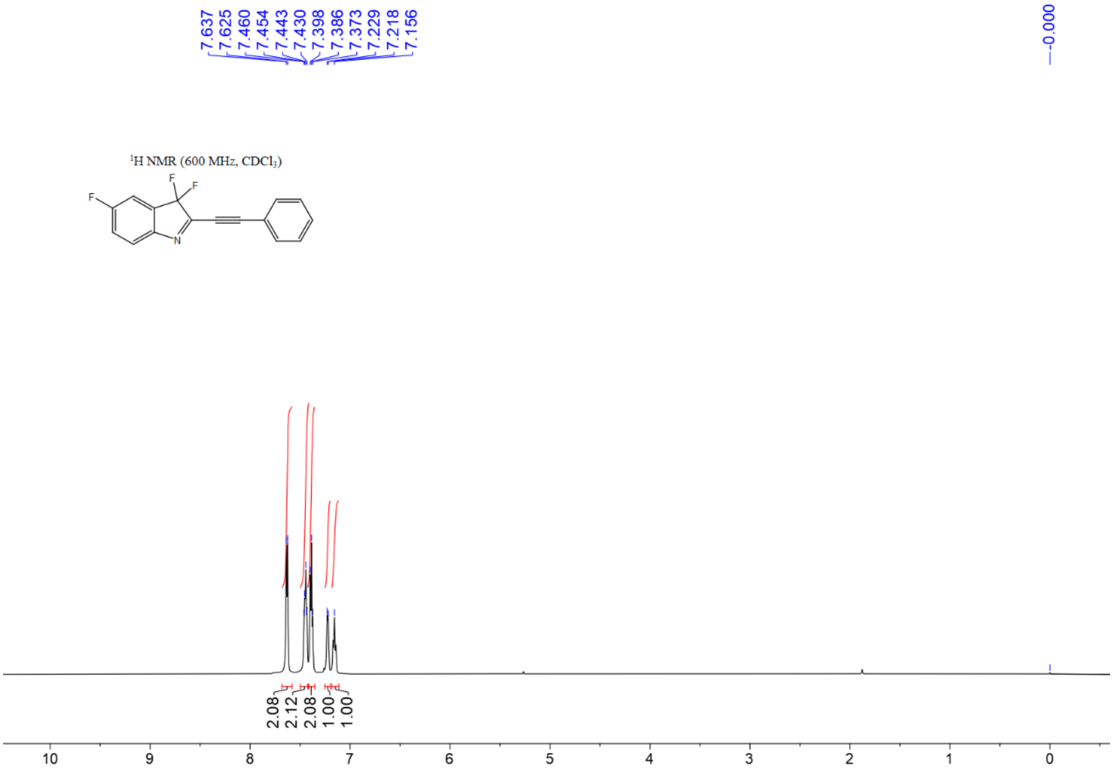

^{13}C NMR spectra of **1c**

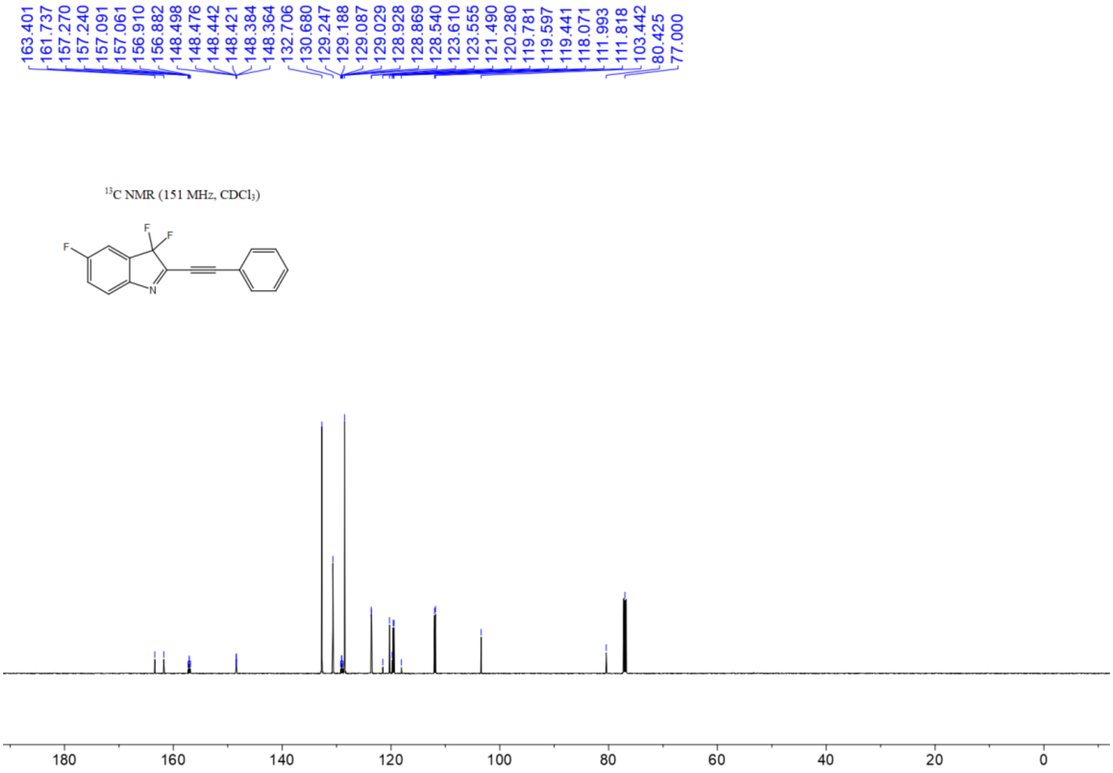

^{19}F NMR spectra of **1c**

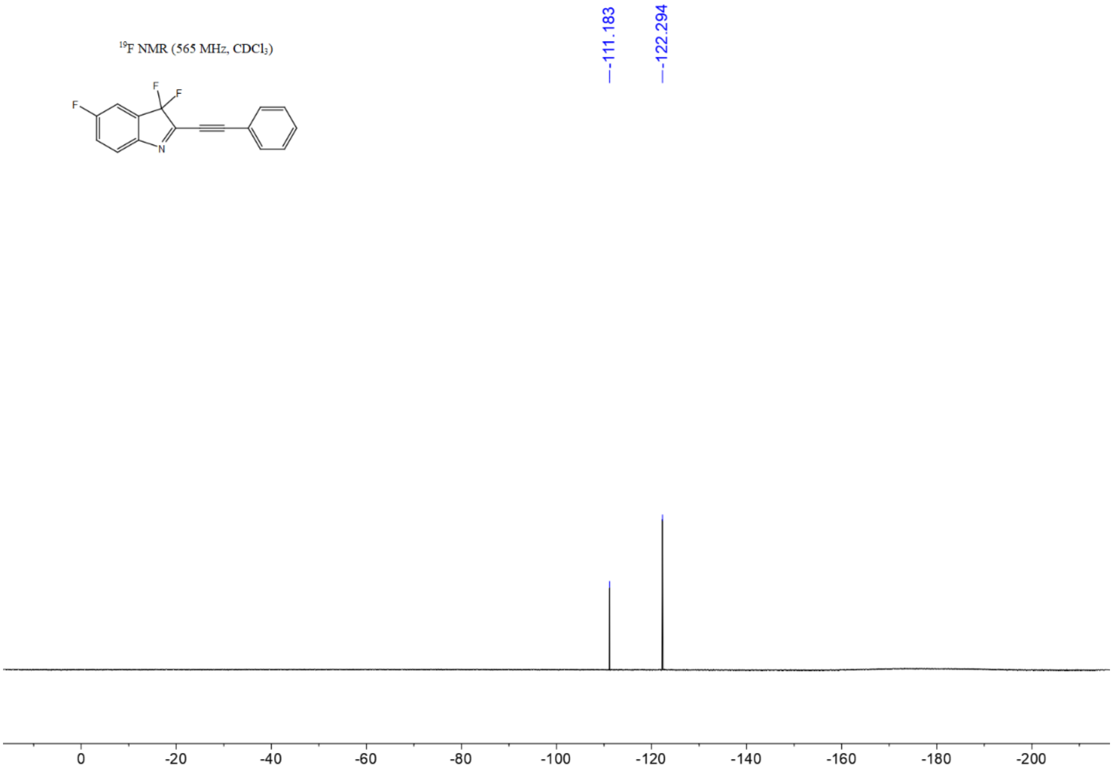

^{1}H NMR spectra of **1d**

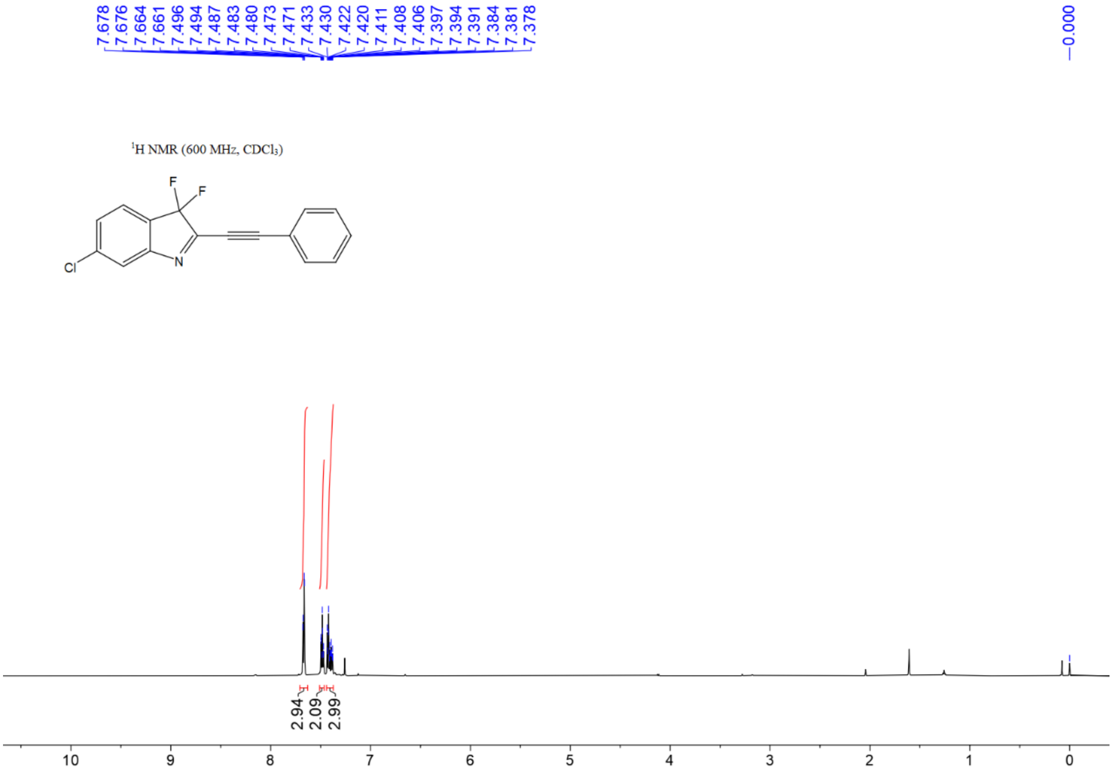

^{13}C NMR spectra of **1d**

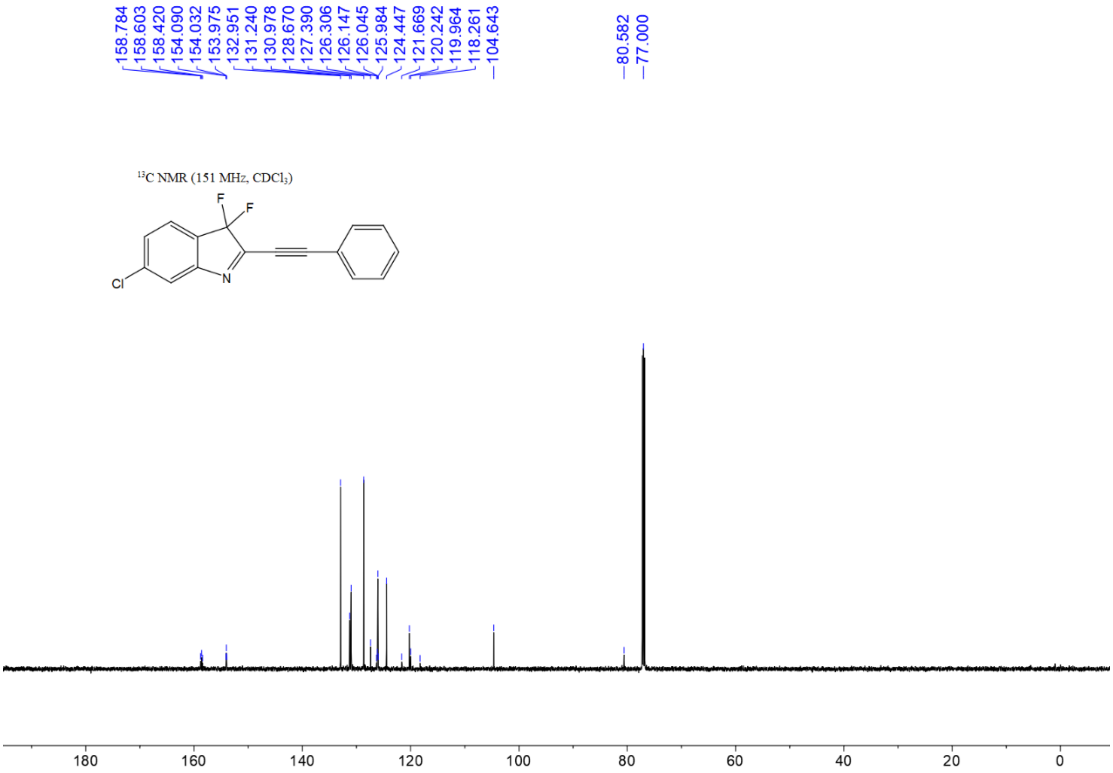

^{19}F NMR spectra of **1d**

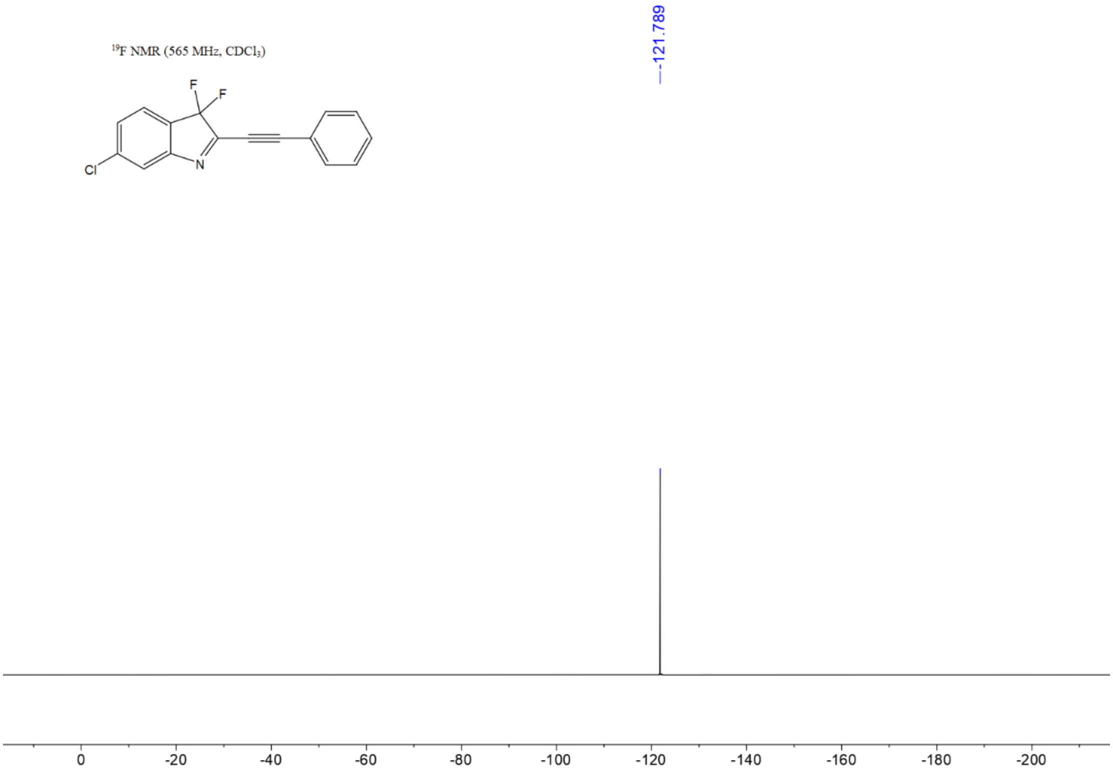

^{1}H NMR spectra of **1e**

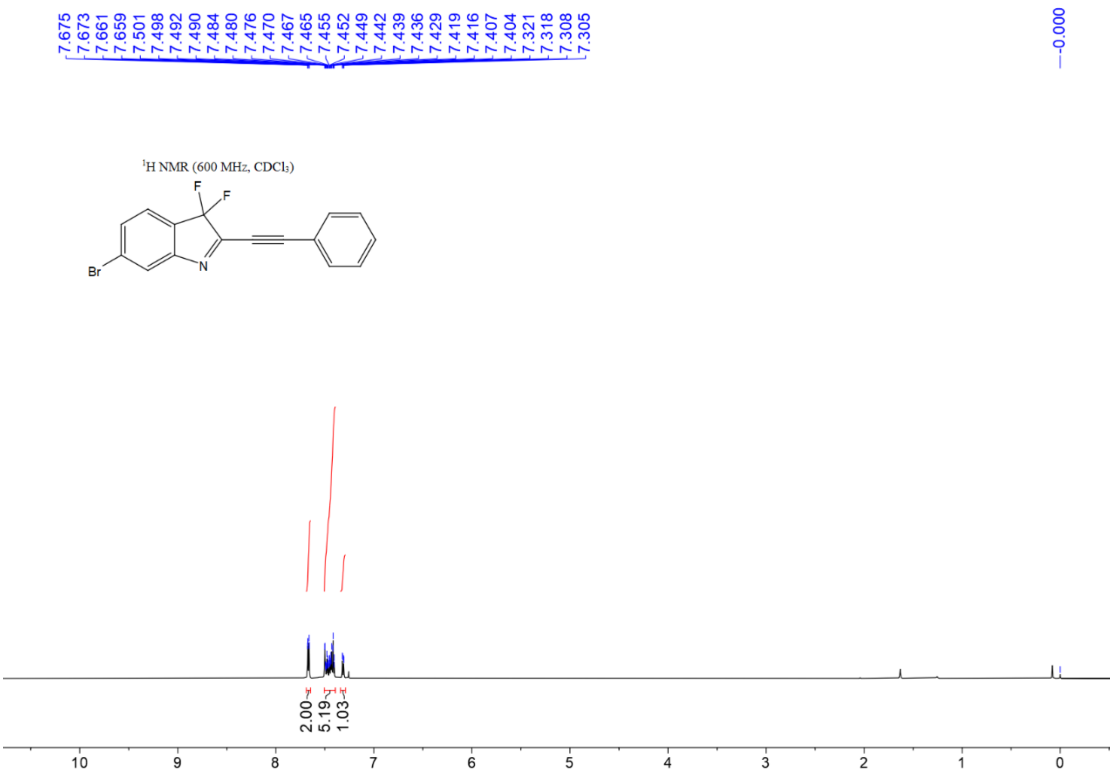

^{13}C NMR spectra of **1e**

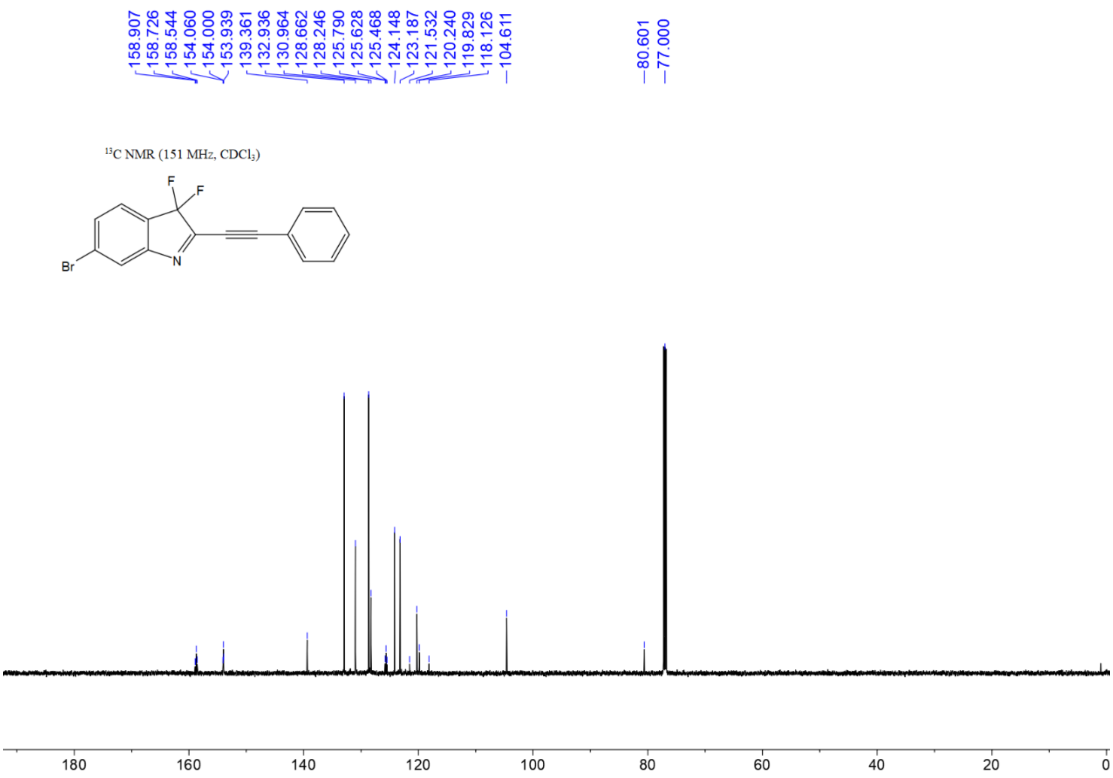

^{19}F NMR spectra of **1e**

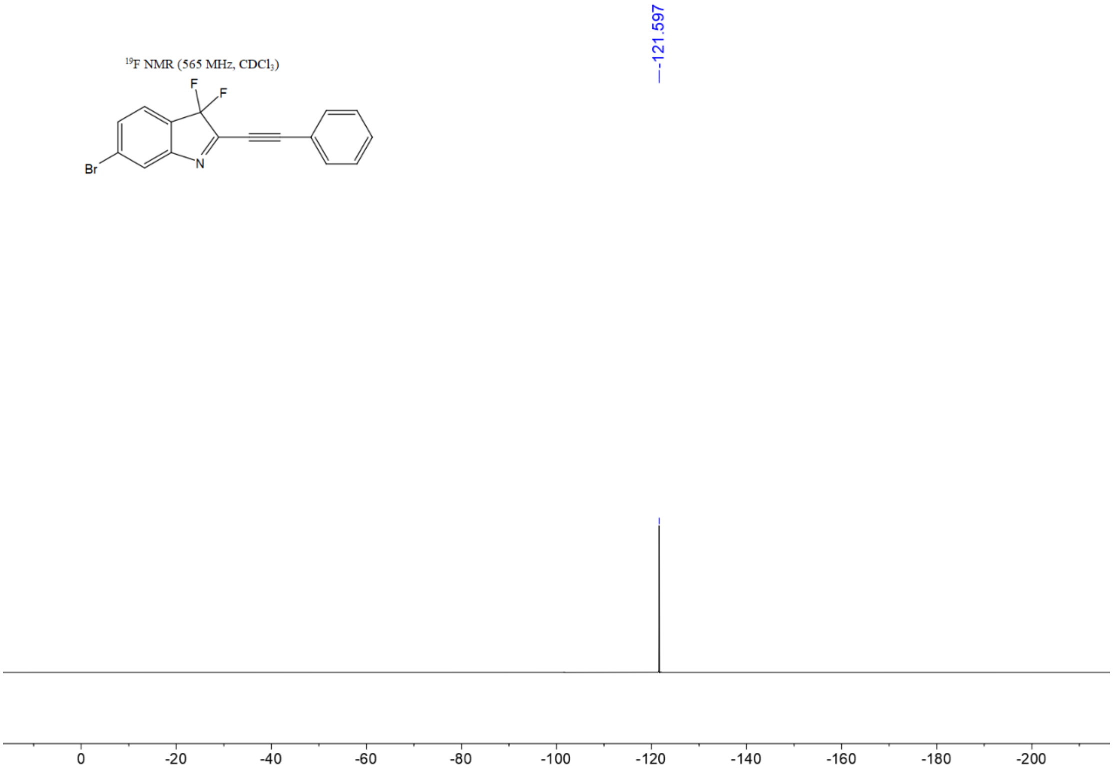

^{1}H NMR spectra of **1f**

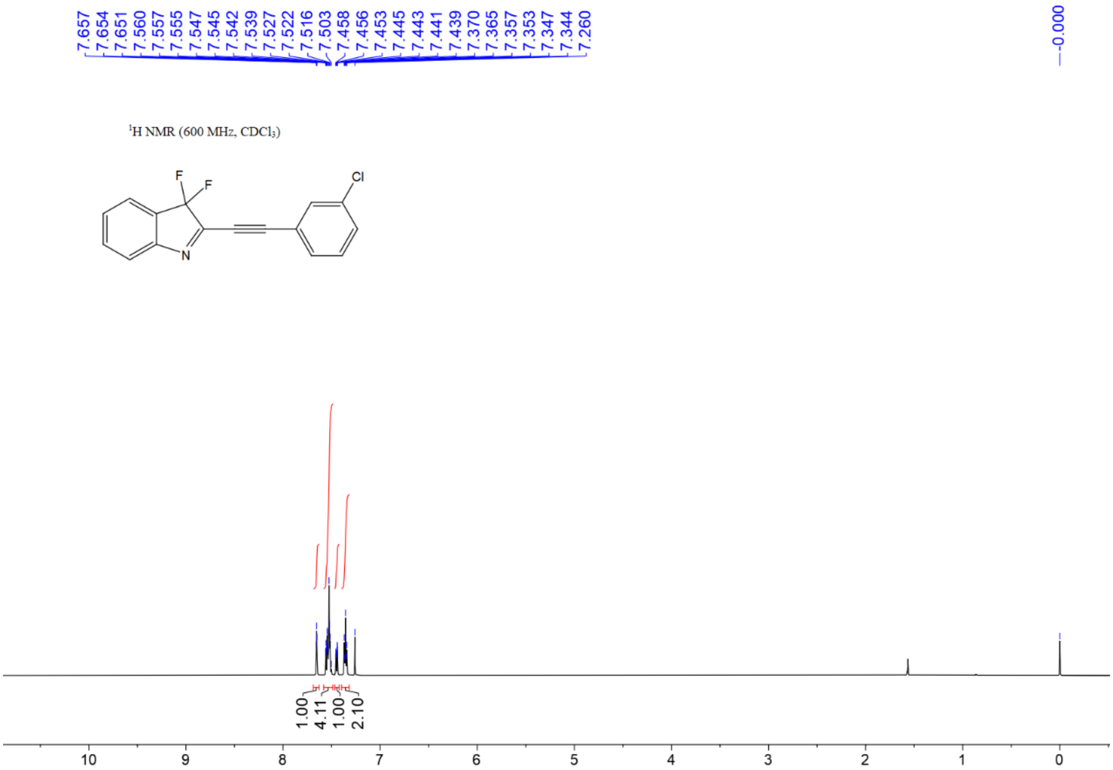

^{13}C NMR spectra of **1f**

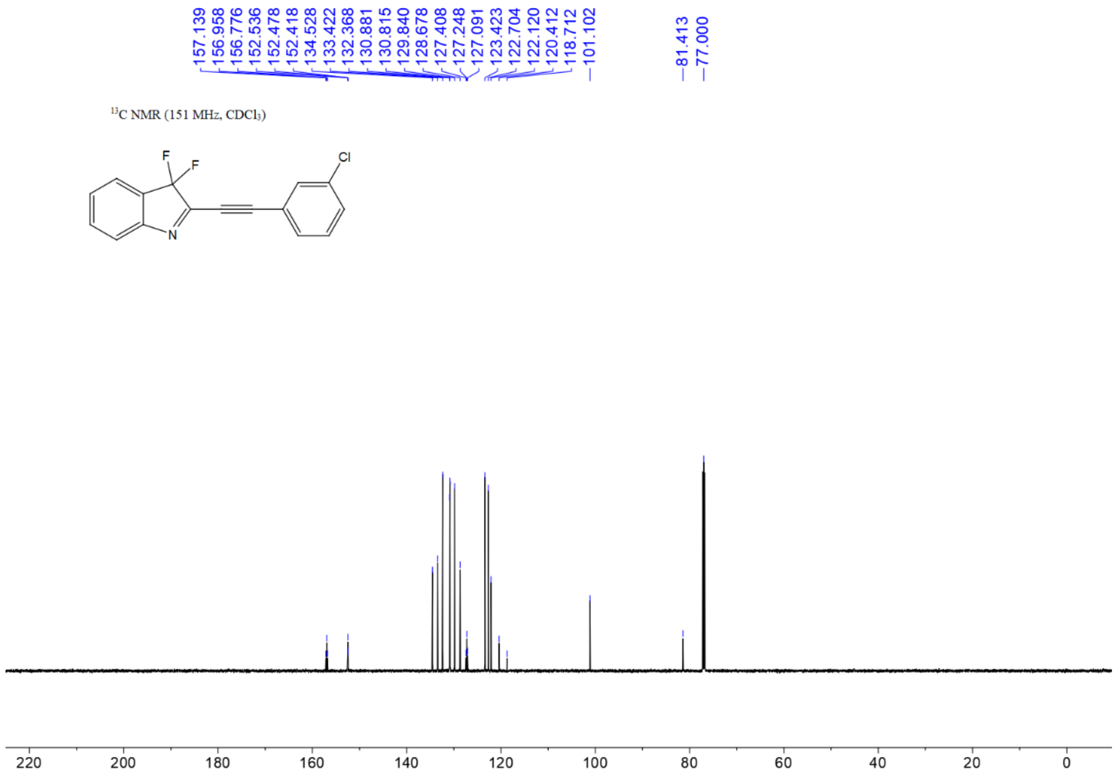

^{19}F NMR spectra of **1f**

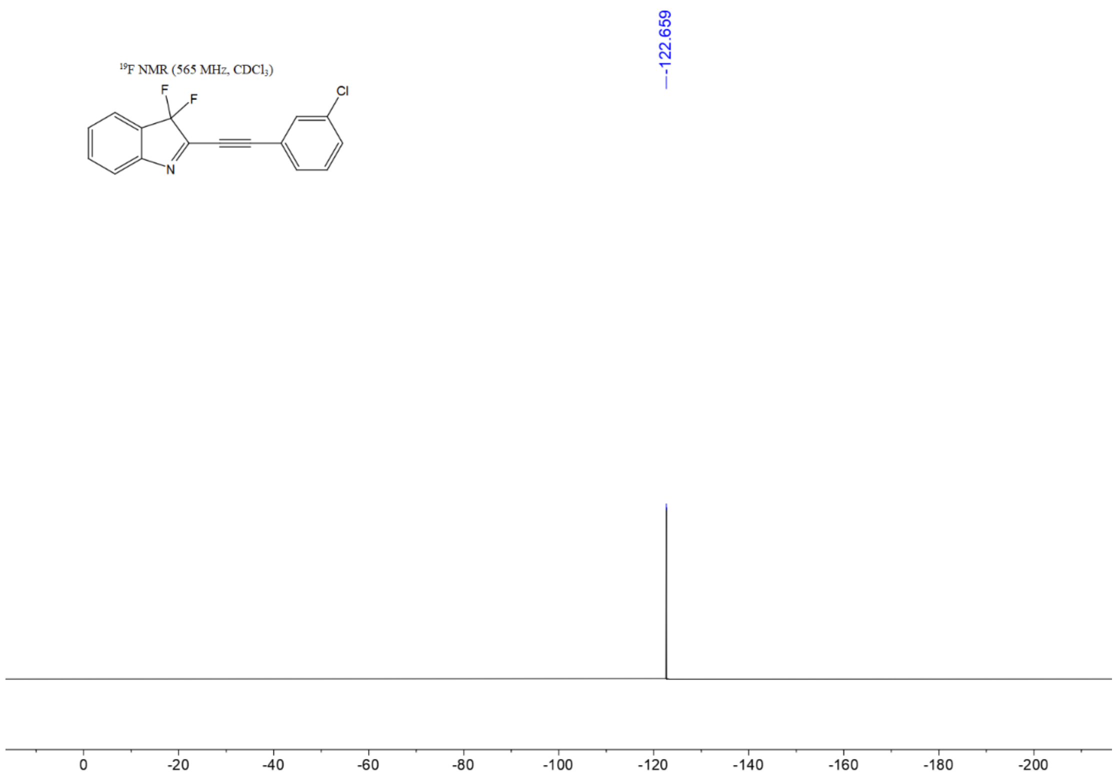

^{1}H NMR spectra of **1g**

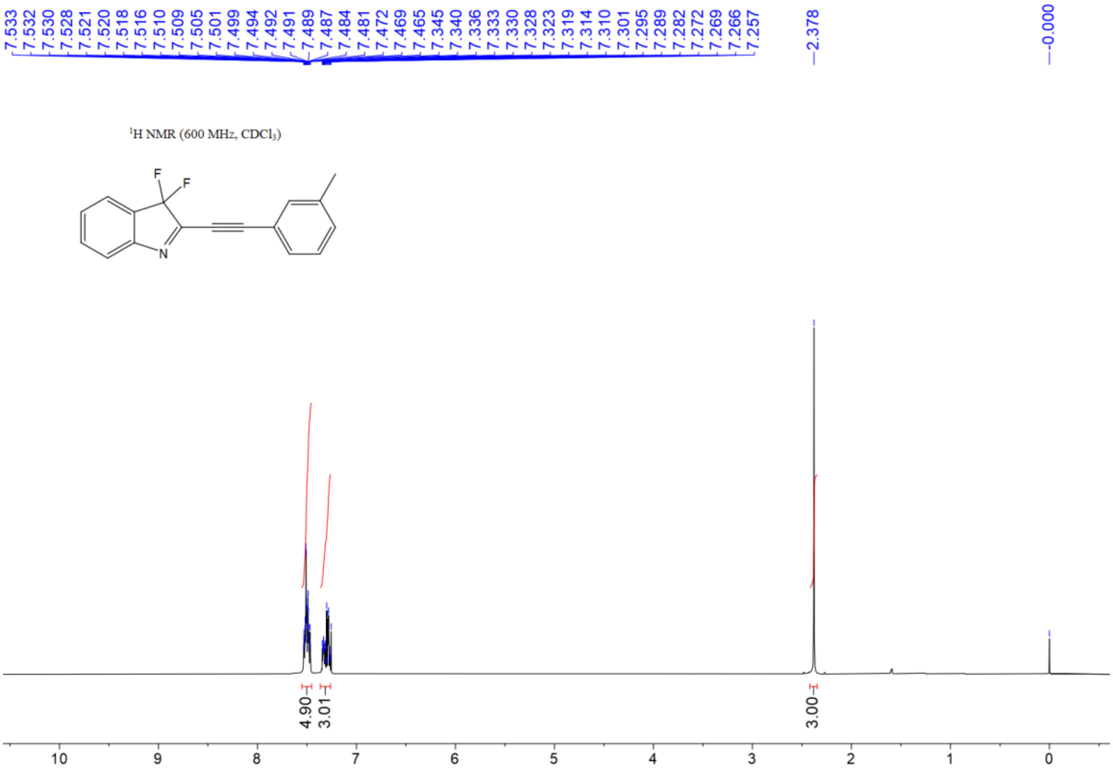

^{13}C NMR spectra of **1g**

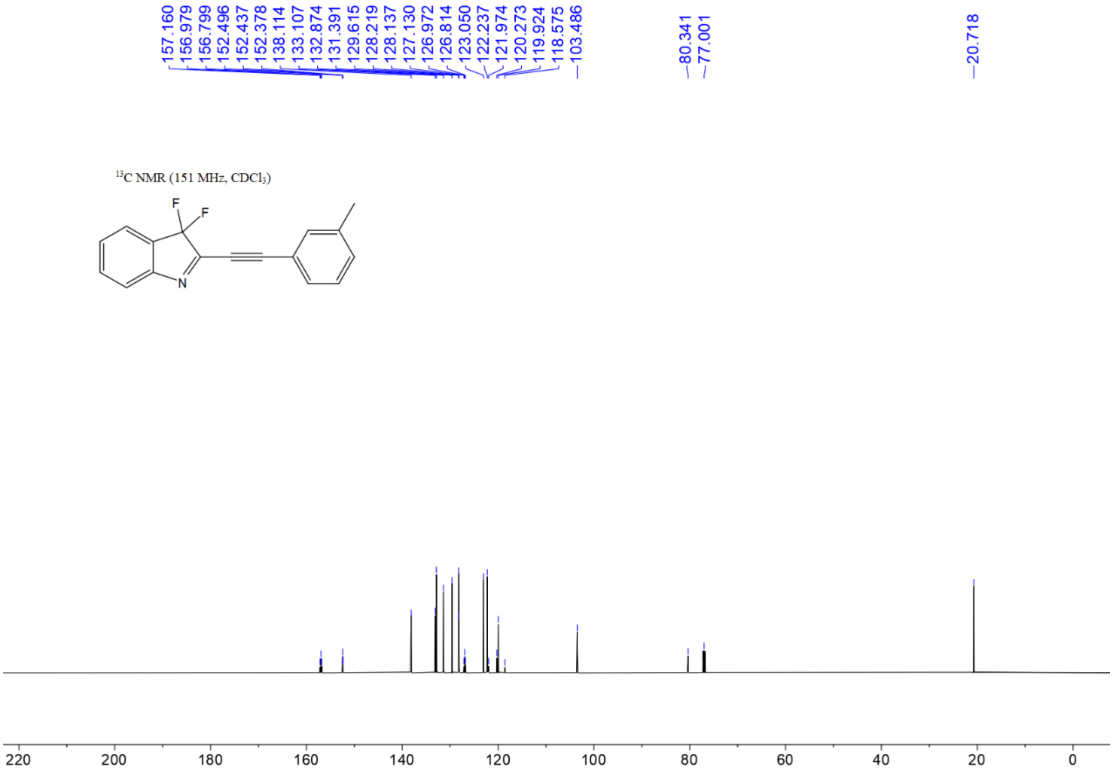

^{19}F NMR spectra of **1g**

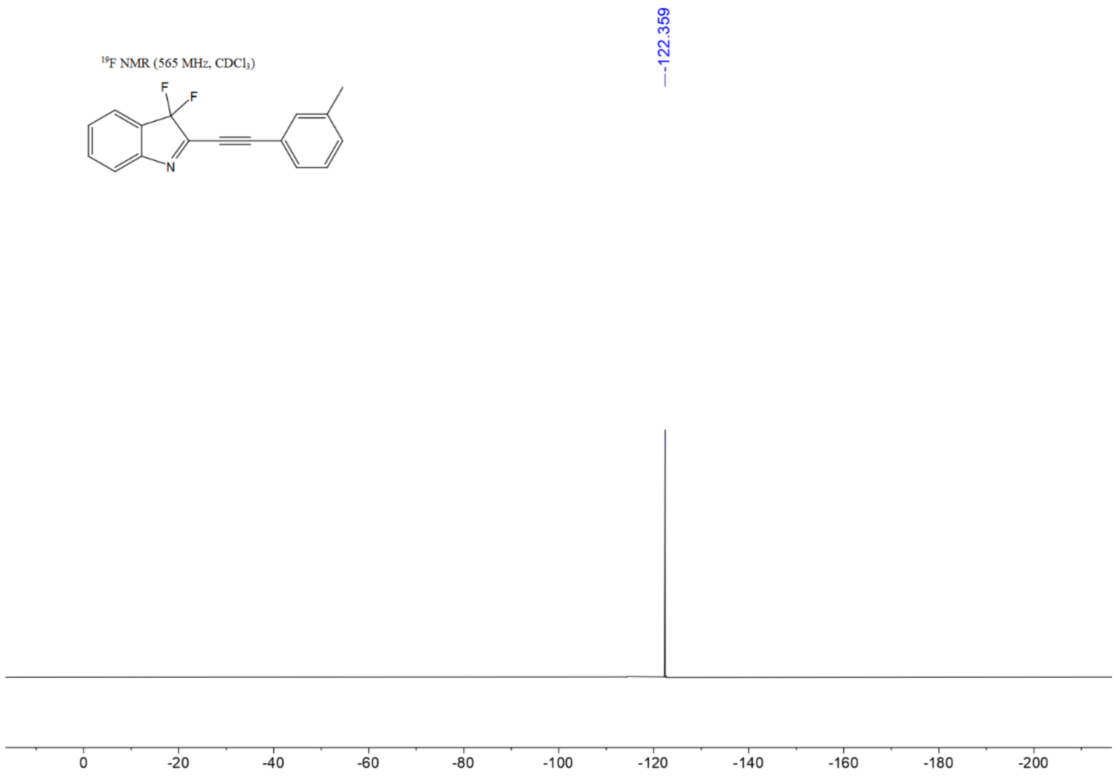

^{1}H NMR spectra of **1h**

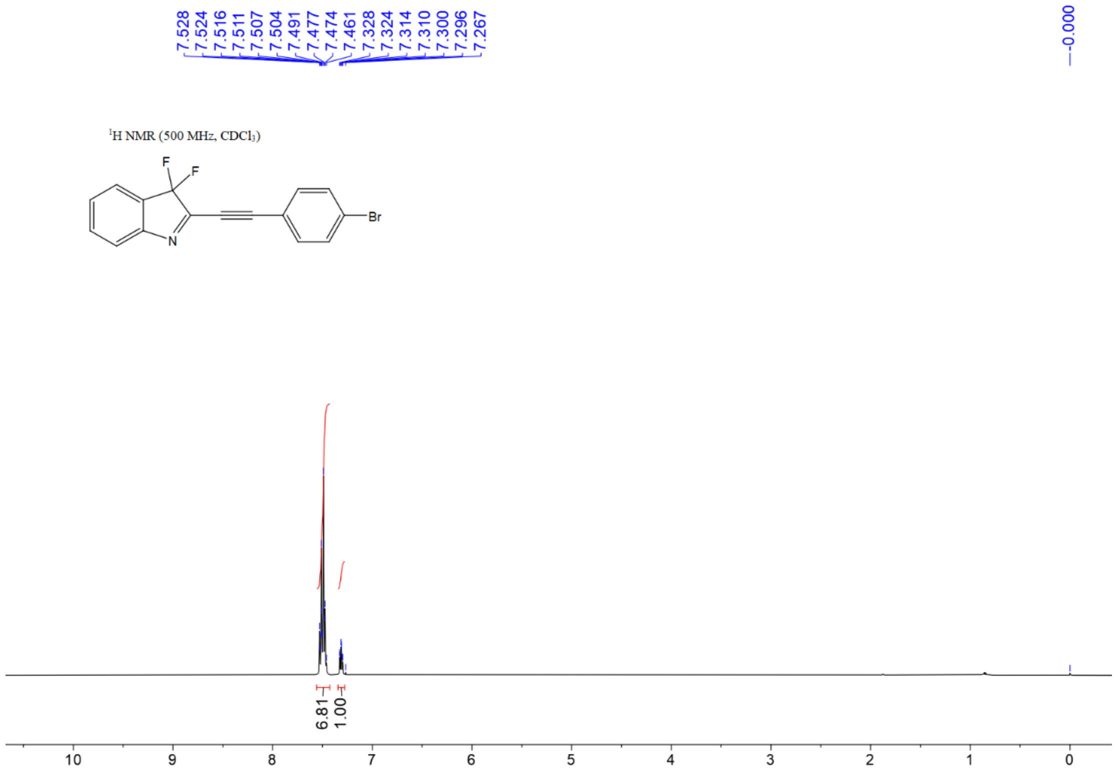

^{13}C NMR spectra of **1h**

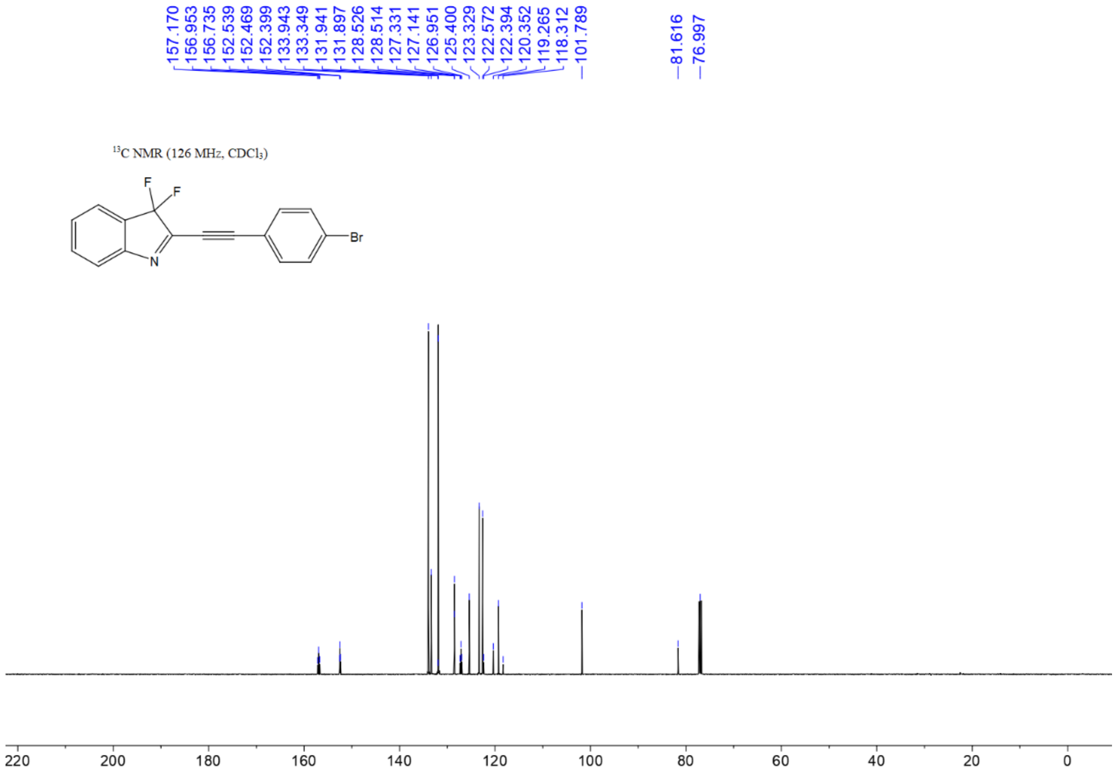

^{19}F NMR spectra of **1h**

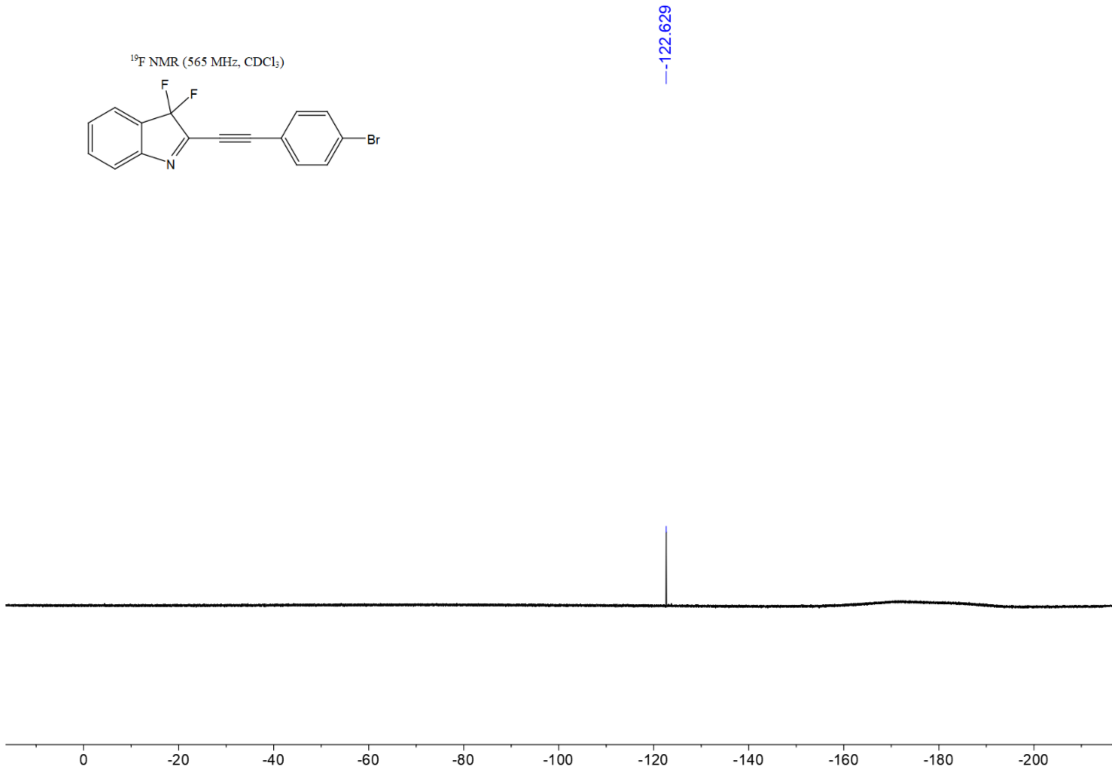

^{1}H NMR spectra of **1i**

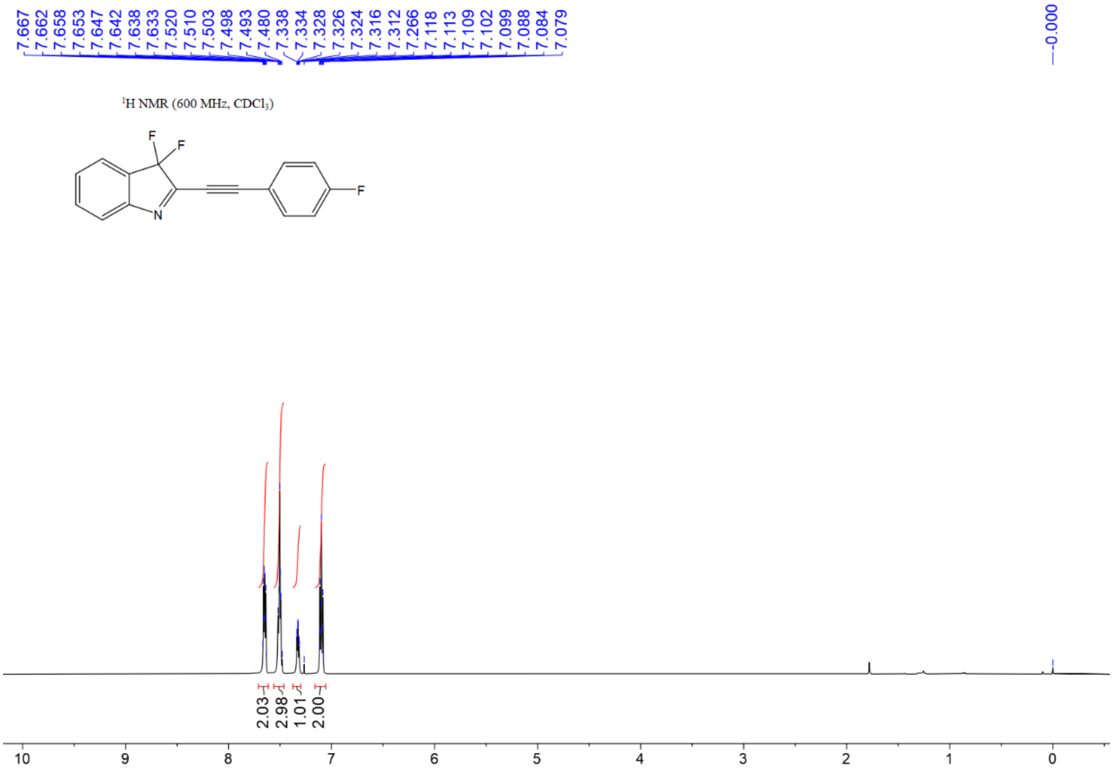

^{13}C NMR spectra of **1i**

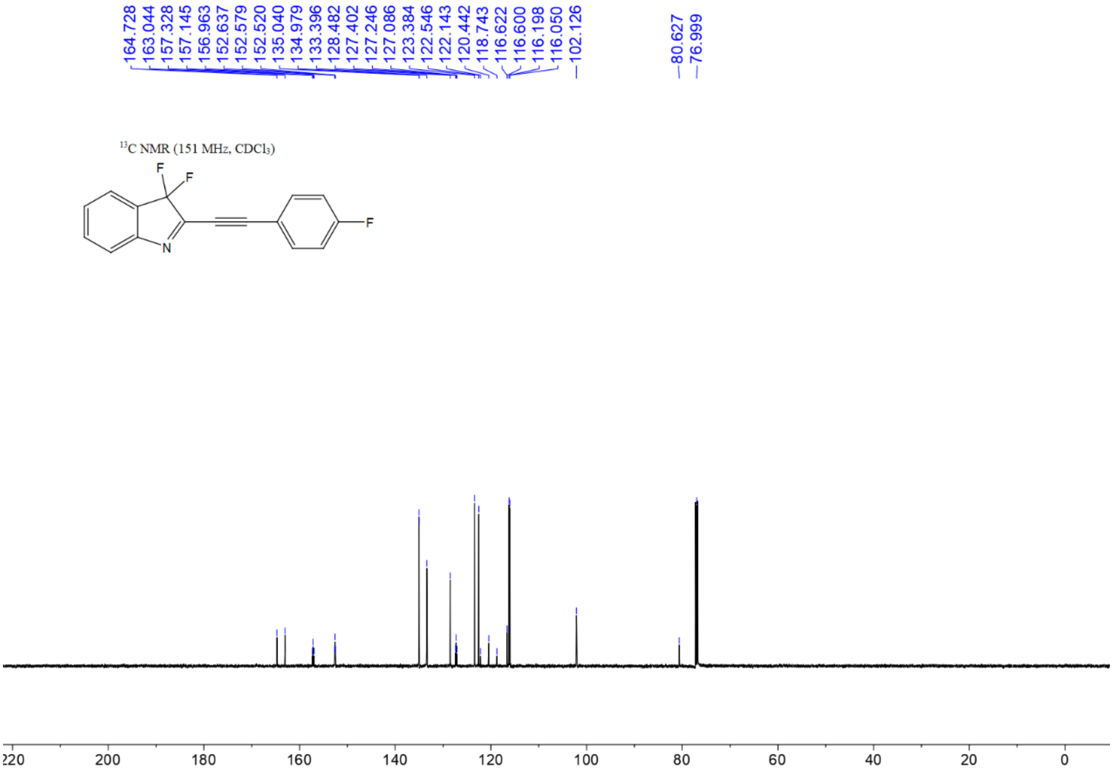

^{19}F NMR spectra of **1i**

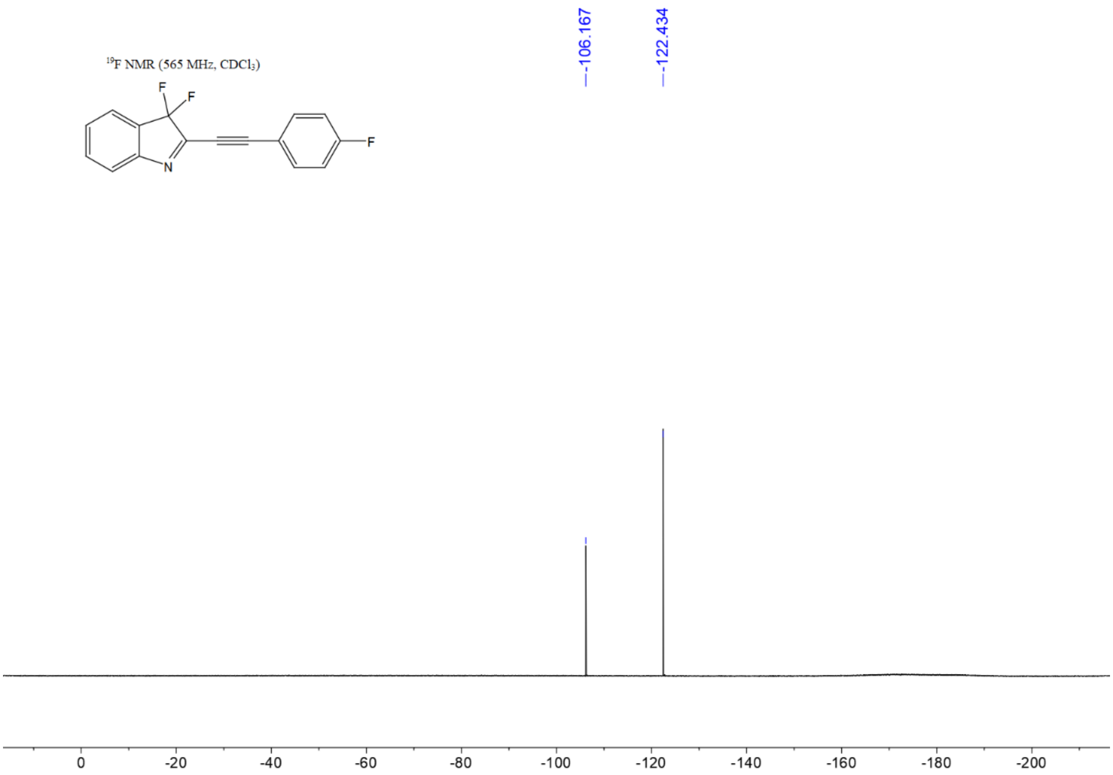

^{1}H NMR spectra of **1j**

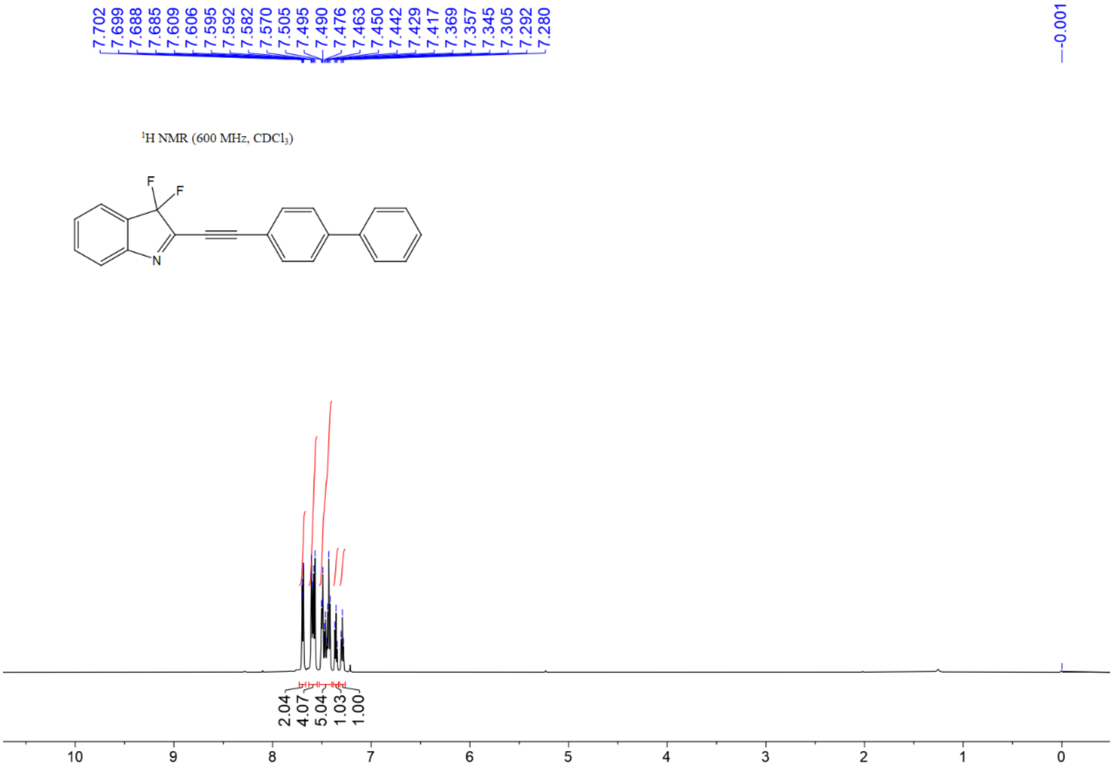

^{13}C NMR spectra of **1j**

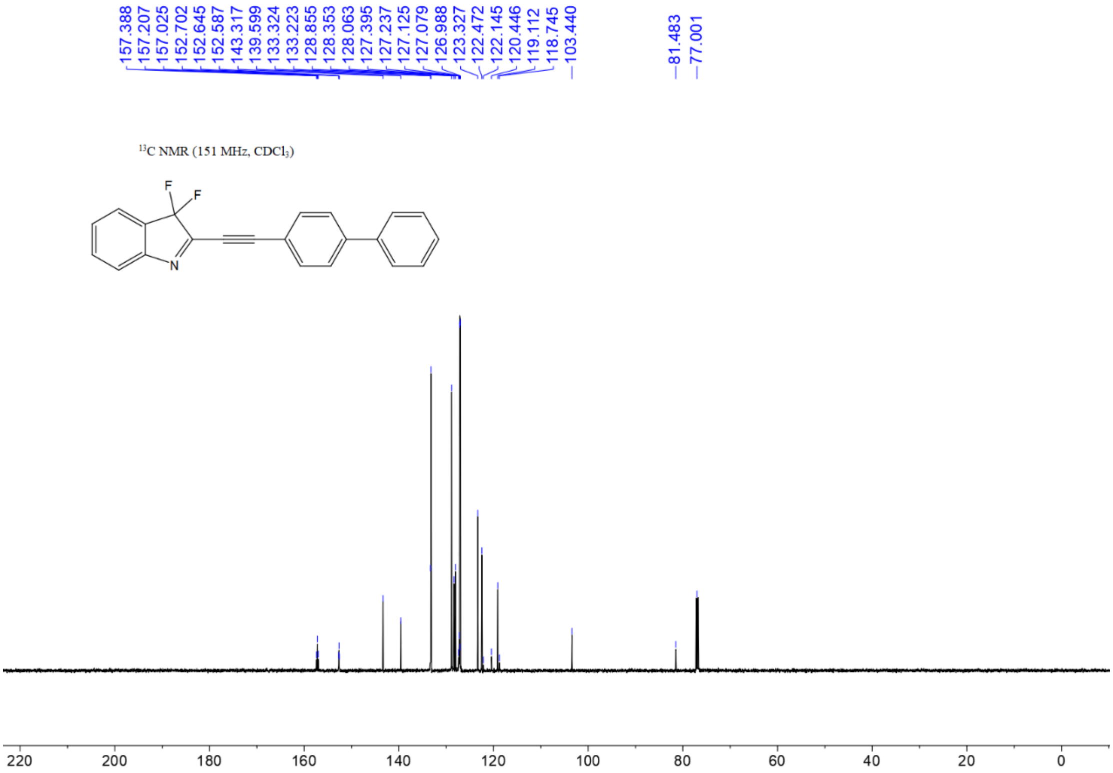

^{19}F NMR spectra of **1j**

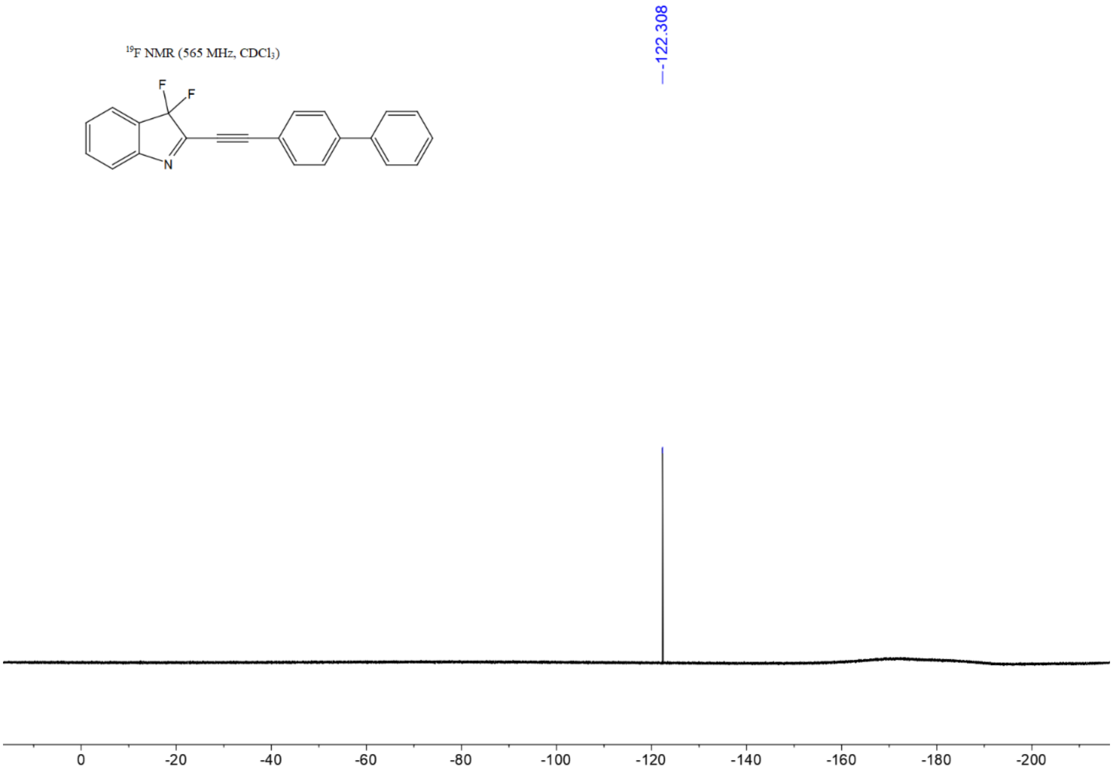

^{1}H NMR spectra of **1k**

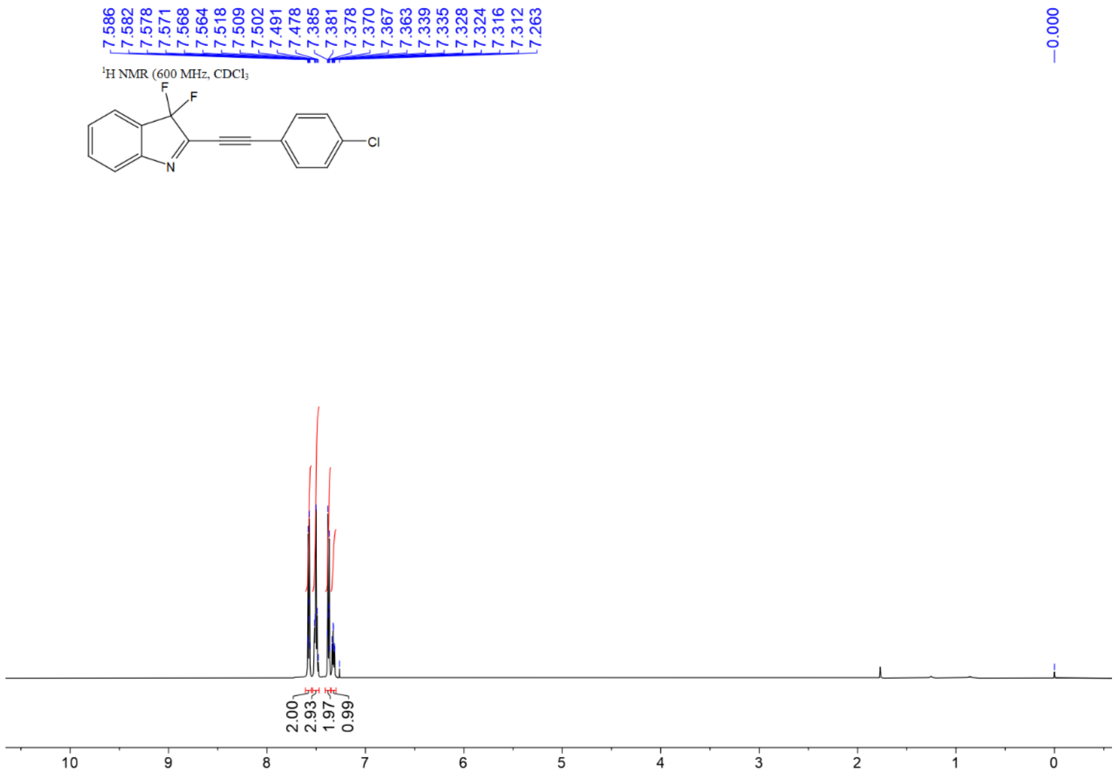

^{13}C NMR spectra of **1k**

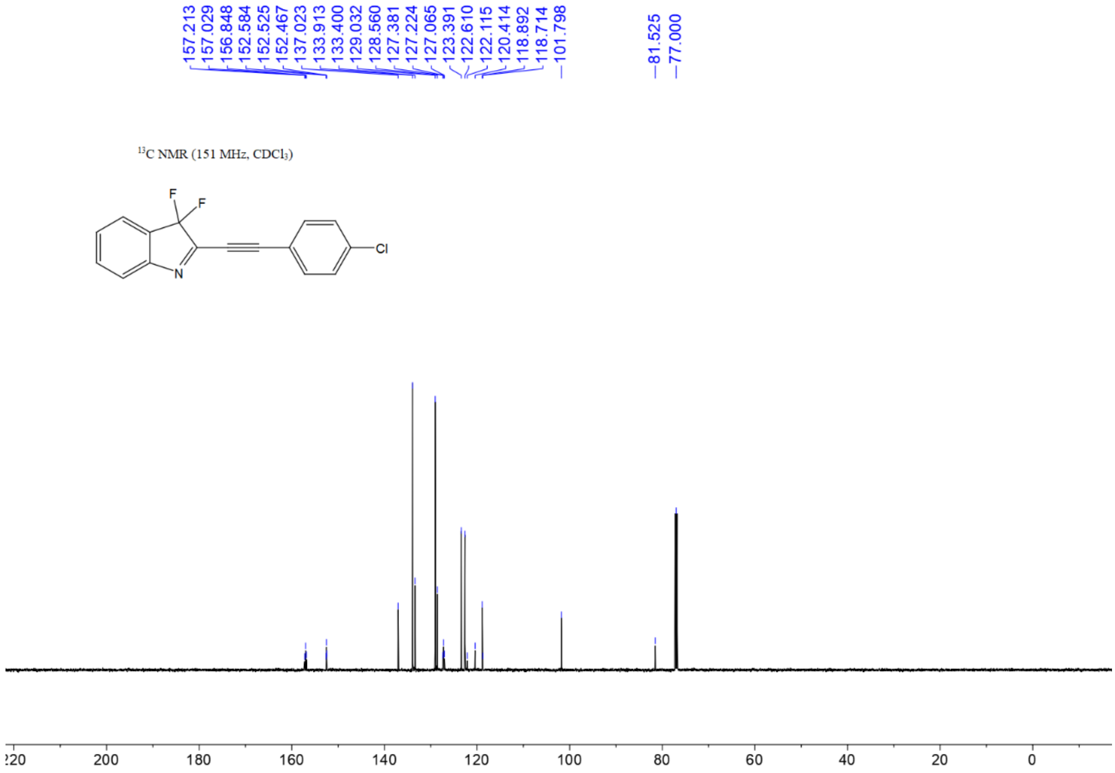

^{19}F NMR spectra of **1k**

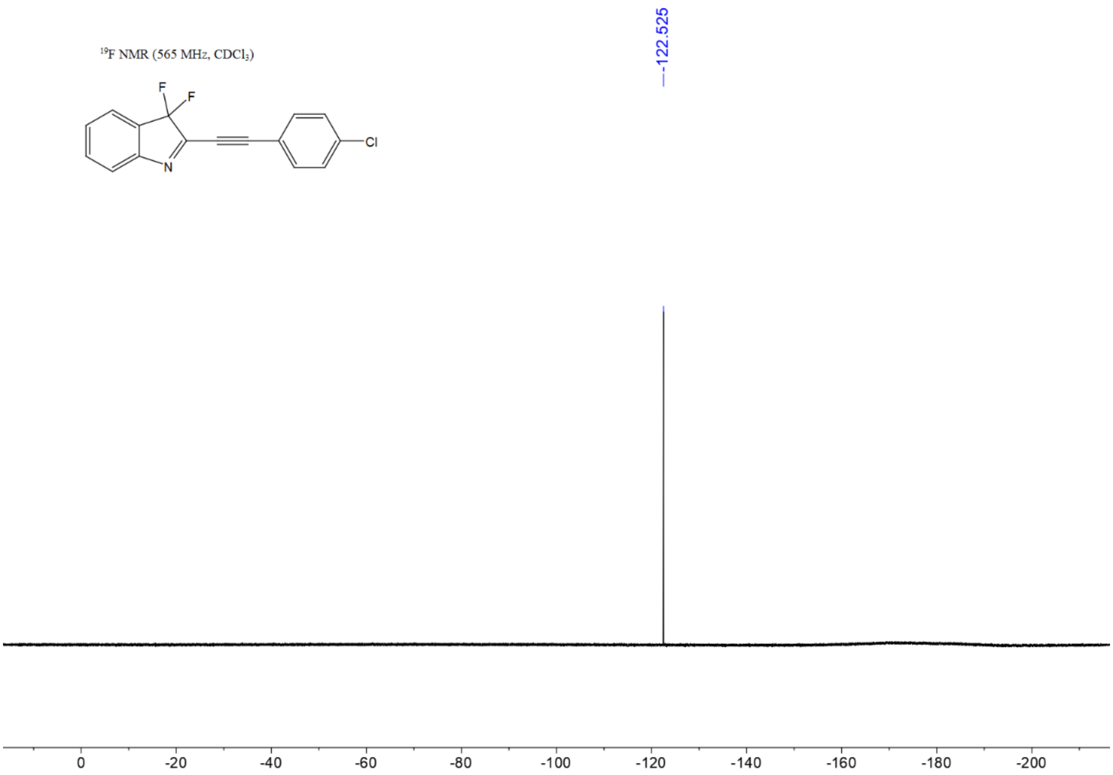

^{1}H NMR spectra of **1l**

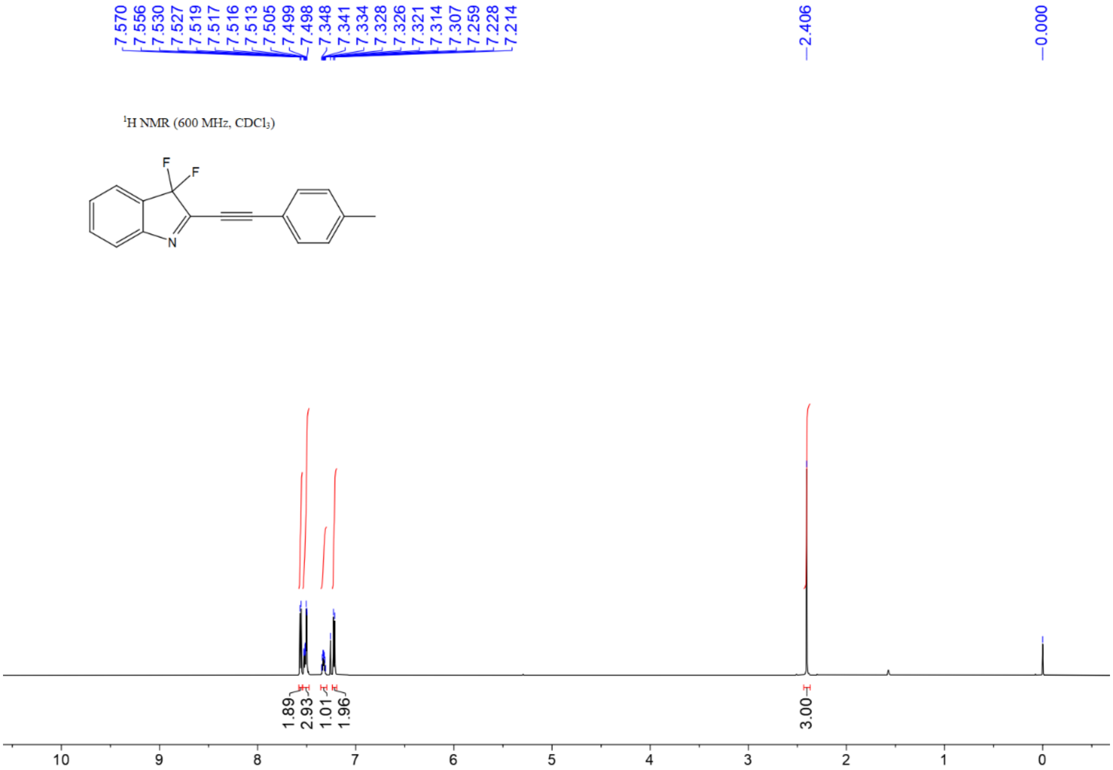

^{13}C NMR spectra of **1l**

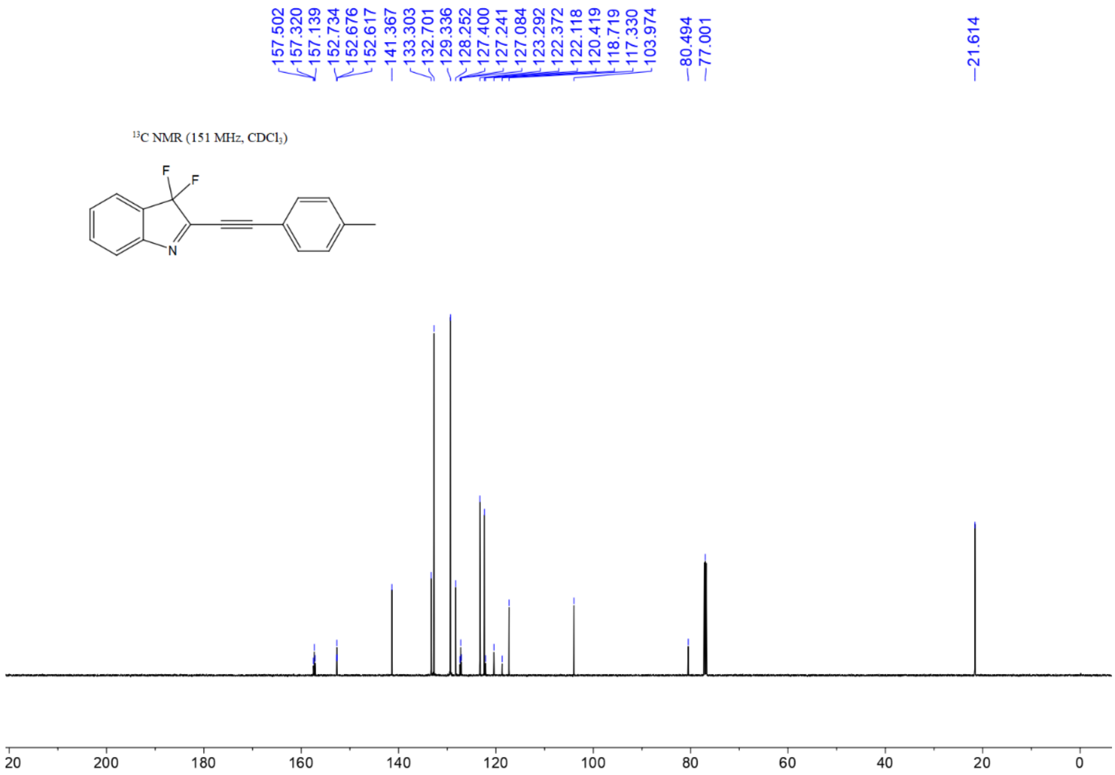

^{19}F NMR spectra of **1l**

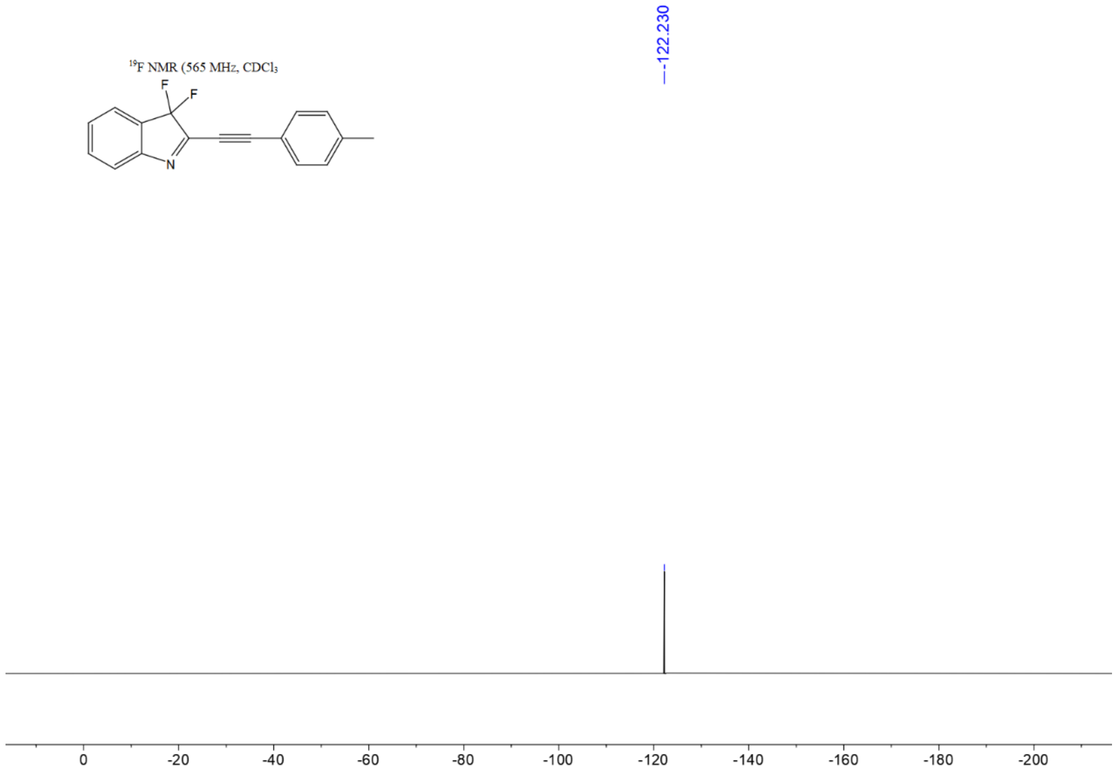

^{1}H NMR spectra of **1m**

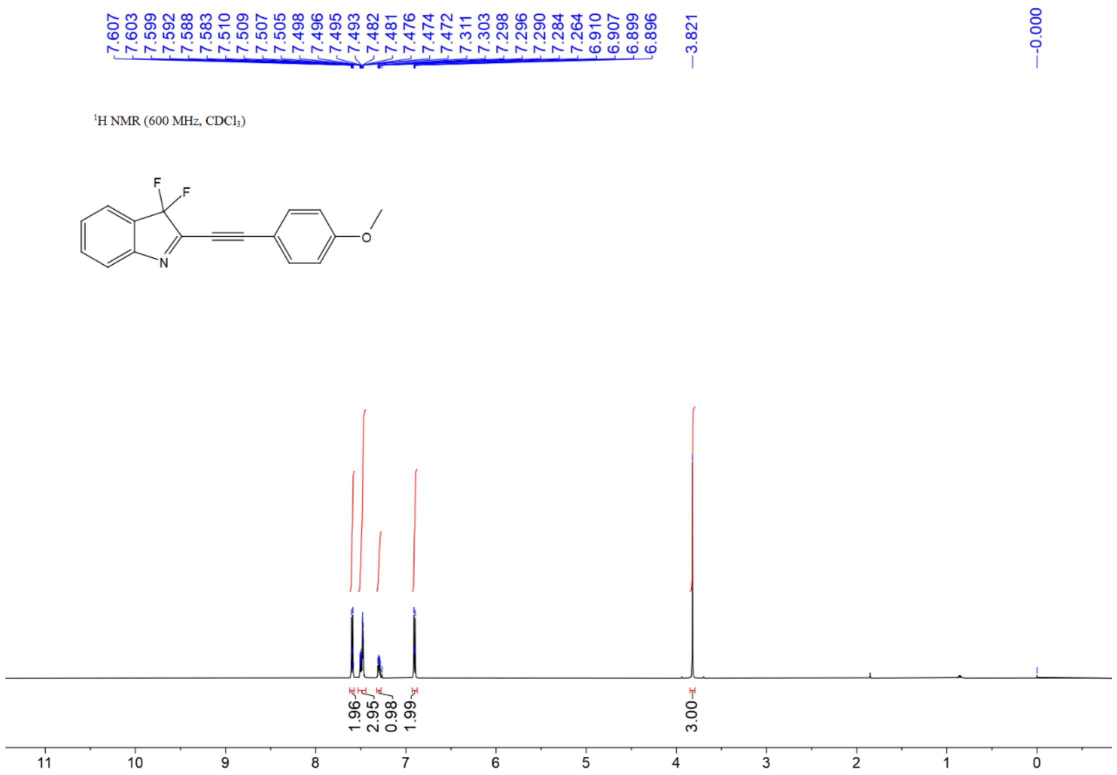

^{13}C NMR spectra of **1m**

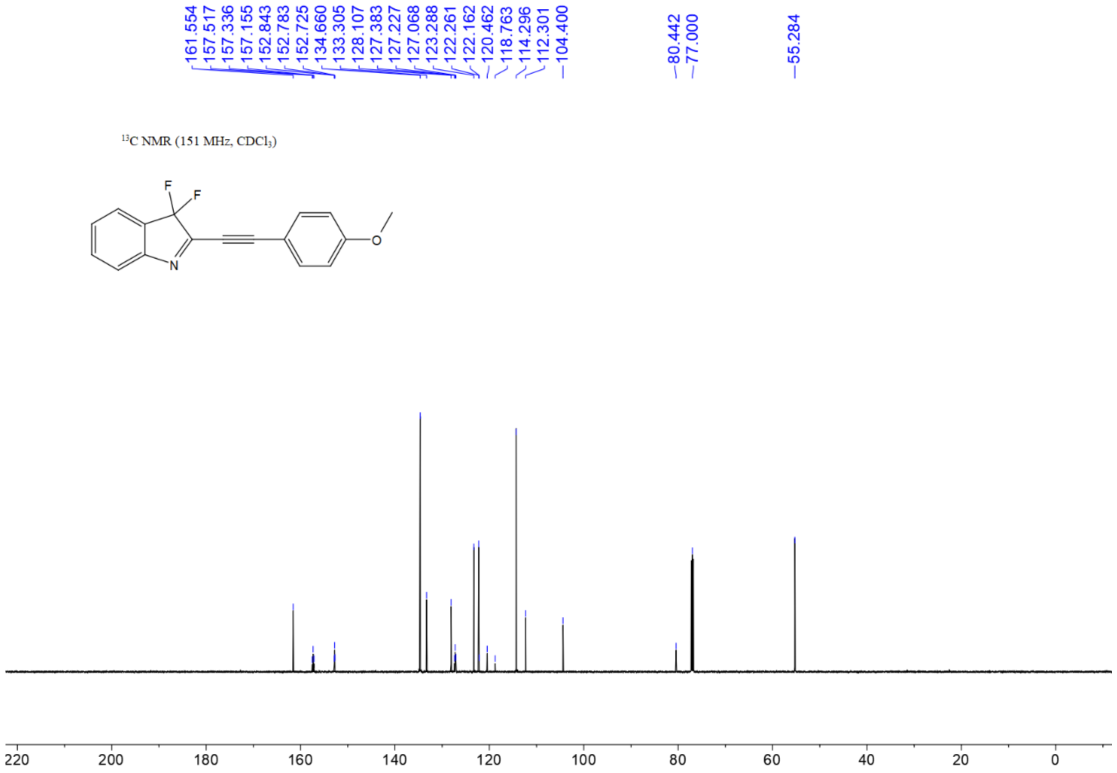

^{19}F NMR spectra of **1m**

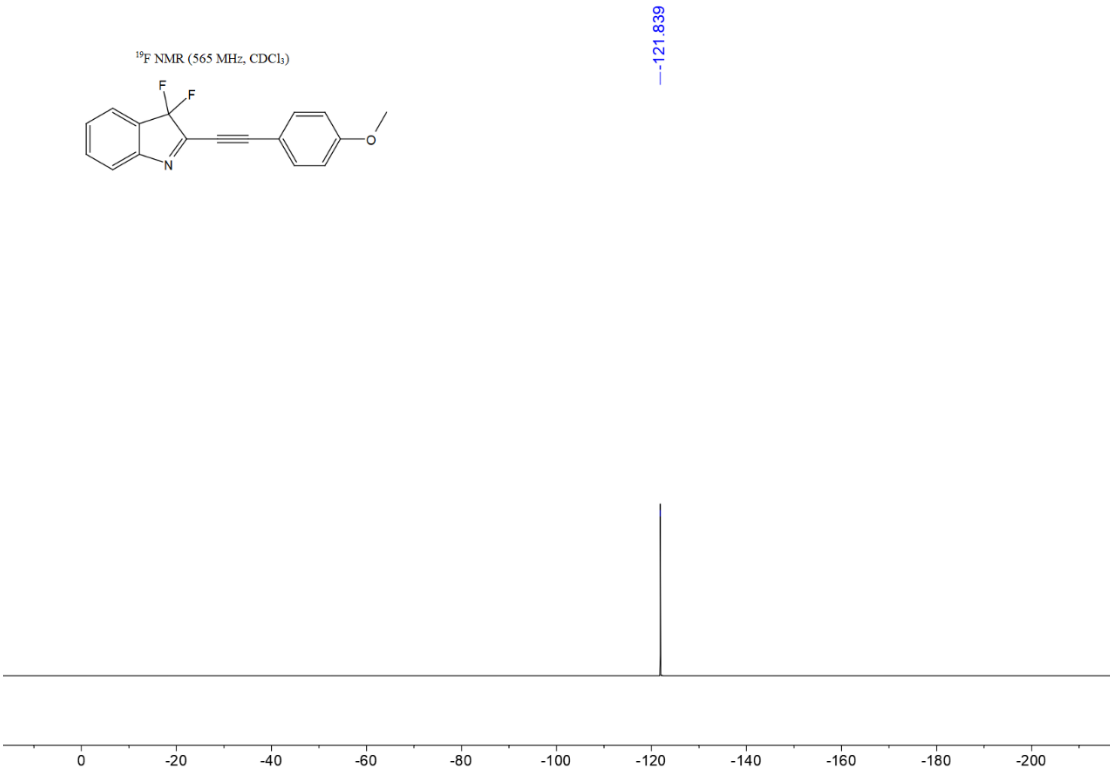

^{1}H NMR spectra of **1n**

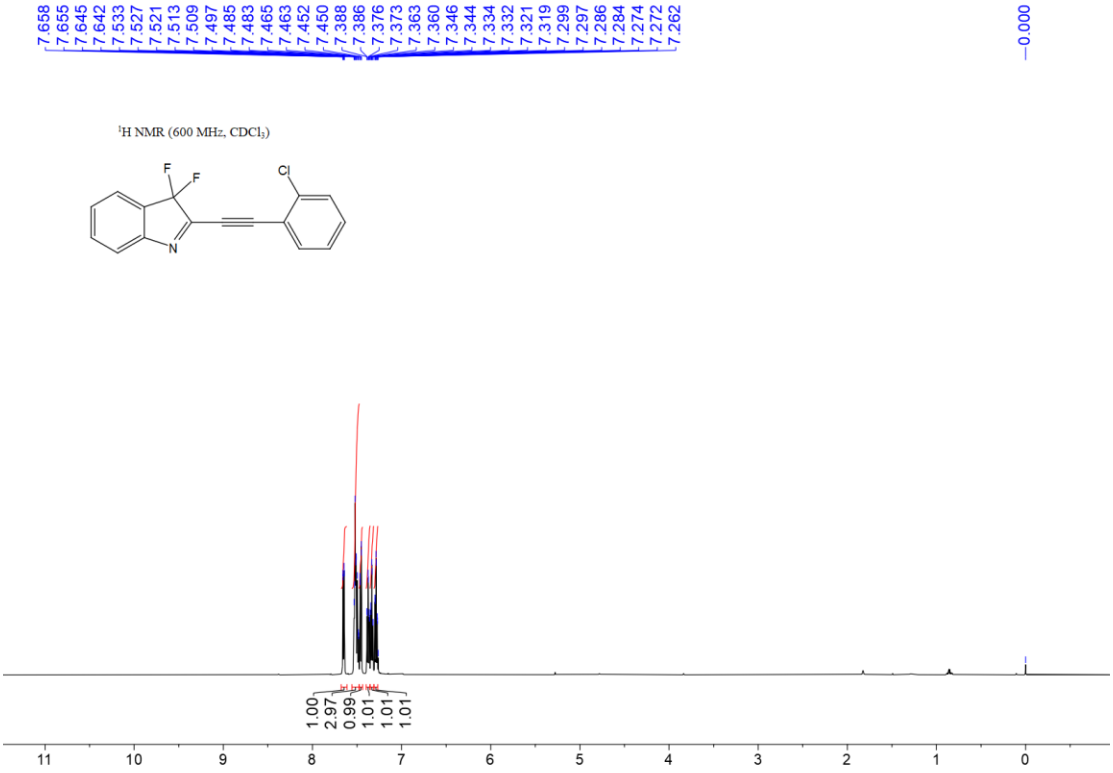

^{13}C NMR spectra of **1n**

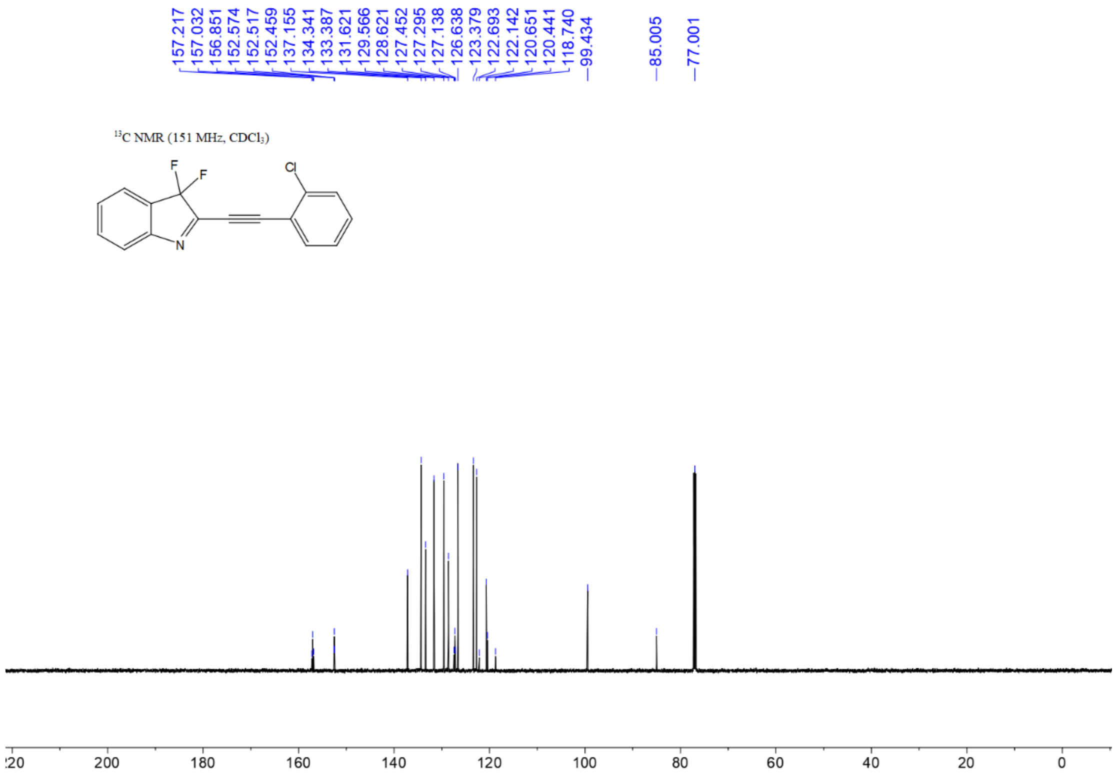

^{19}F NMR spectra of **1n**

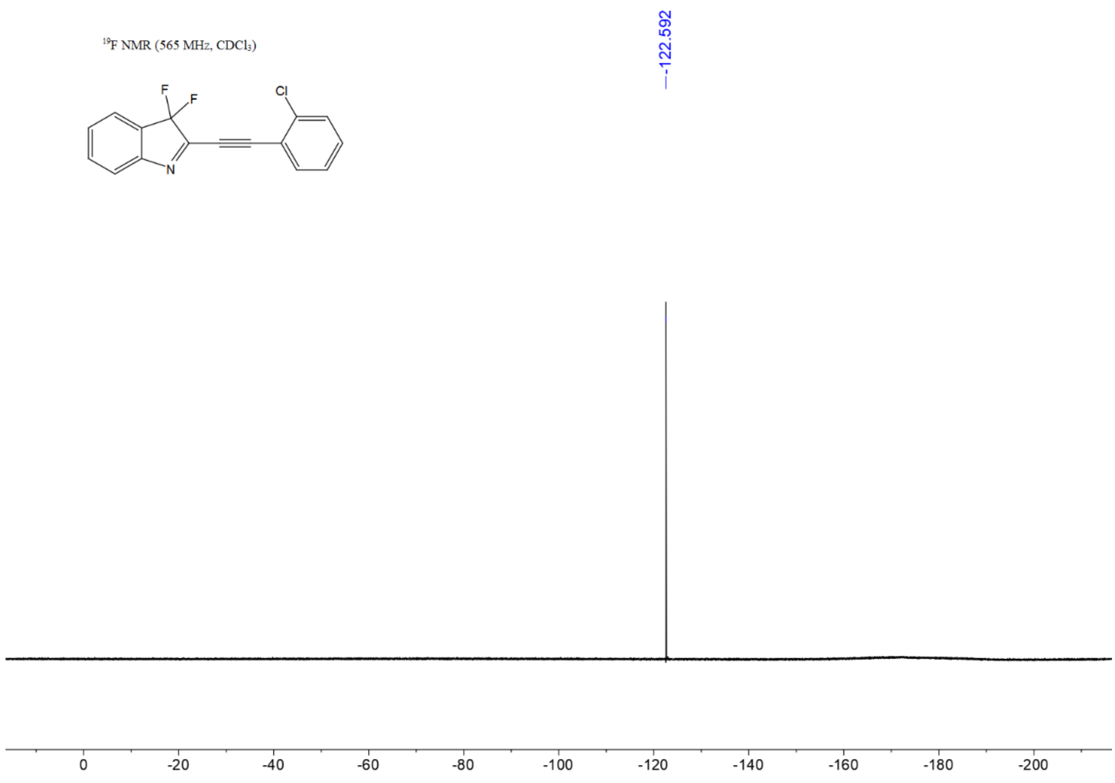

^{1}H NMR spectra of **1o**

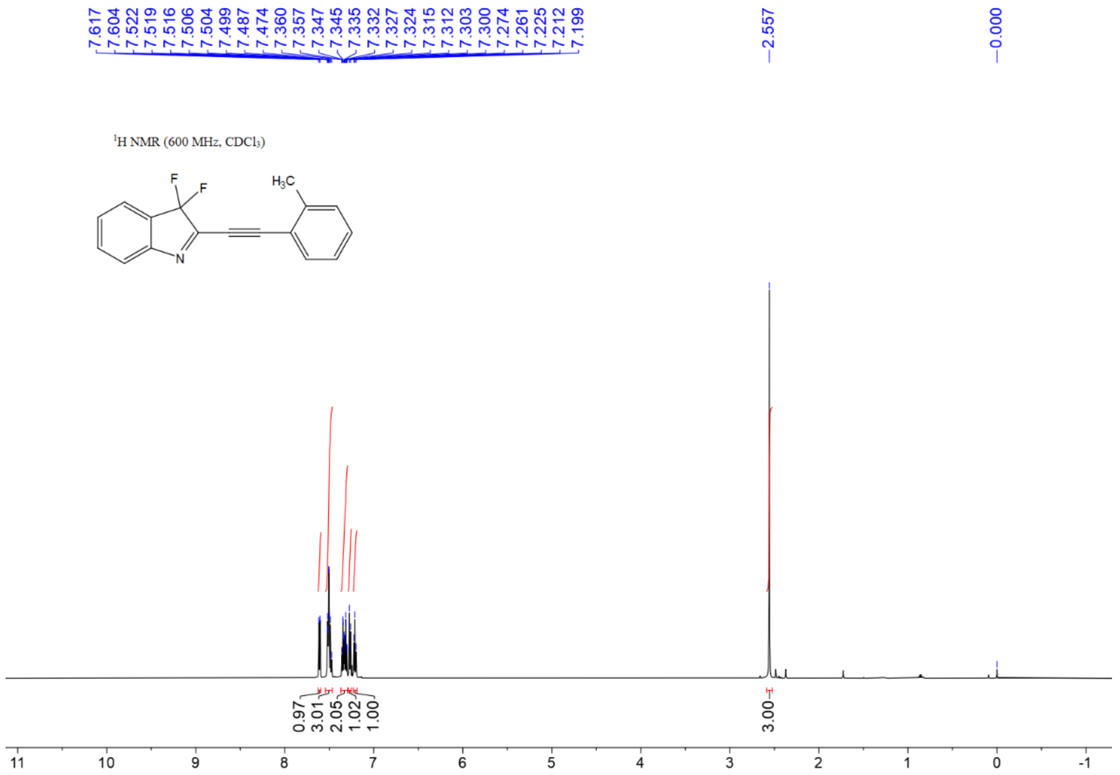

^{13}C NMR spectra of **1o**

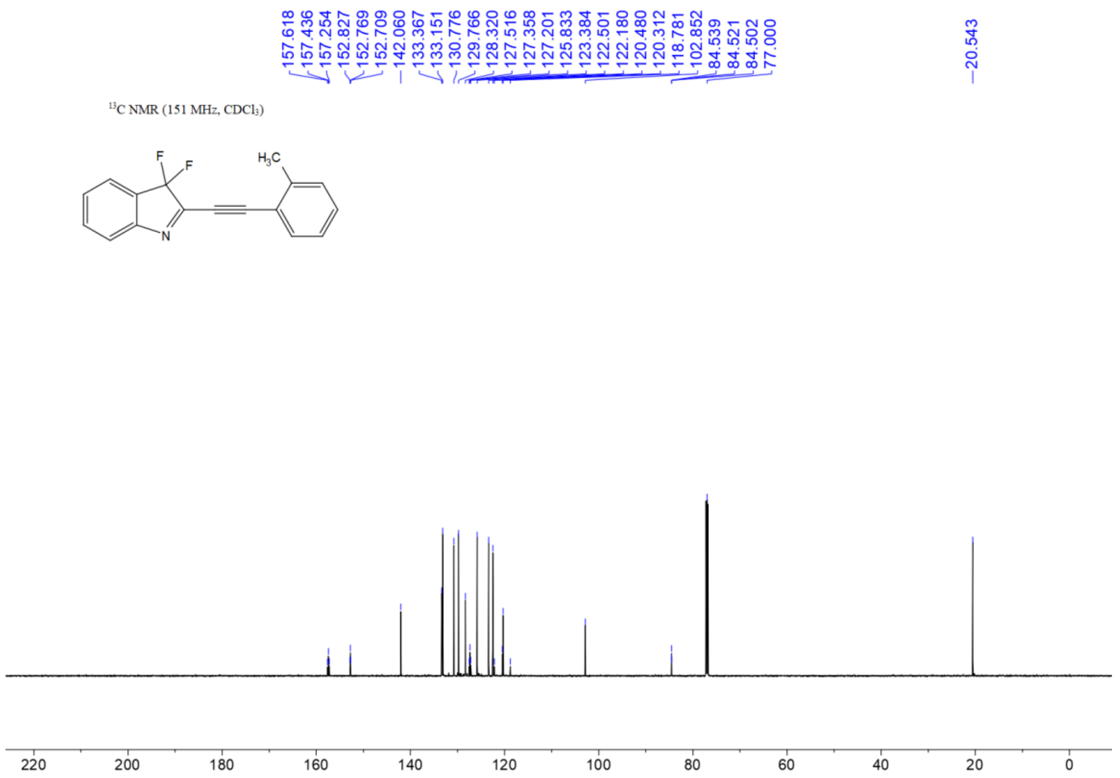

^{19}F NMR spectra of **1o**

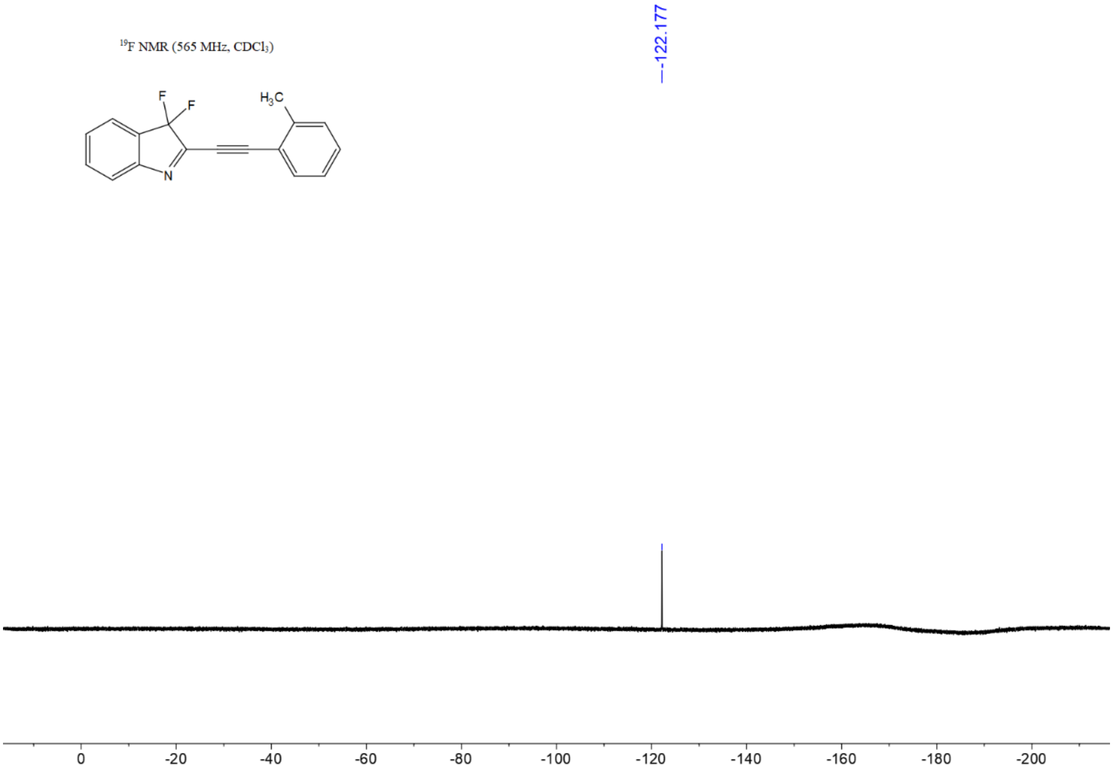

^{1}H NMR spectra of **1p**

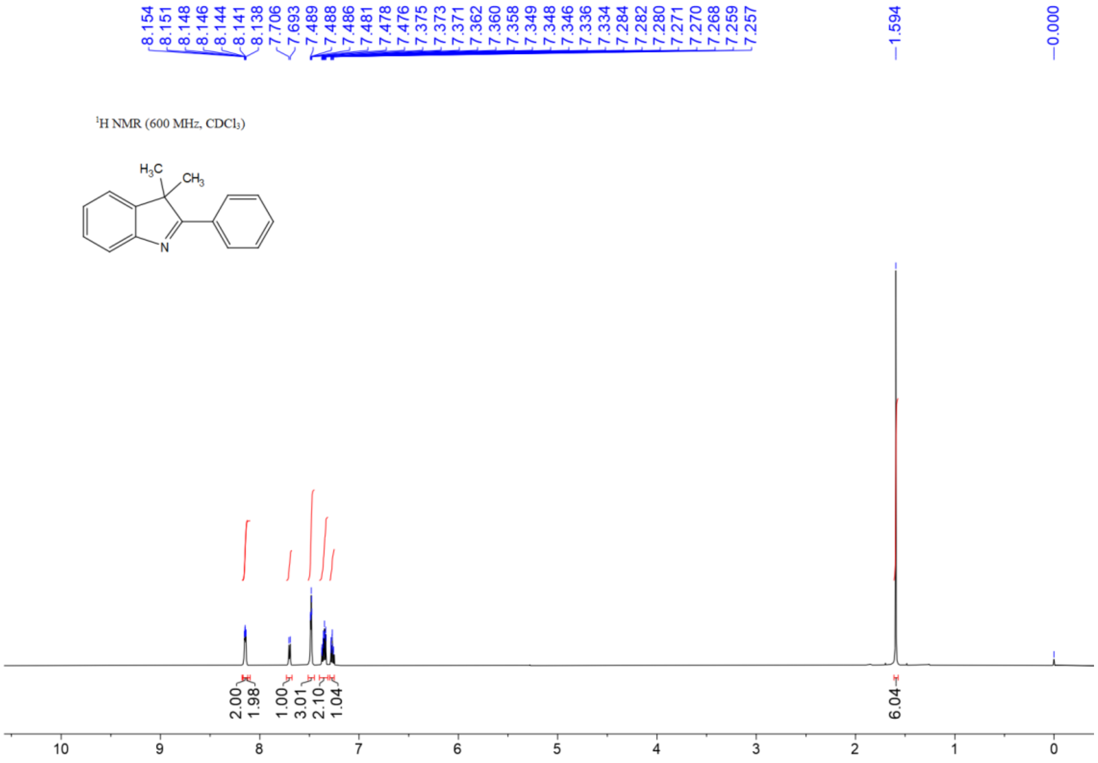

^{13}C NMR spectra of **1p**

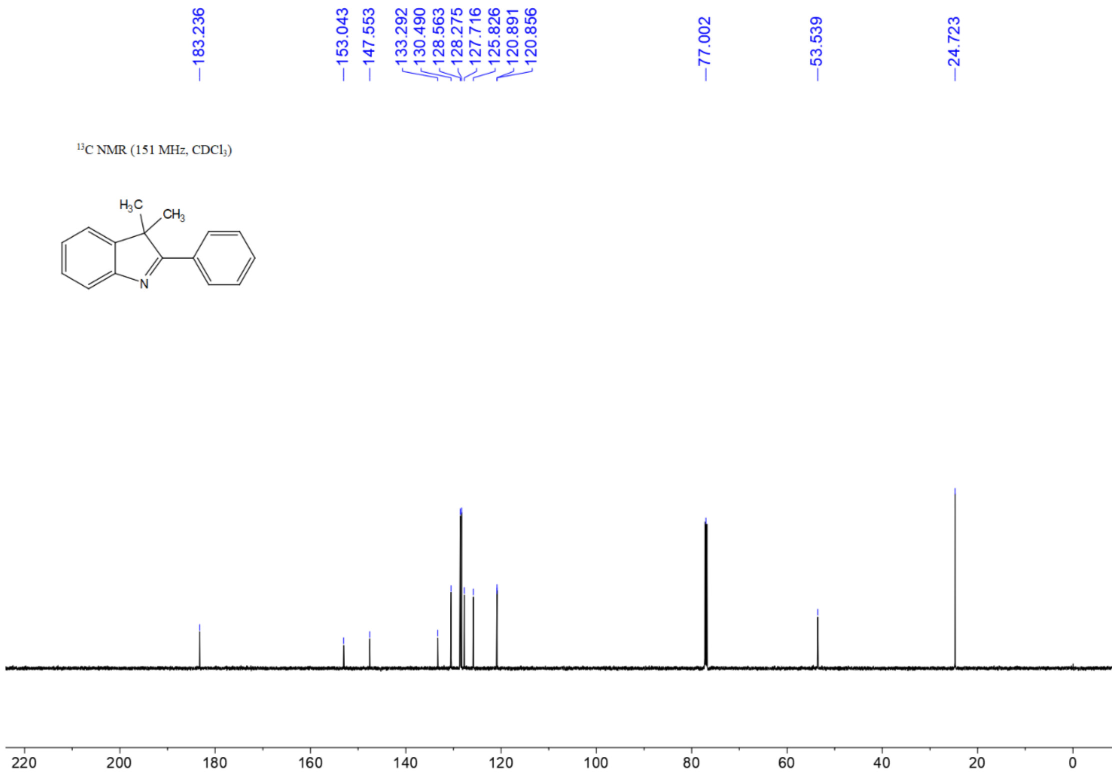

^{1}H NMR spectra of **1q**

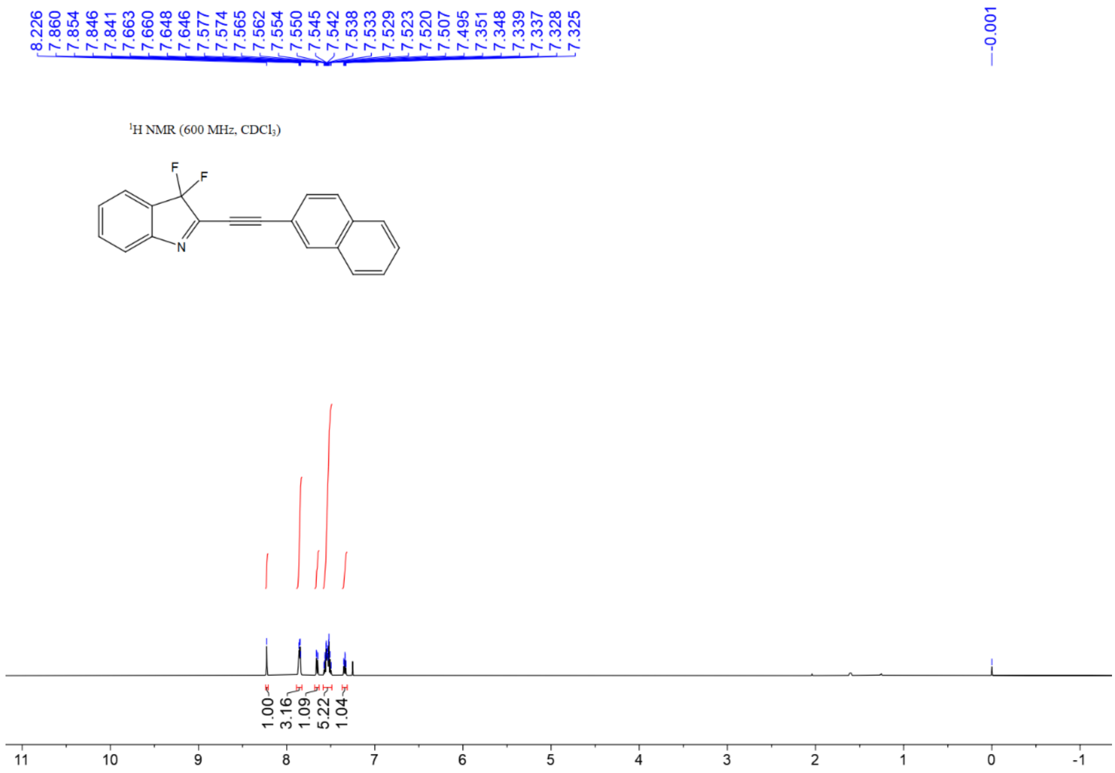

^{13}C NMR spectra of **1q**

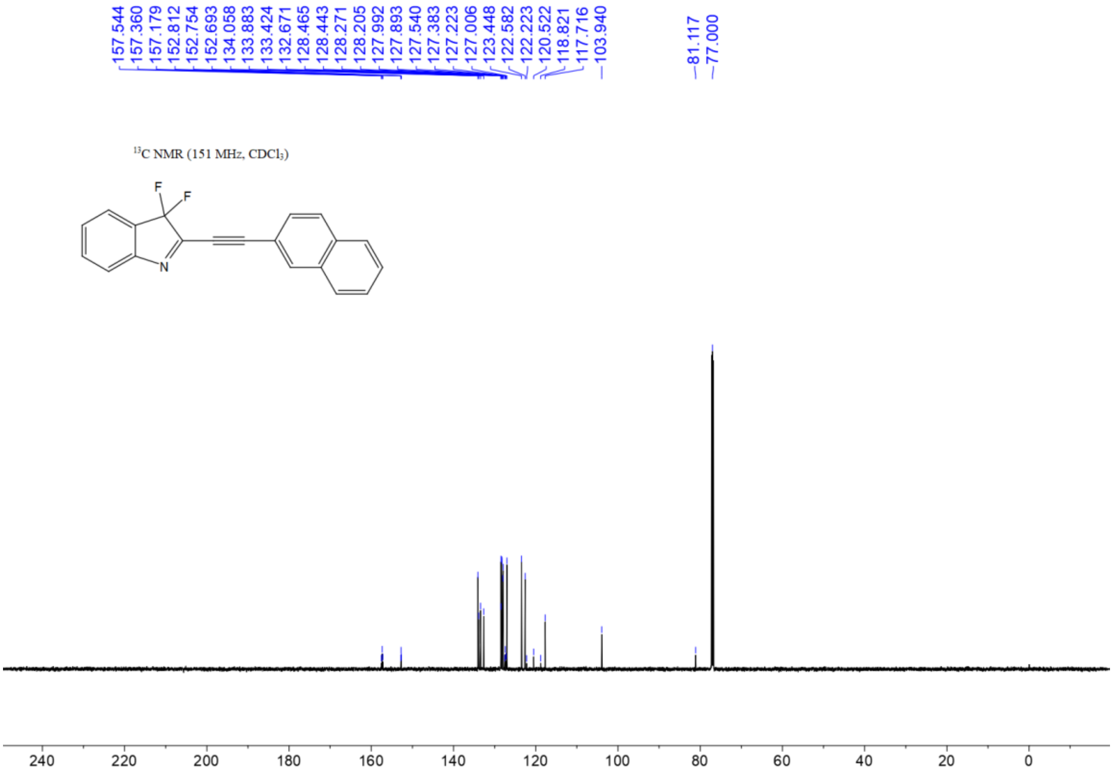

^{19}F NMR spectra of **1q**

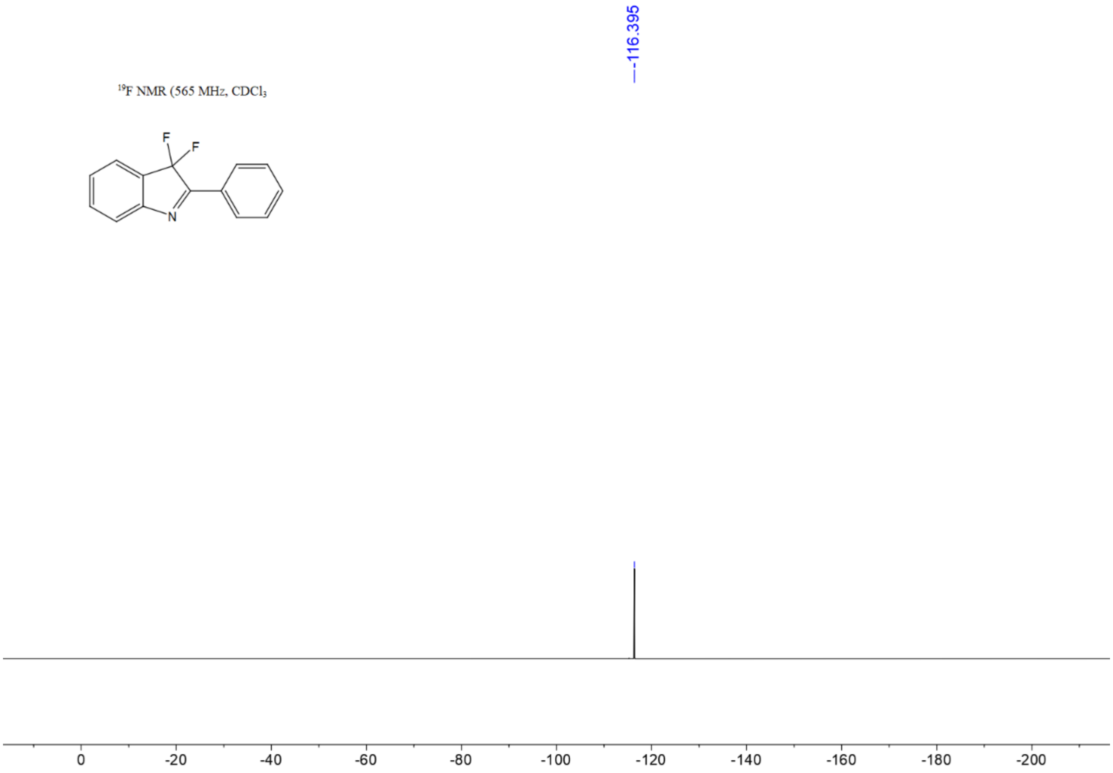

^{1}H NMR spectra of **1r**

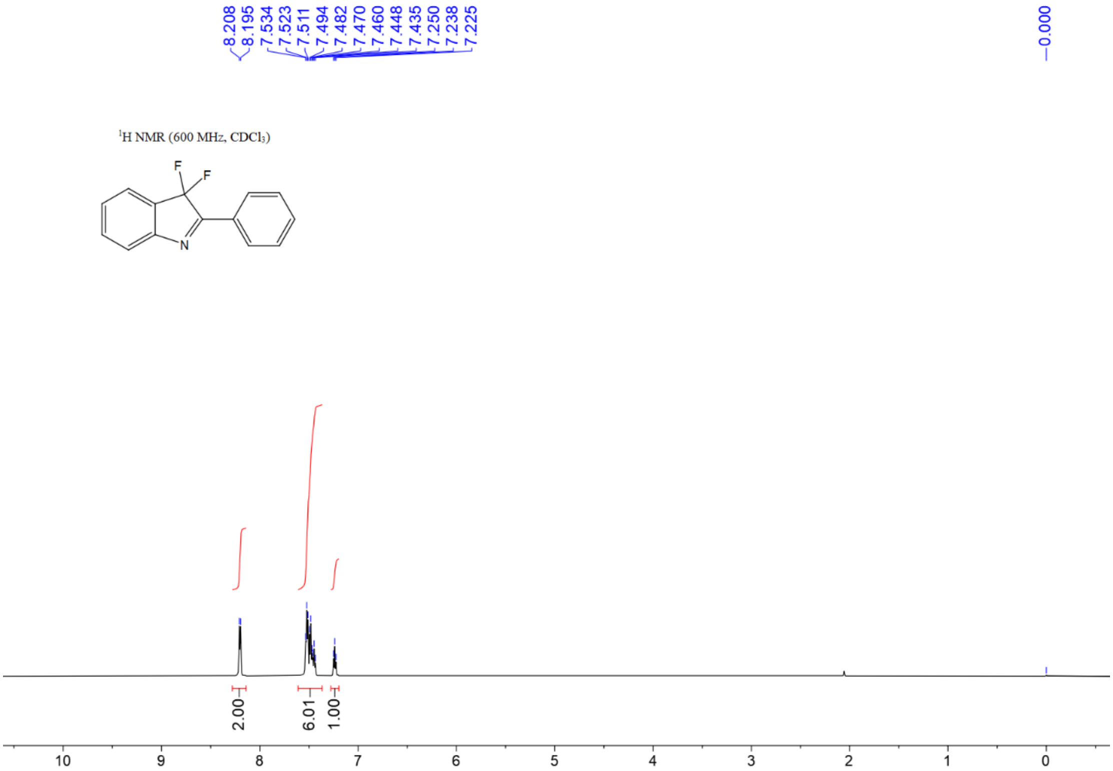

^{13}C NMR spectra of **1r**

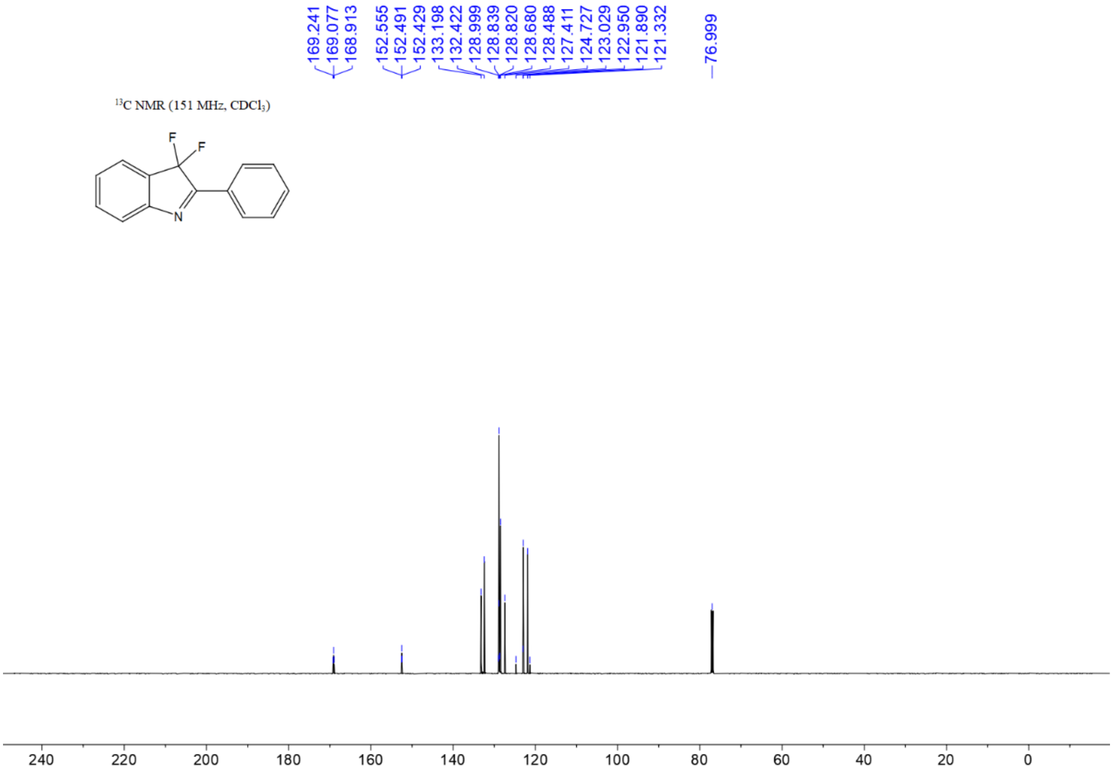

^{19}F NMR spectra of **1r**

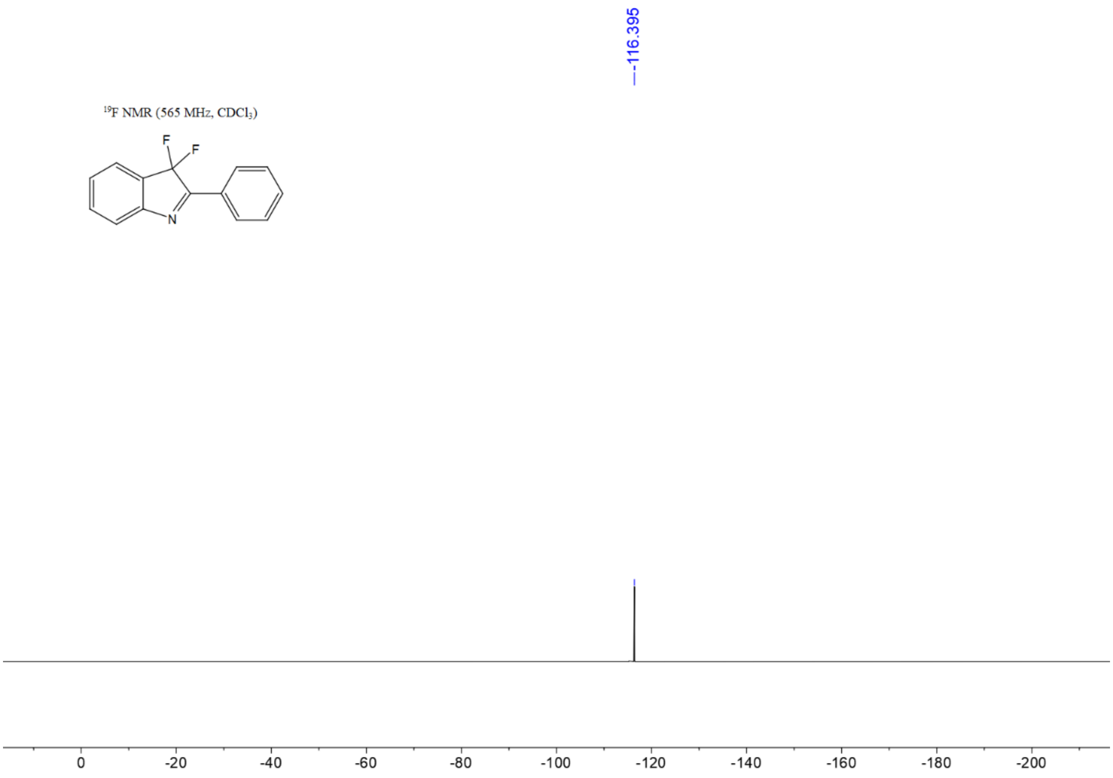

^{1}H NMR spectra of **2a**

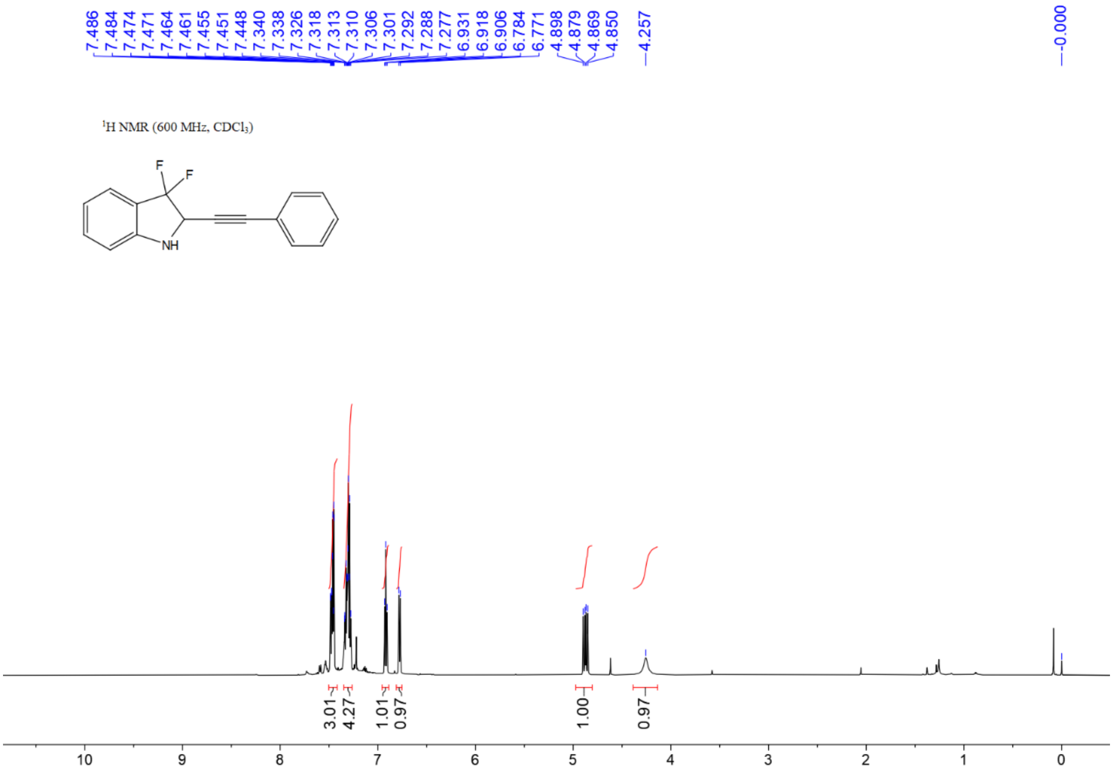

^{13}C NMR spectra of **2a**

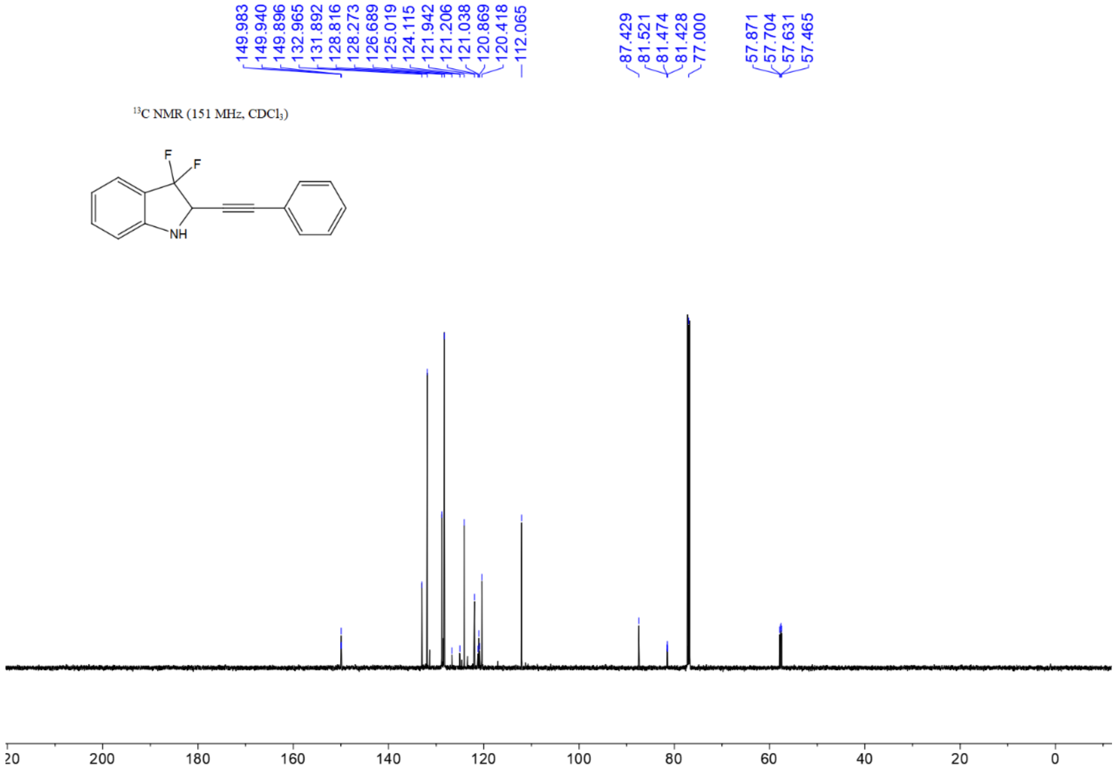

^{19}F NMR spectra of **2a**

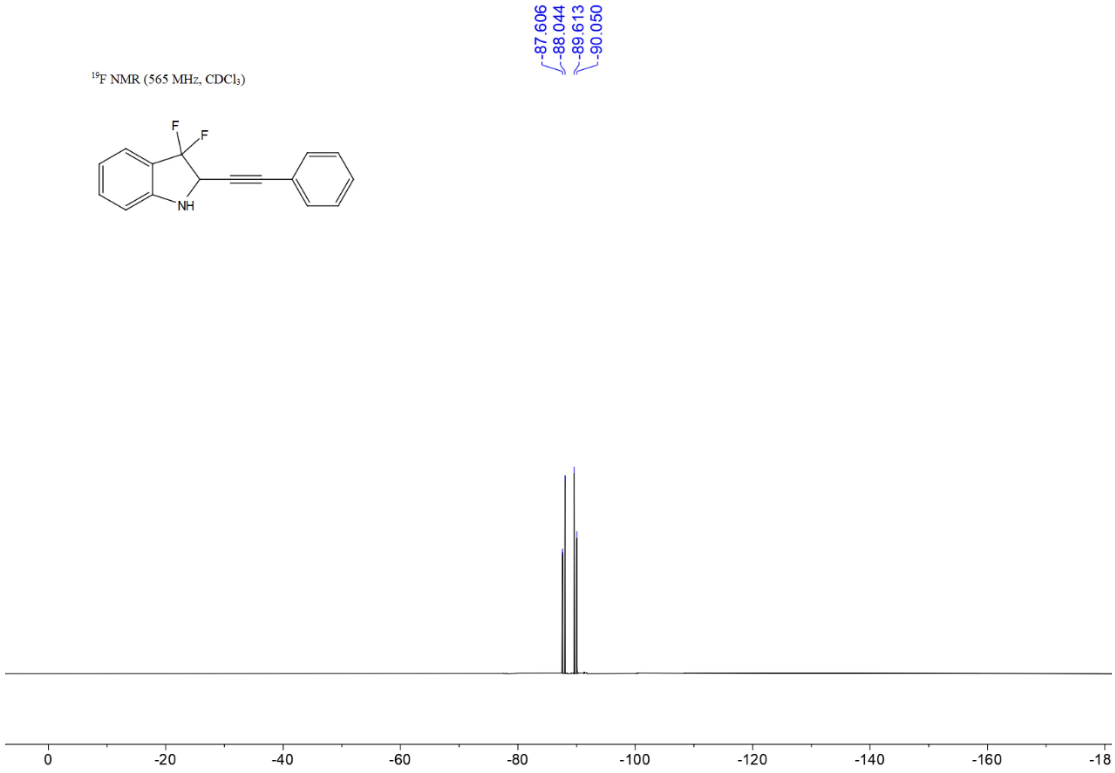

^{1}H NMR spectra of **2b**

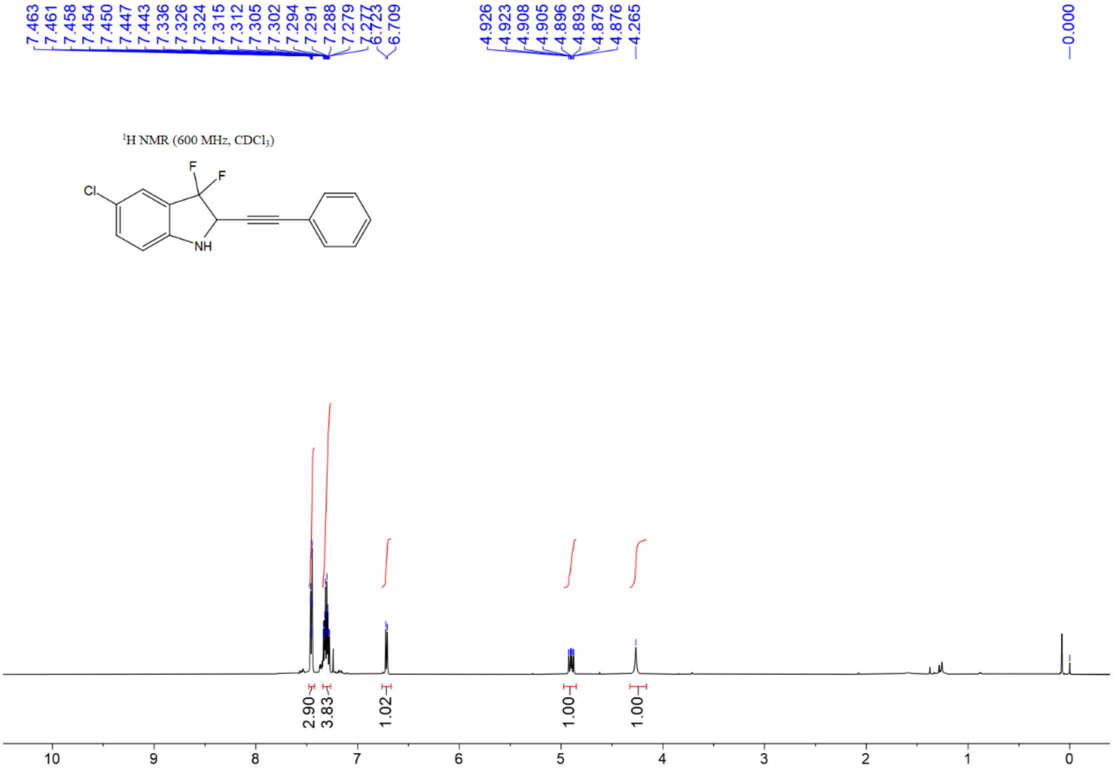

^{13}C NMR spectra of **2b**

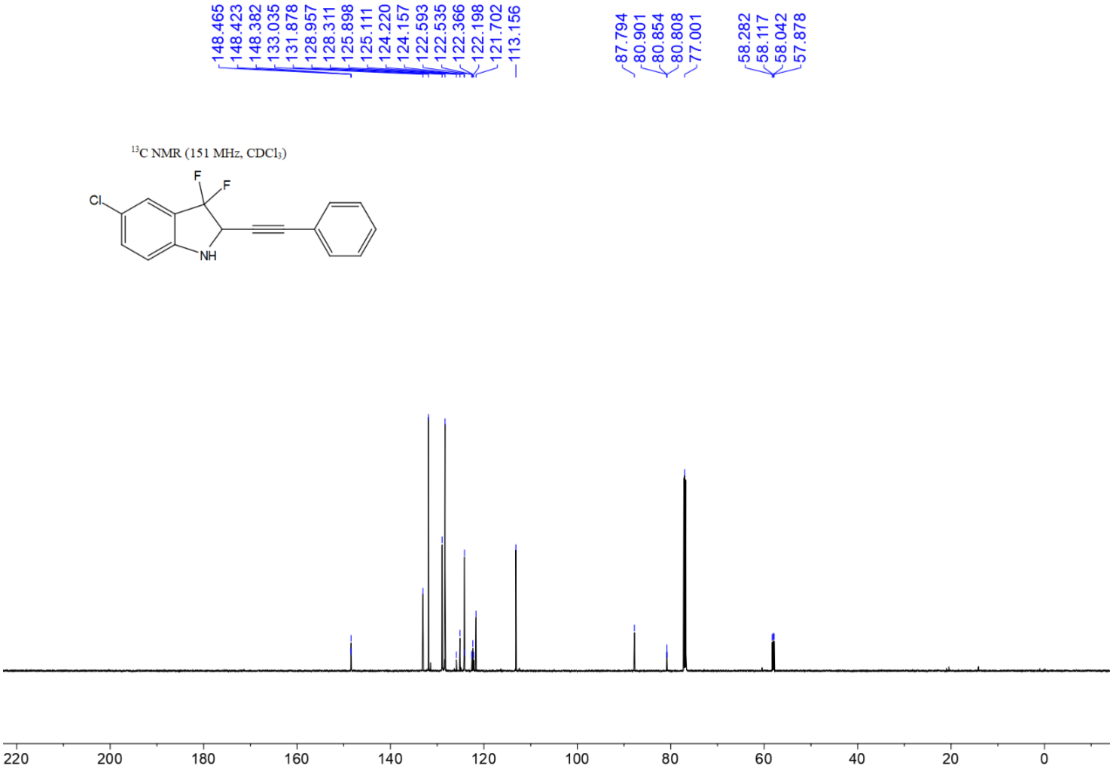

^{19}F NMR spectra of **2b**

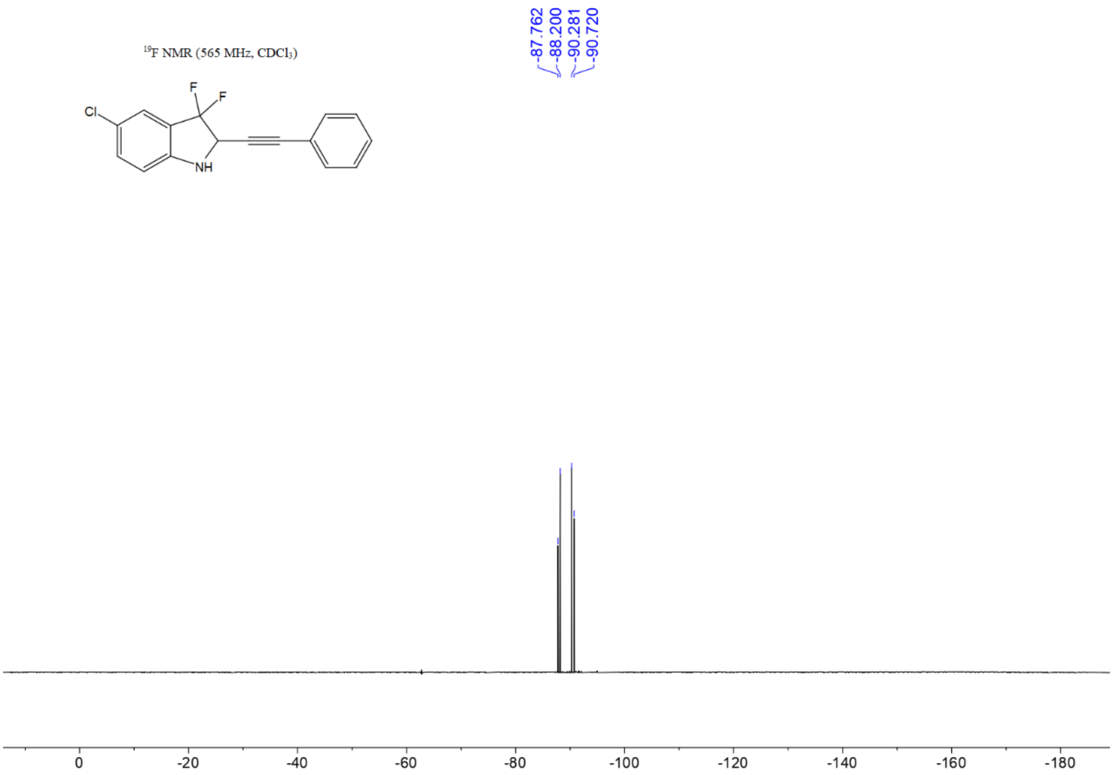

^{1}H NMR spectra of **2c**

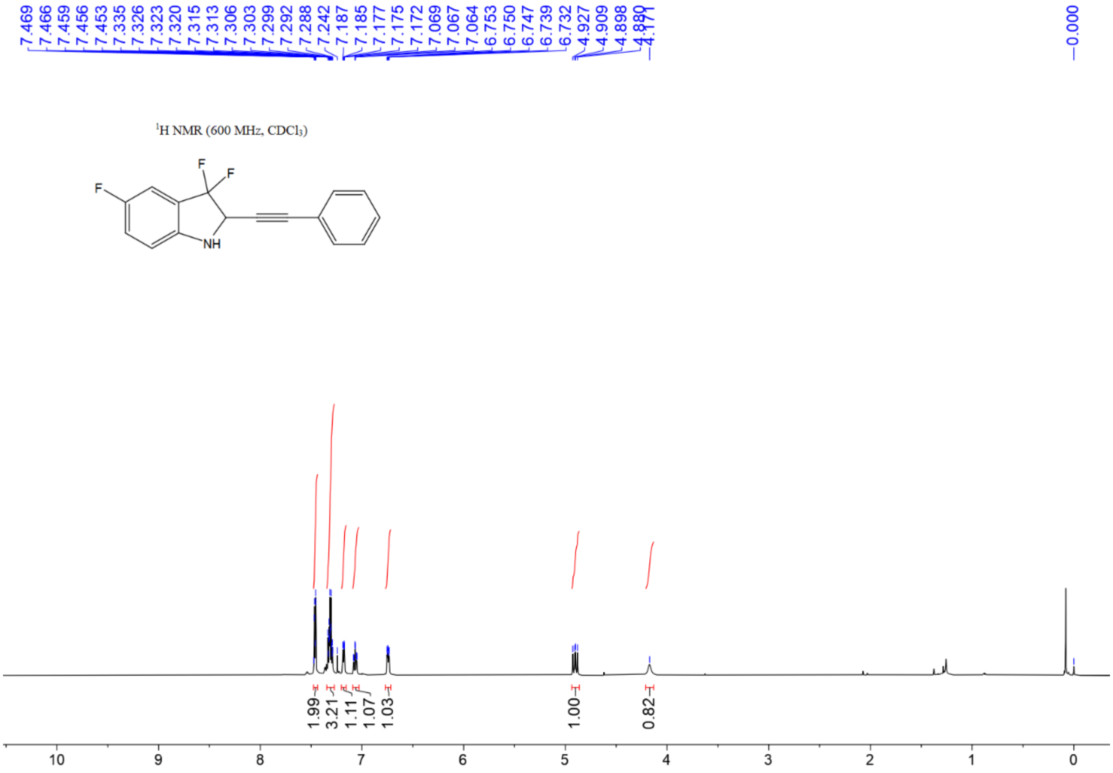

^{13}C NMR spectra of **2c**

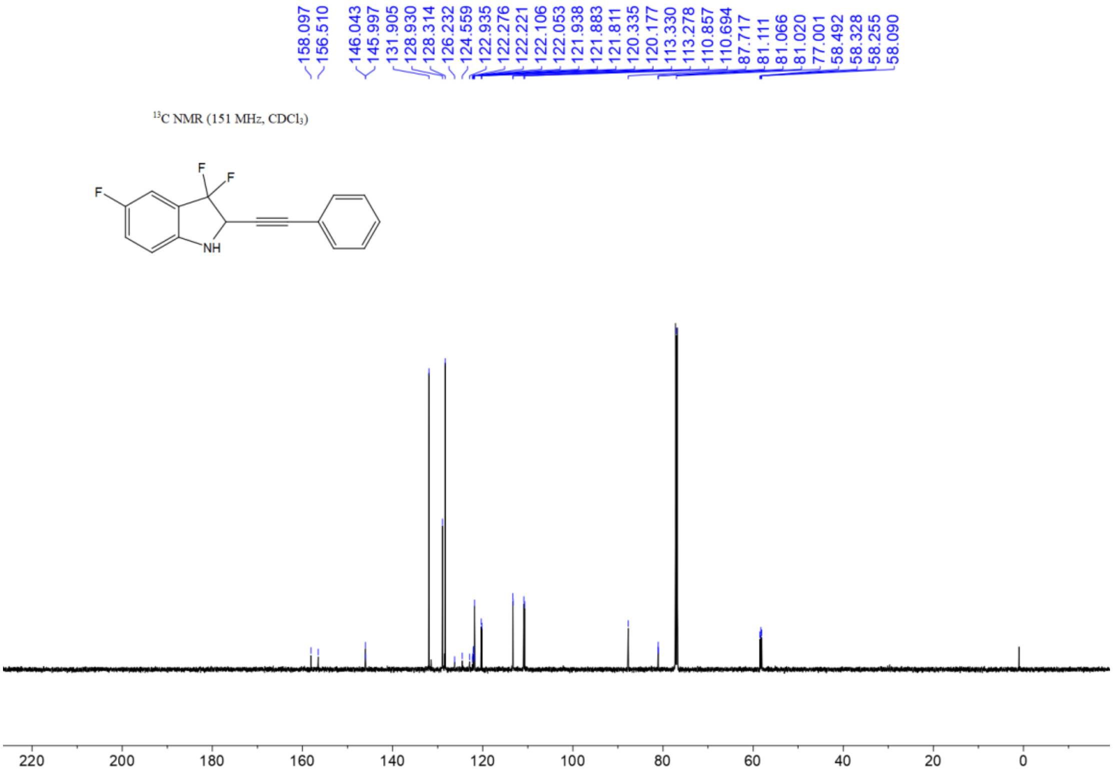

^{19}F NMR spectra of **2c**

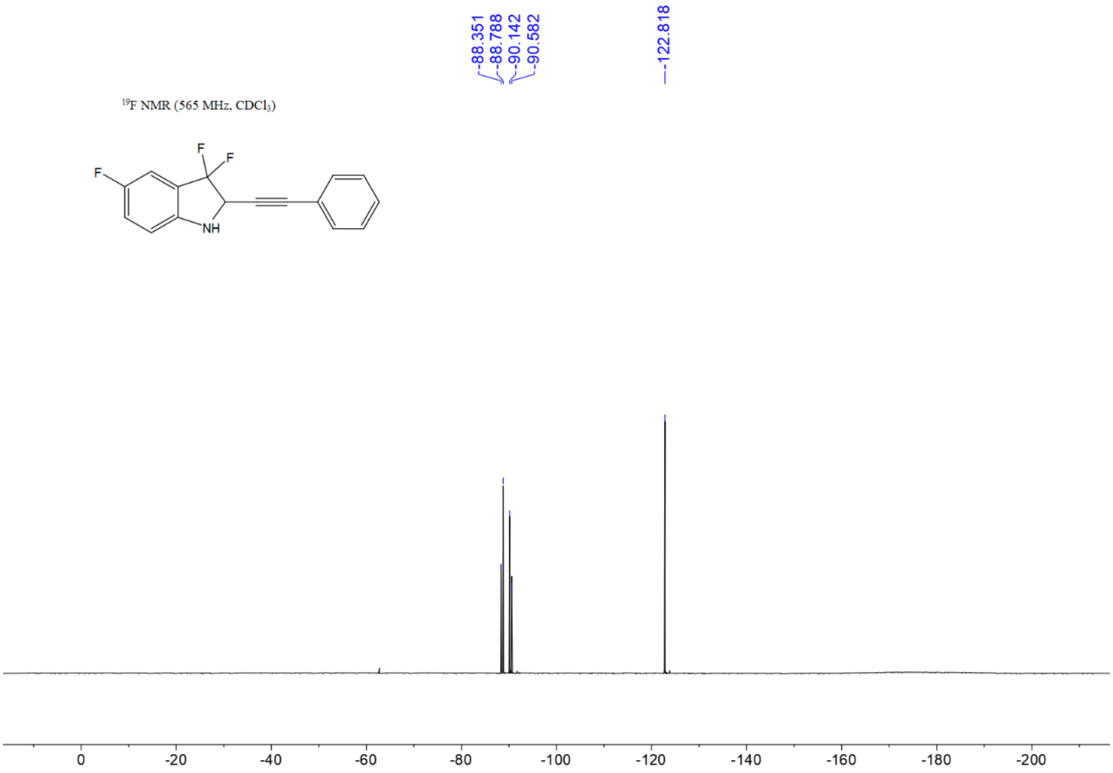

^{1}H NMR spectra of **2d**

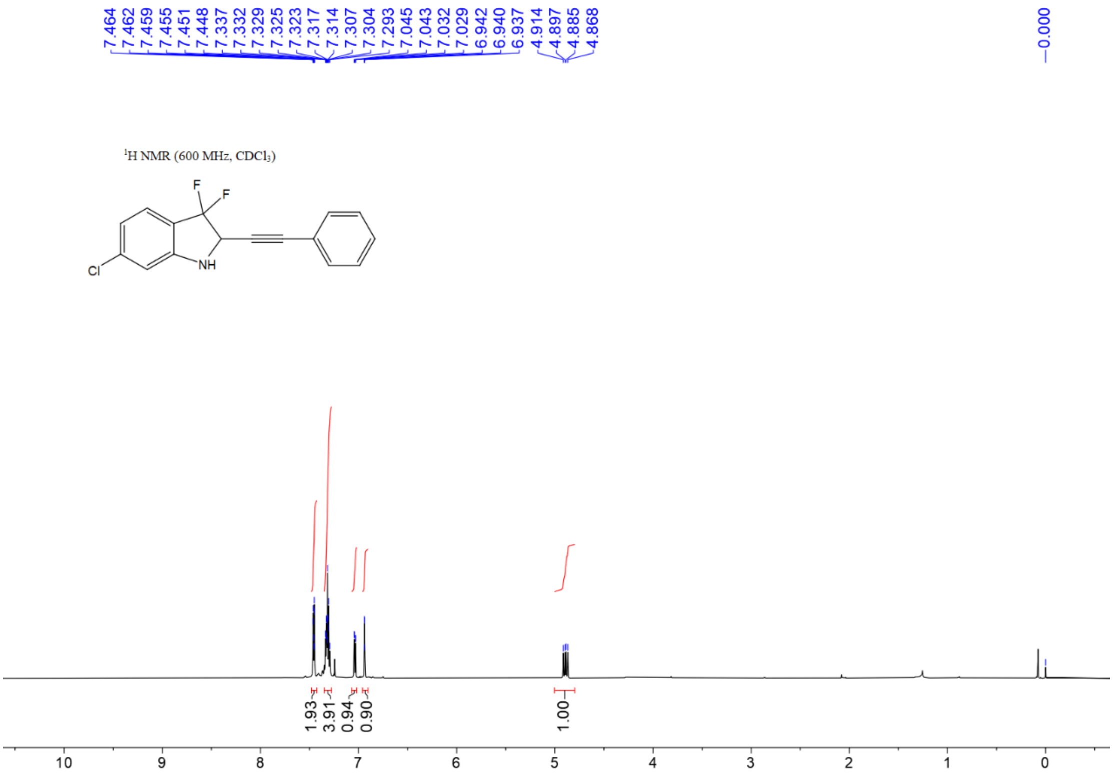

^{13}C NMR spectra of **2d**

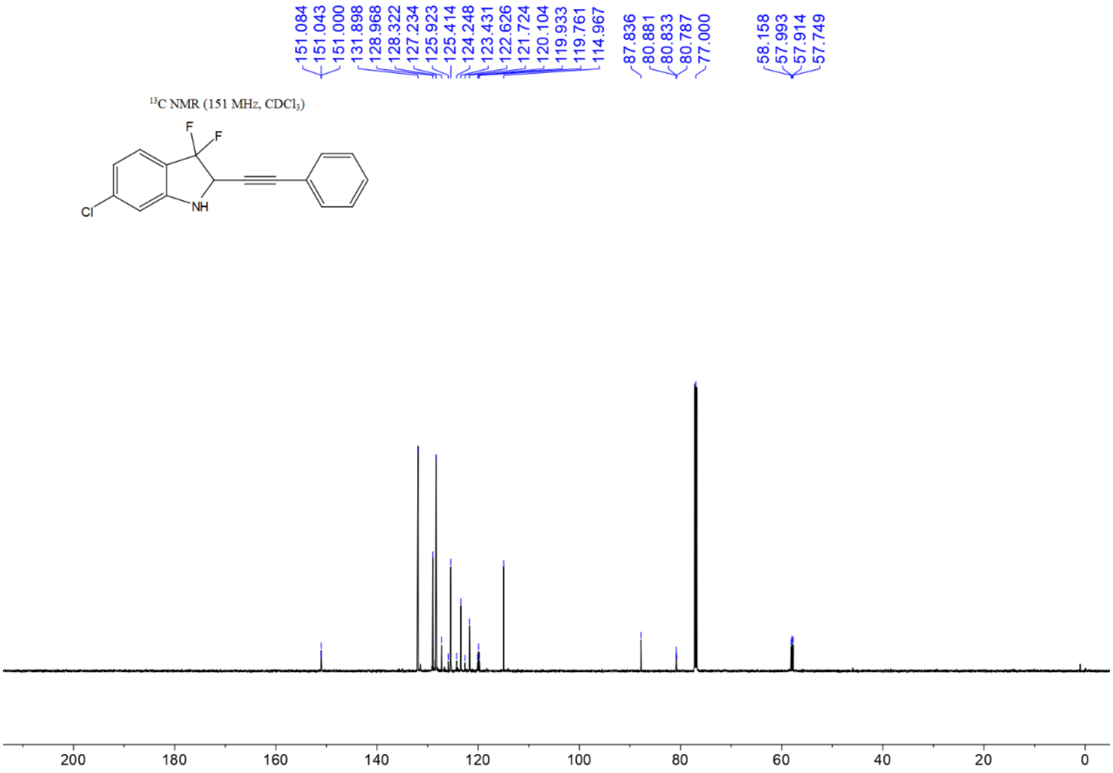

^{19}F NMR spectra of **2d**

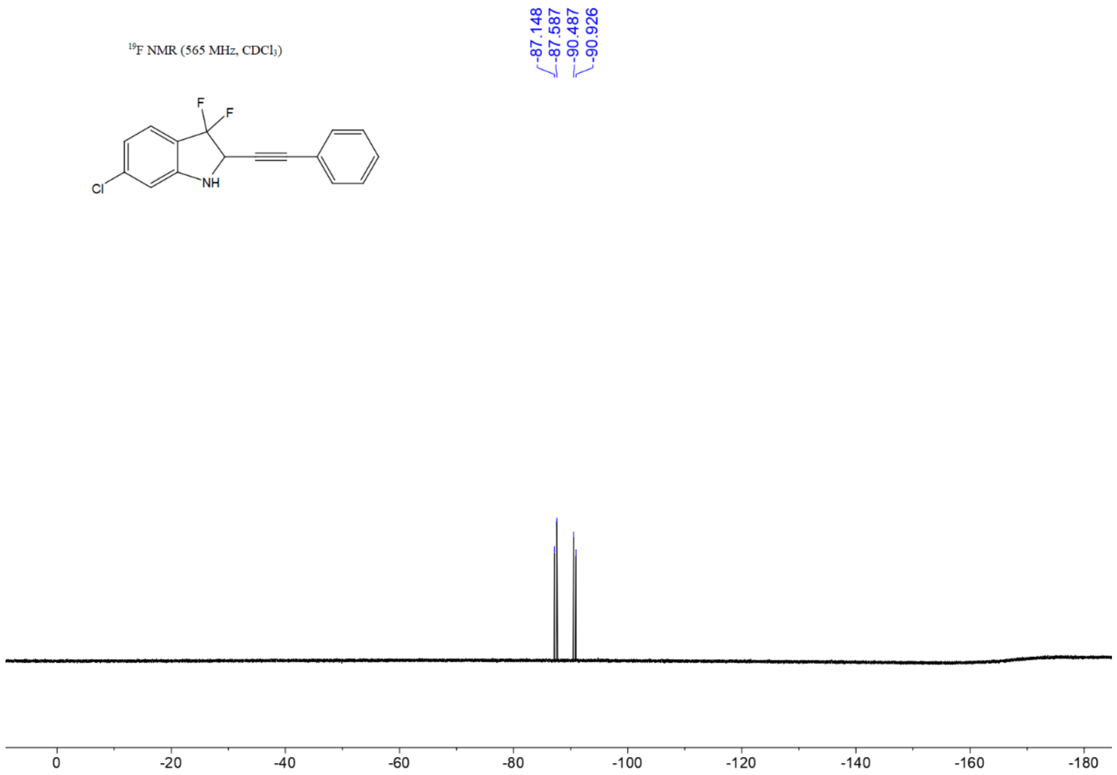

^{1}H NMR spectra of **2e**

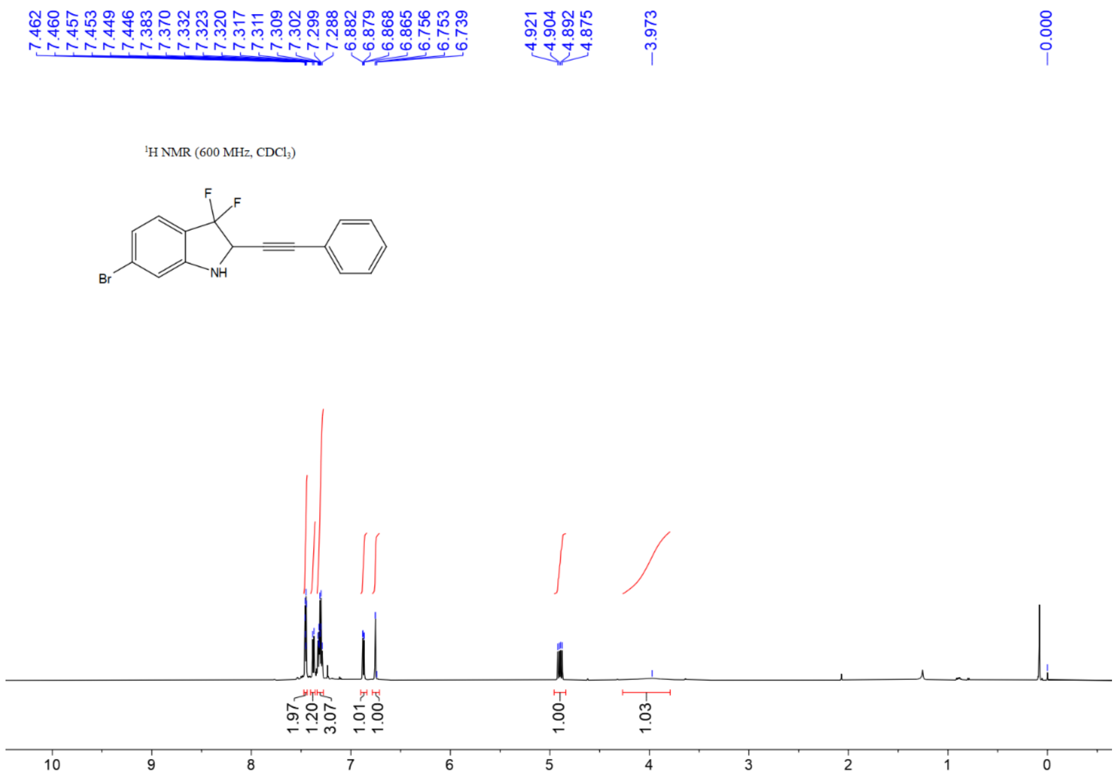

^{13}C NMR spectra of **2e**

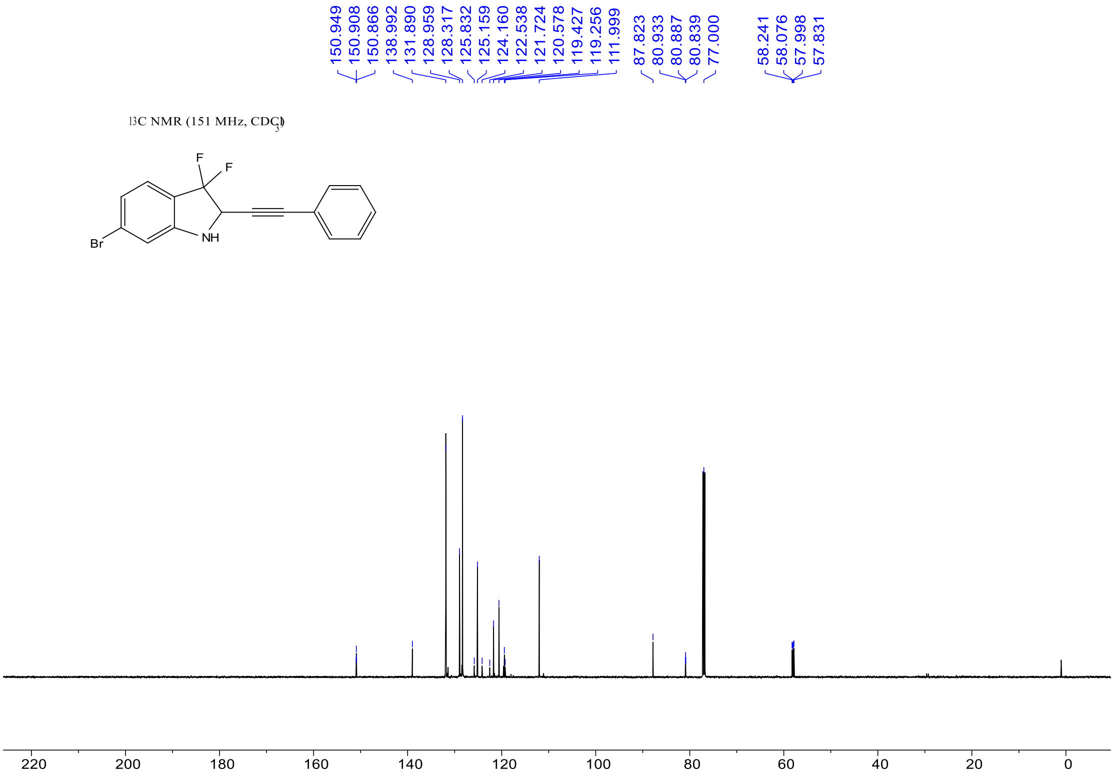

^{19}F NMR spectra of **2e**

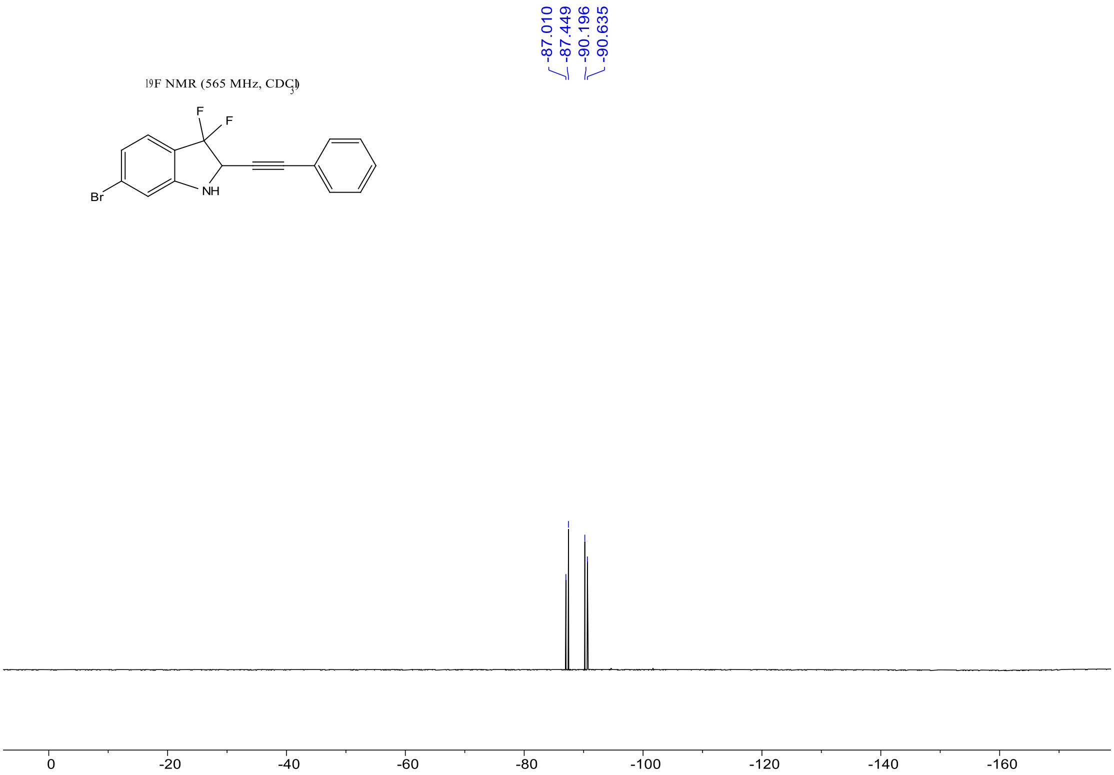

^{1}H NMR spectra of **2f**

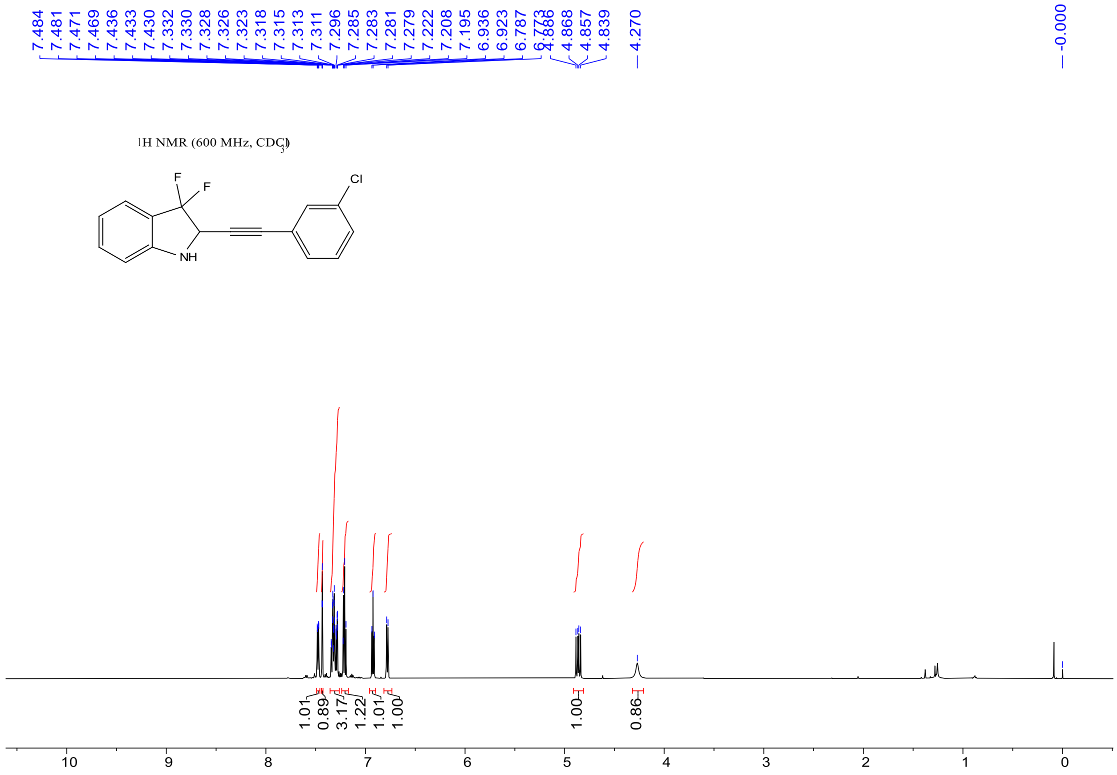

^{13}C NMR spectra of **2f**

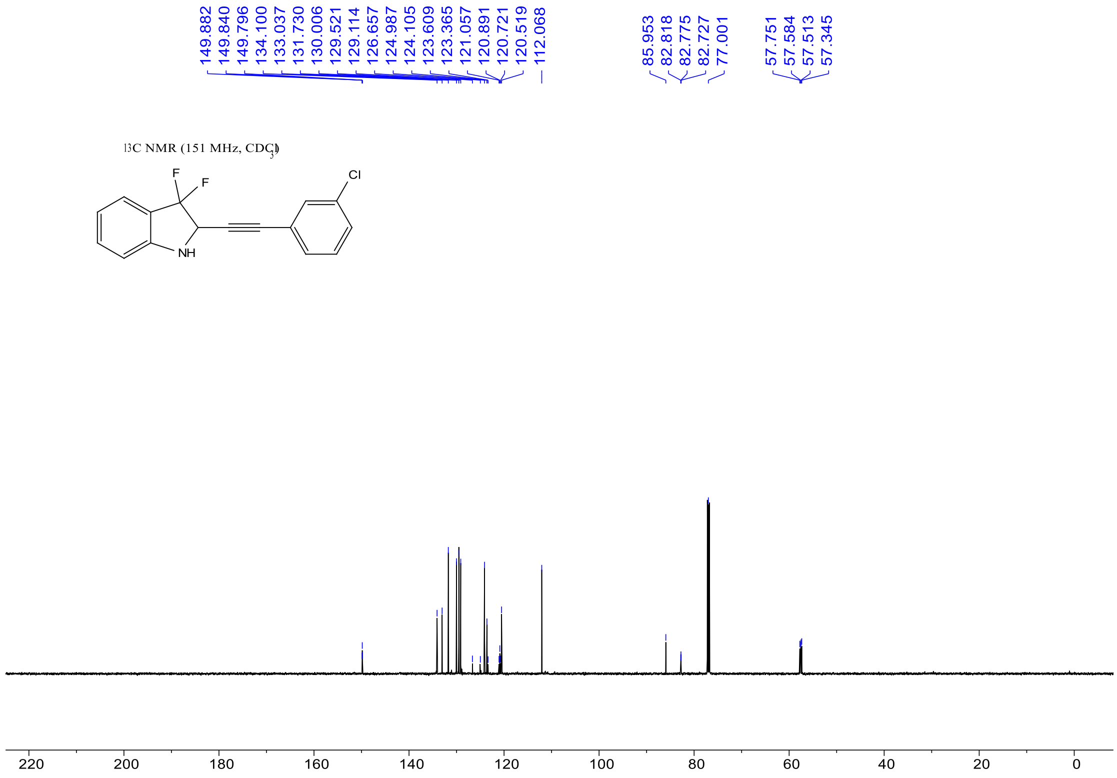

^{19}F NMR spectra of **2f**

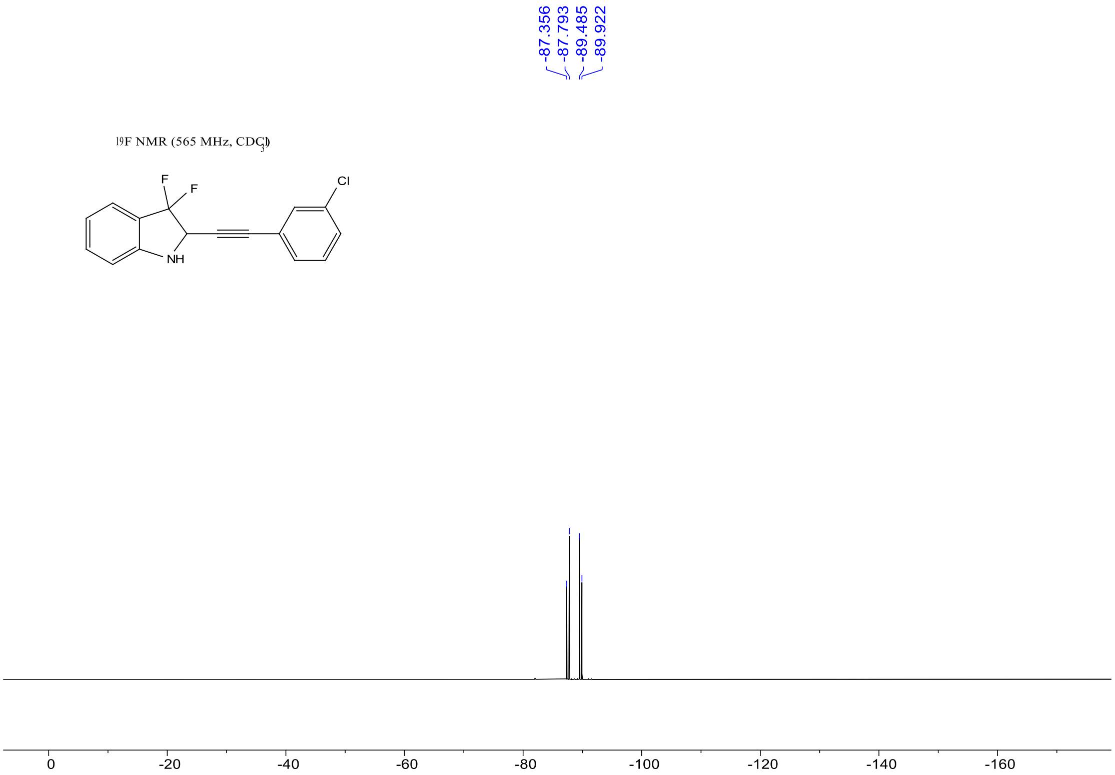

^{1}H NMR spectra of **2g**

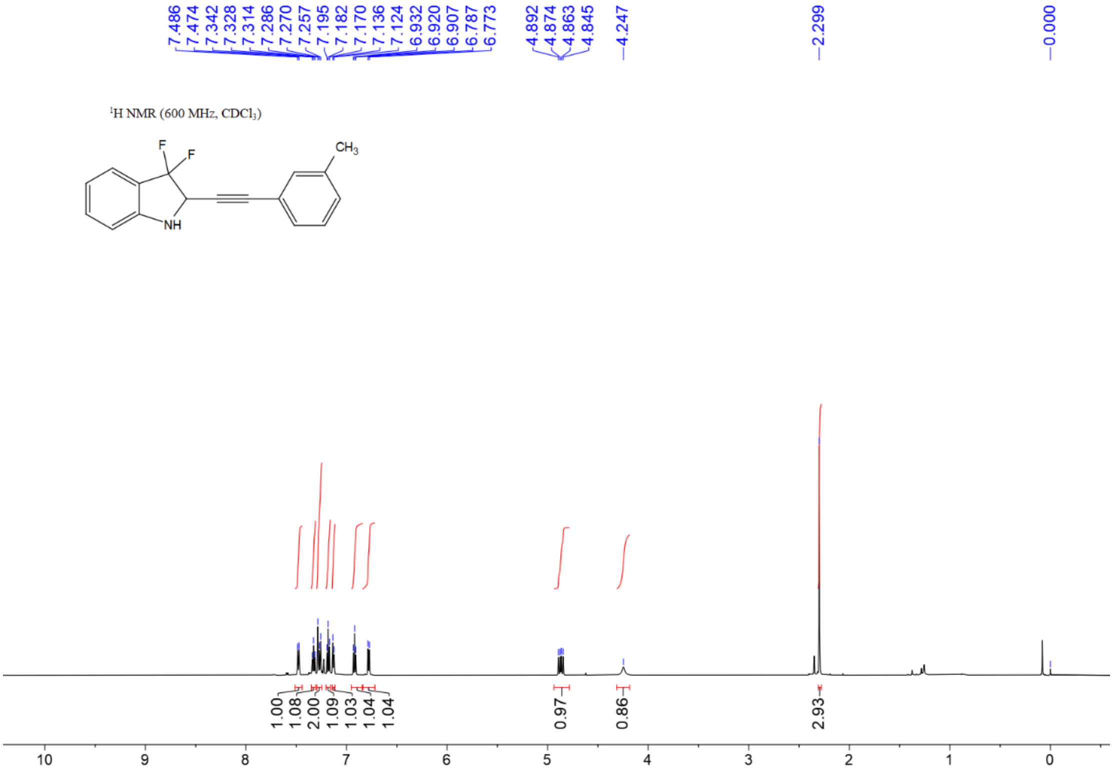

^{13}C NMR spectra of **2g**

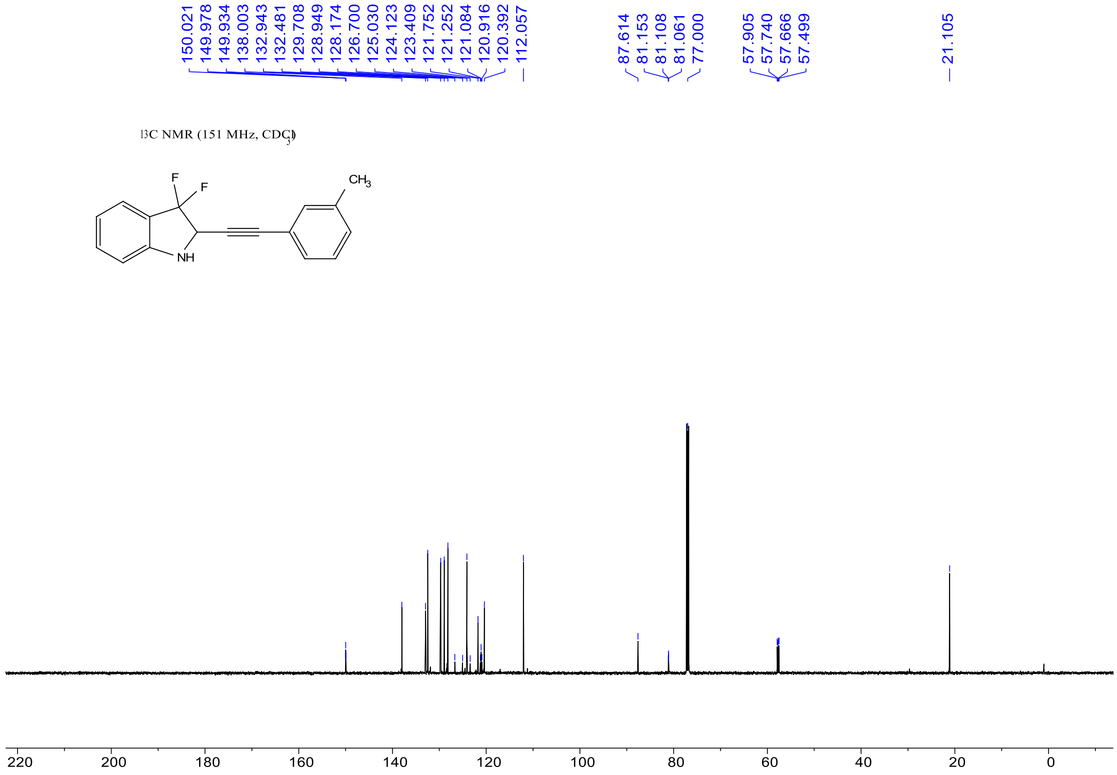

^{19}F NMR spectra of **2g**

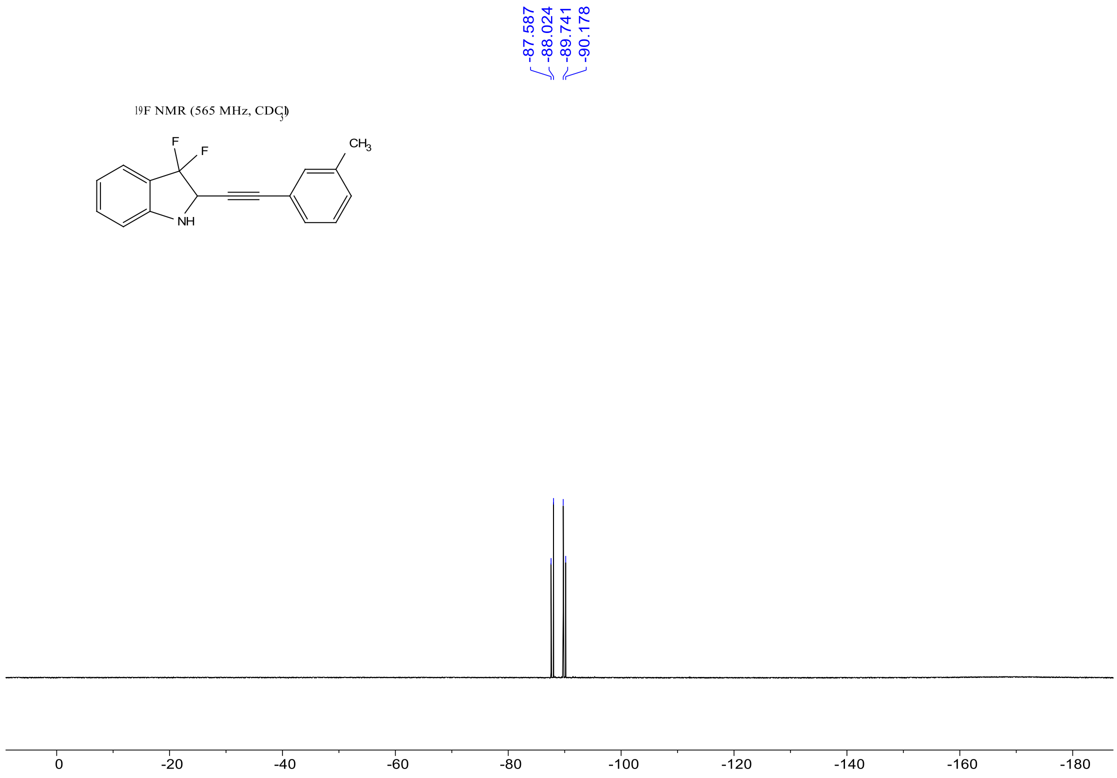

^{1}H NMR spectra of **2h**

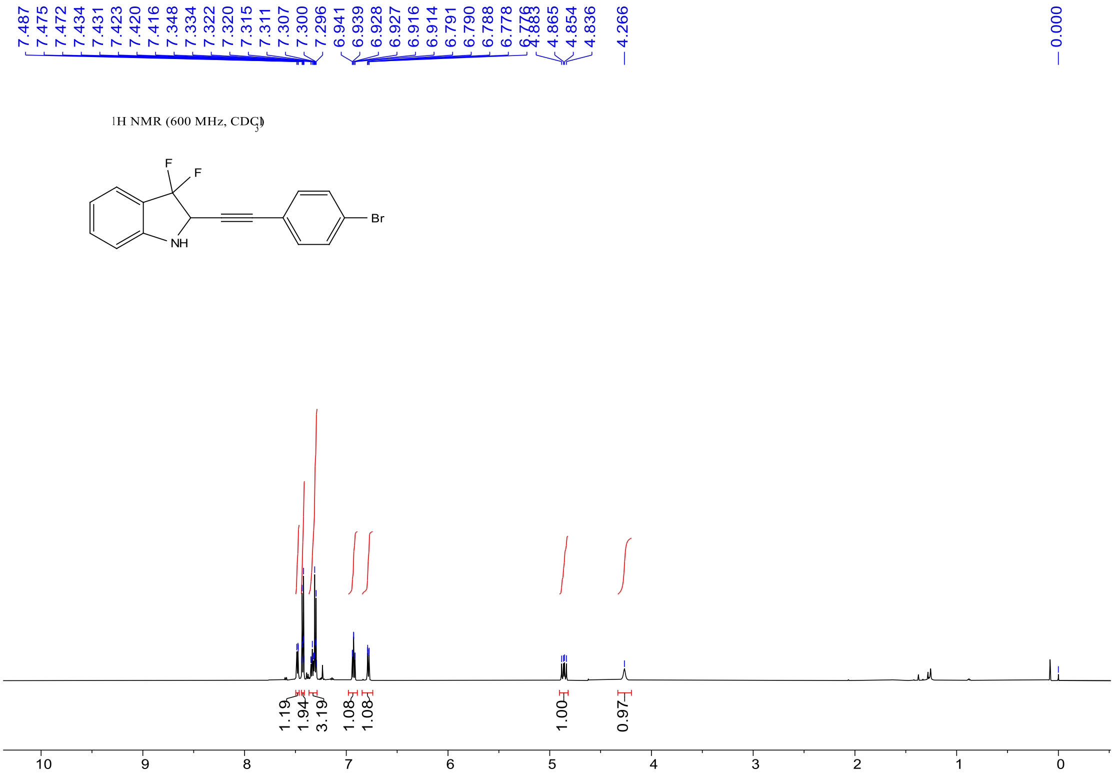

^{13}C NMR spectra of **2h**

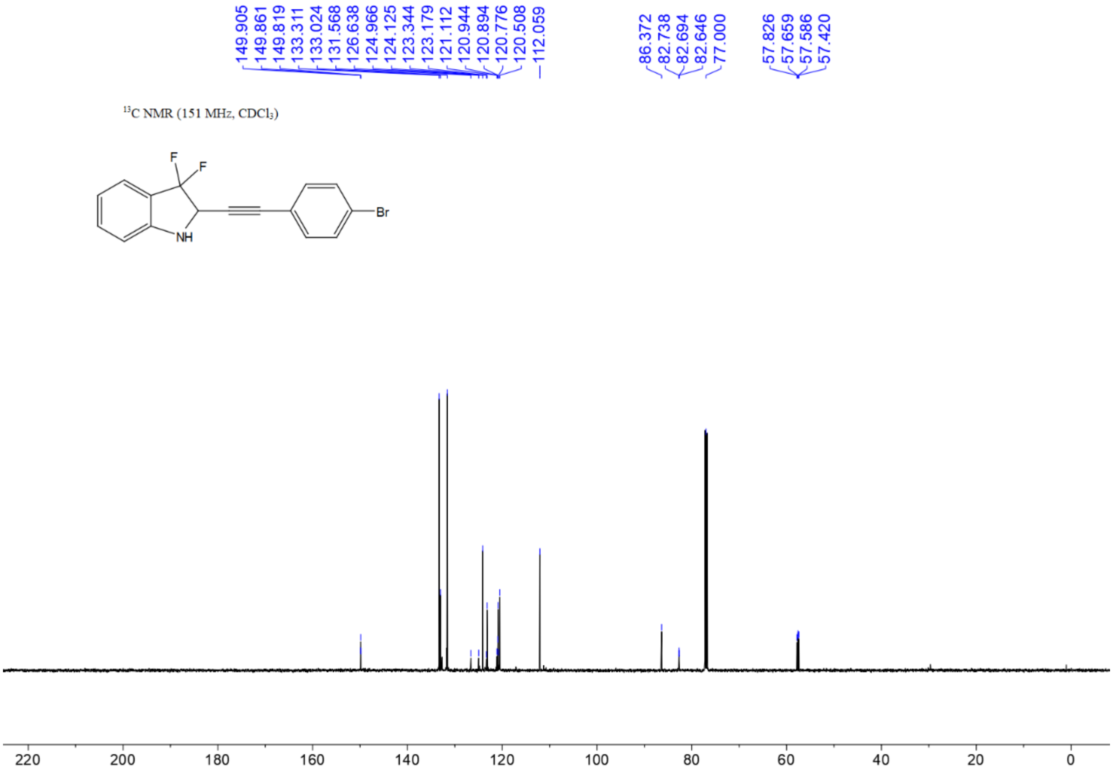

^{19}F NMR spectra of **2h**

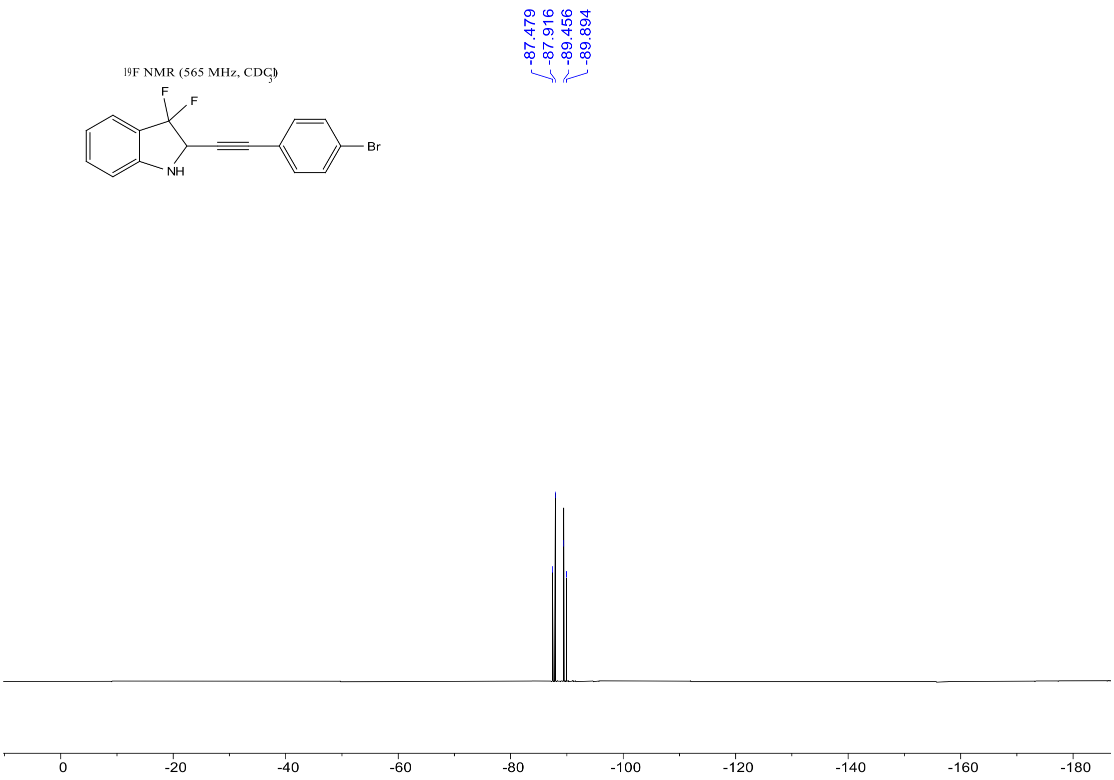

^{1}H NMR spectra of **2i**

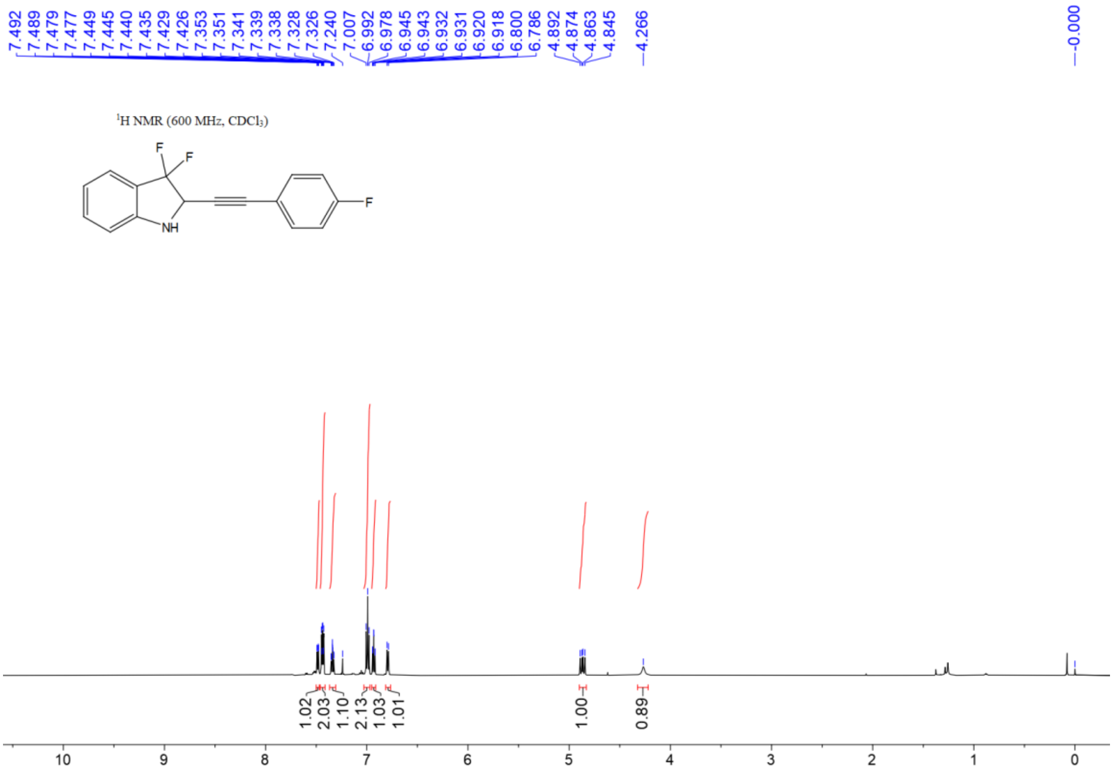

^{13}C NMR spectra of **2i**

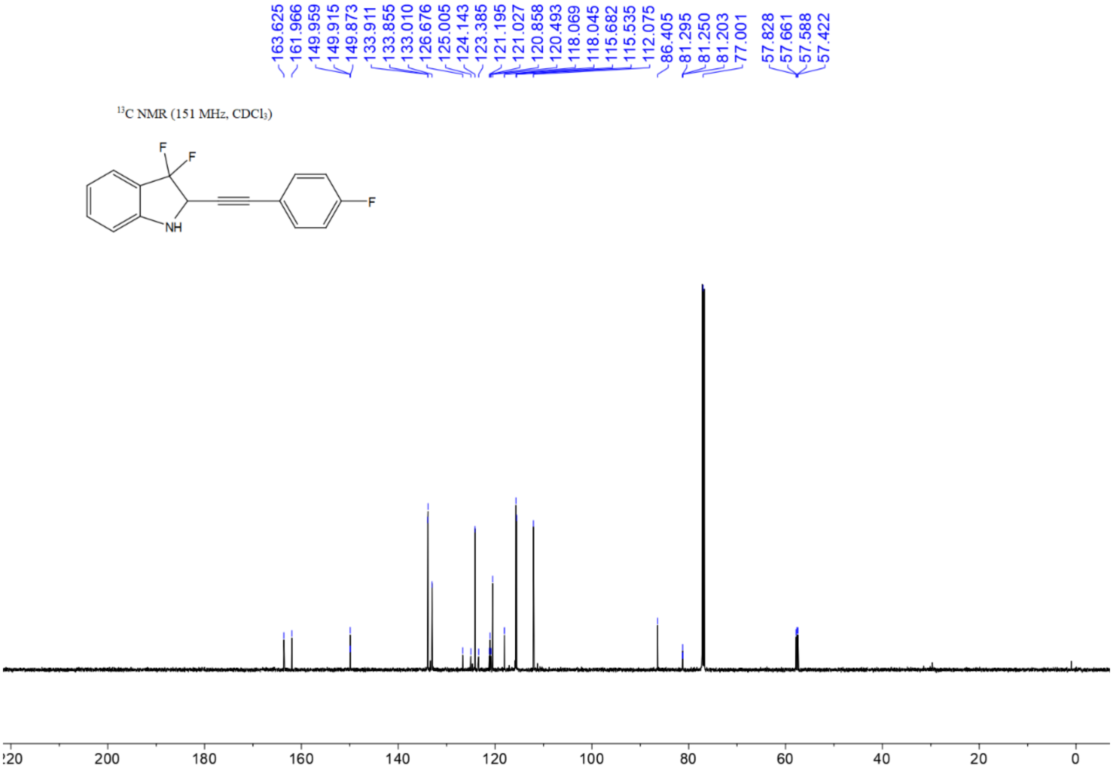

^{19}F NMR spectra of **2i**

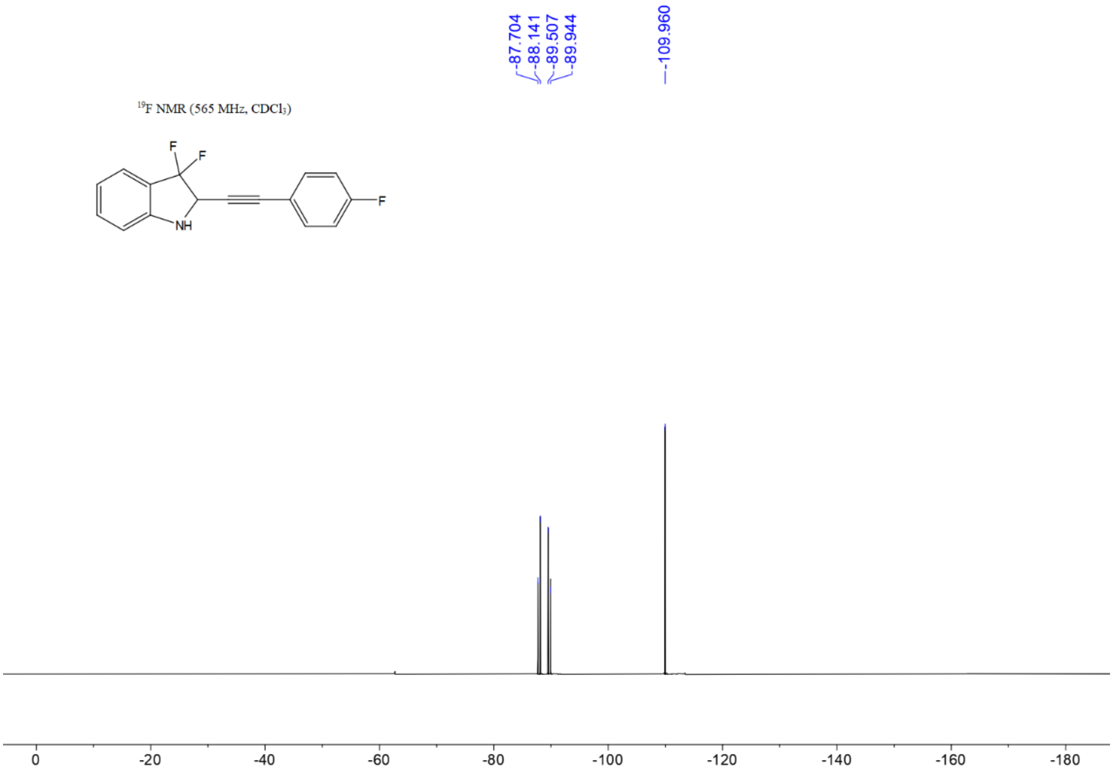

^{1}H NMR spectra of **2j**

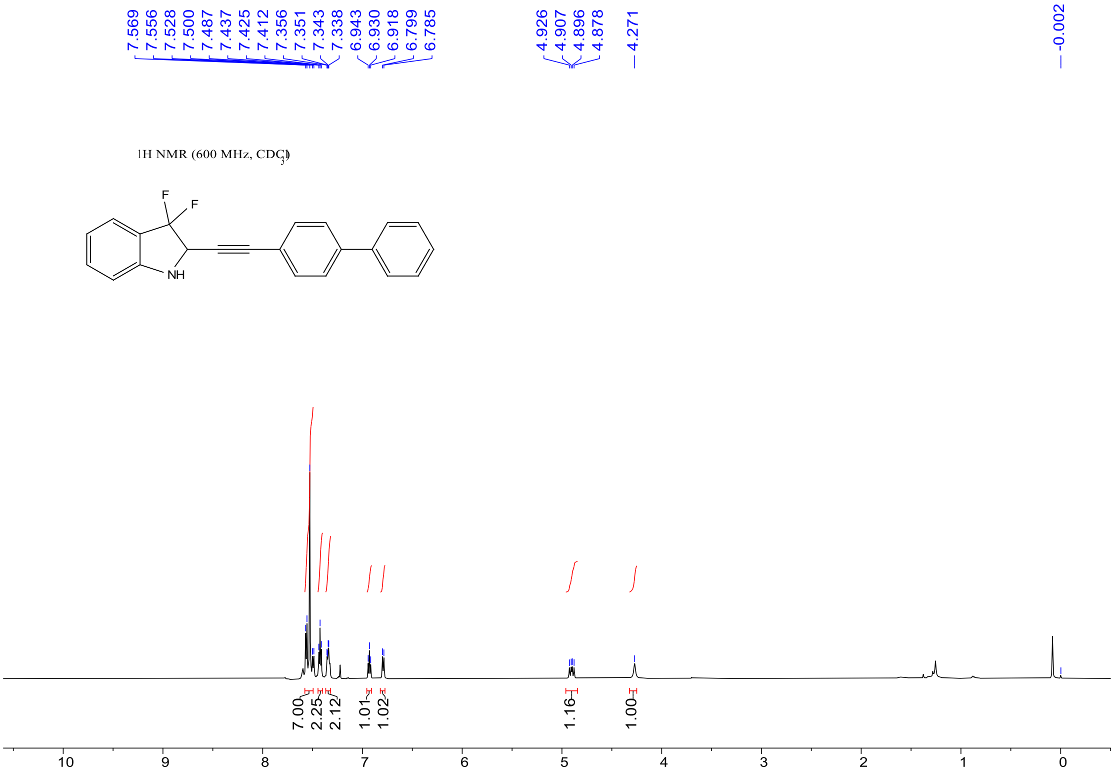

^{13}C NMR spectra of **2j**

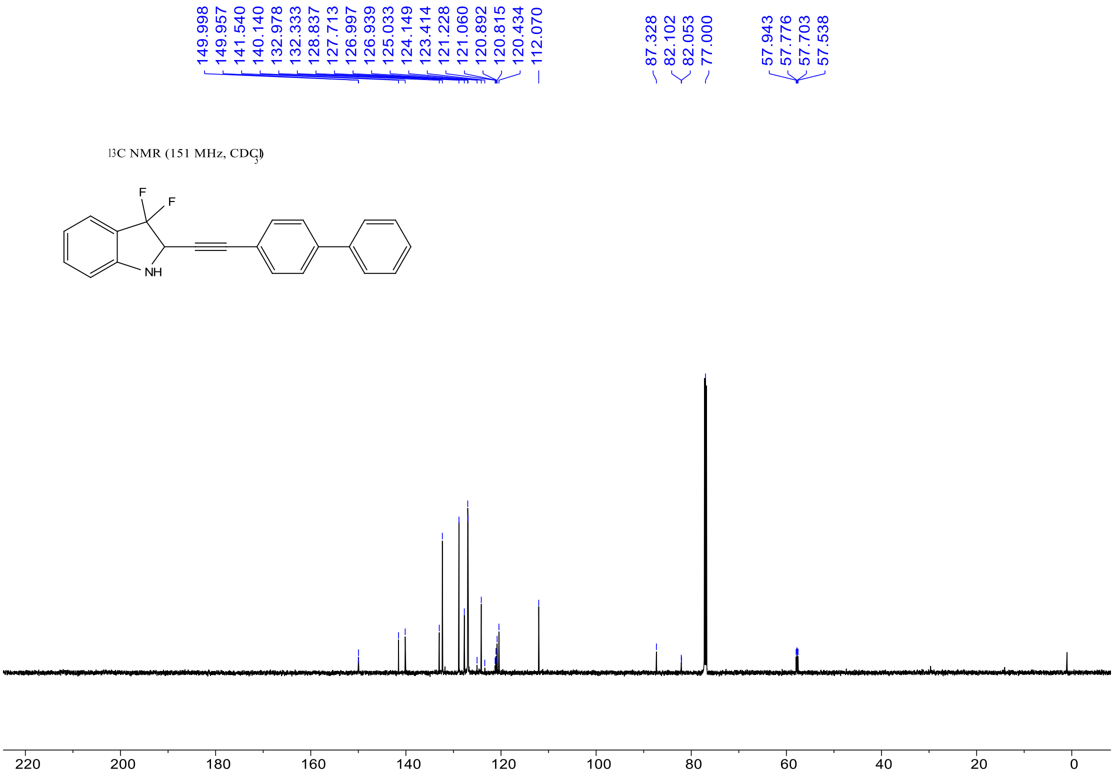

^{19}F NMR spectra of **2j**

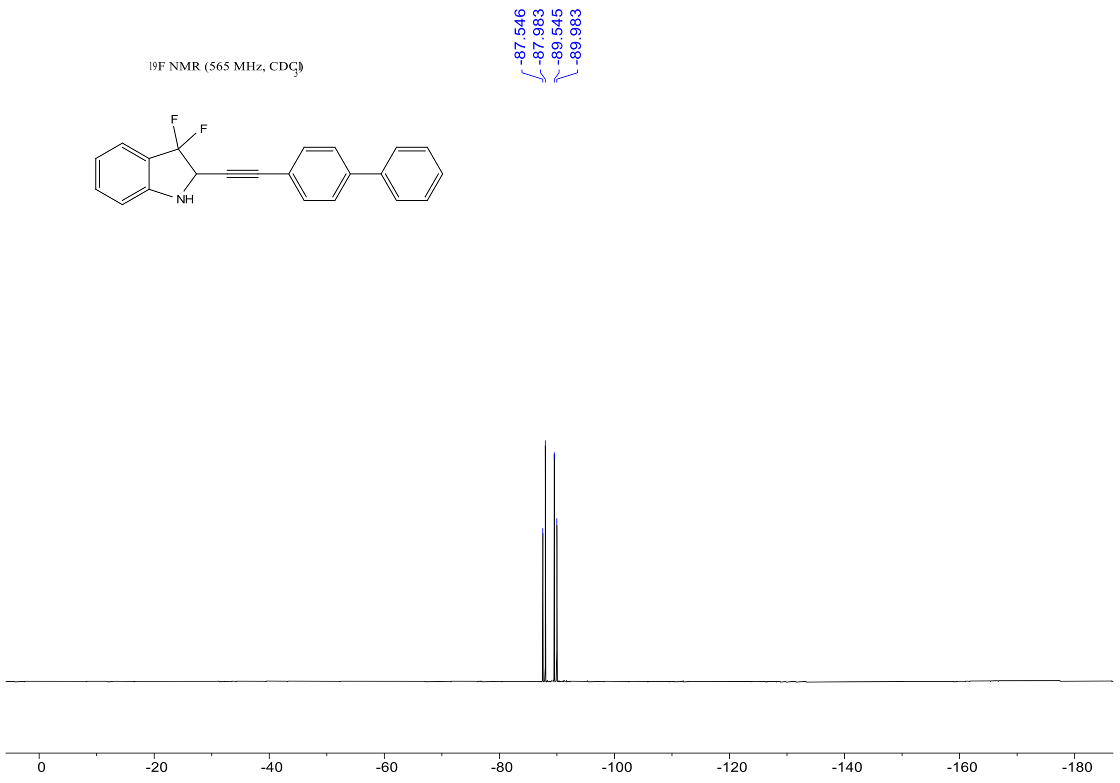

^{1}H NMR spectra of **2k**

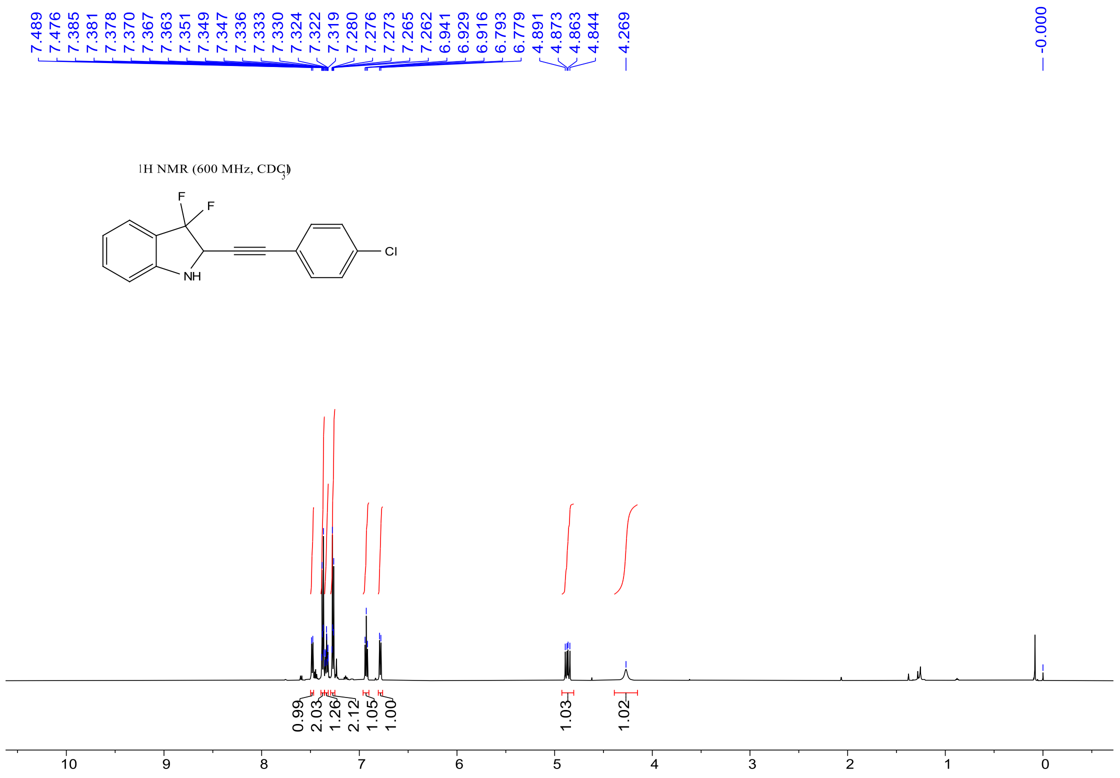

^{13}C NMR spectra of **2k**

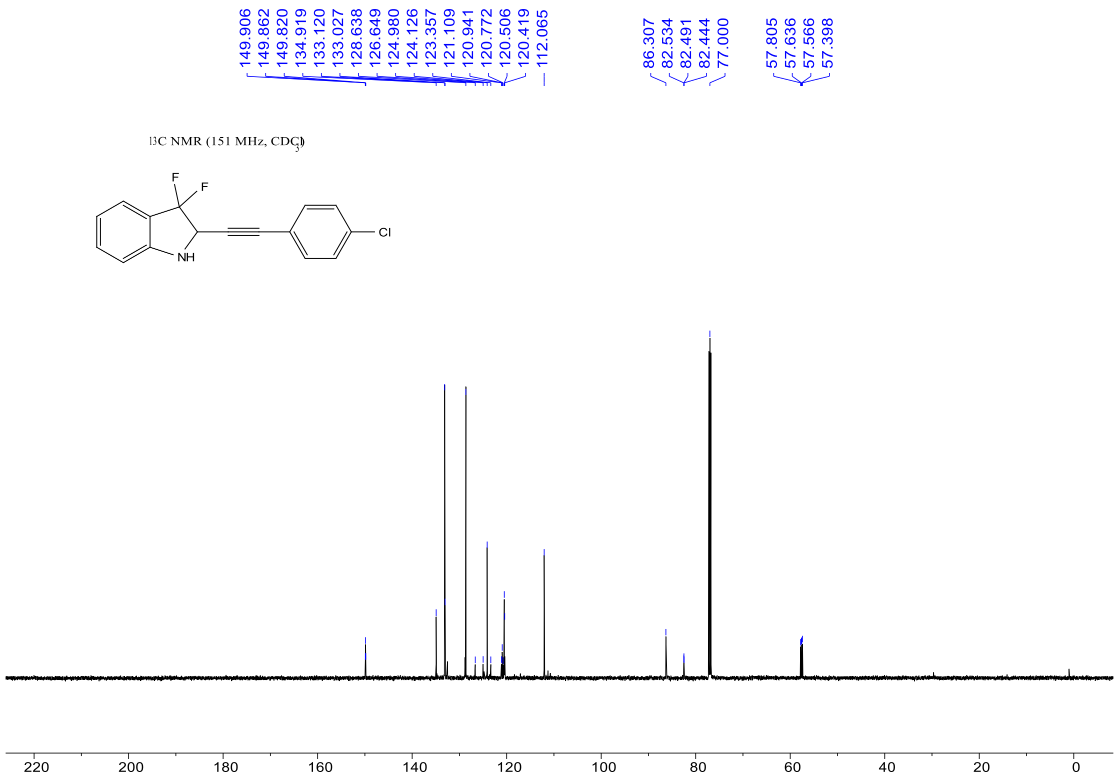

^{19}F NMR spectra of **2k**

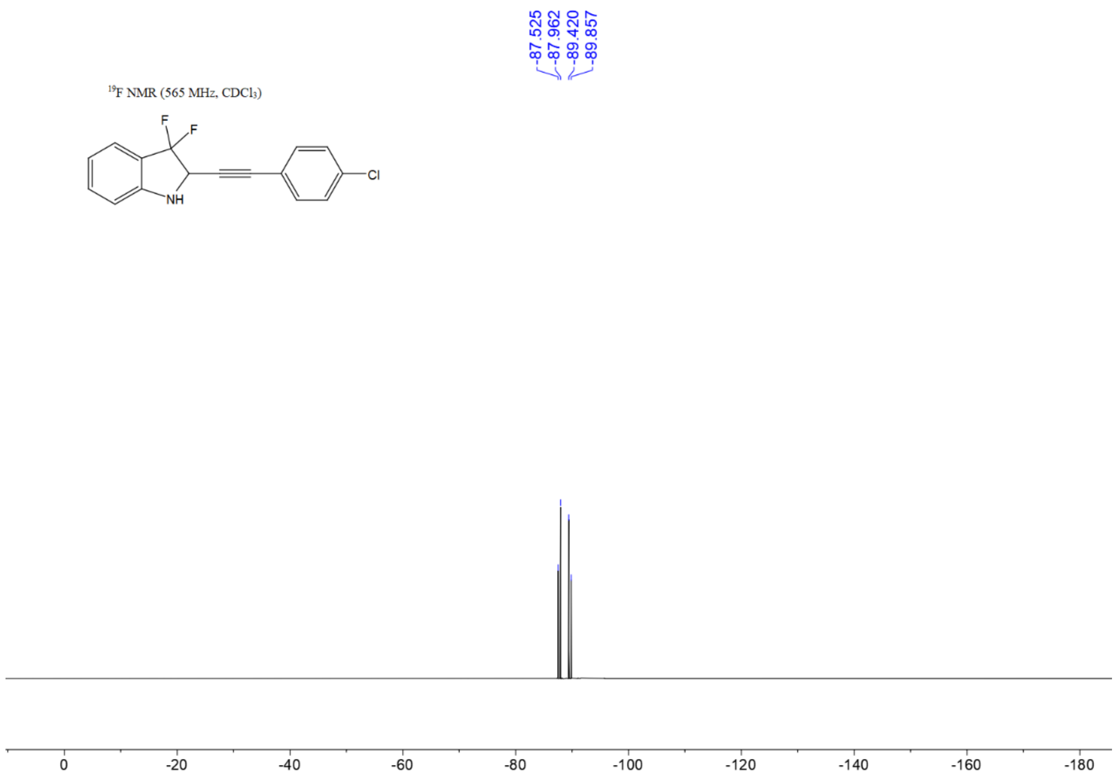

^{1}H NMR spectra of **2l**

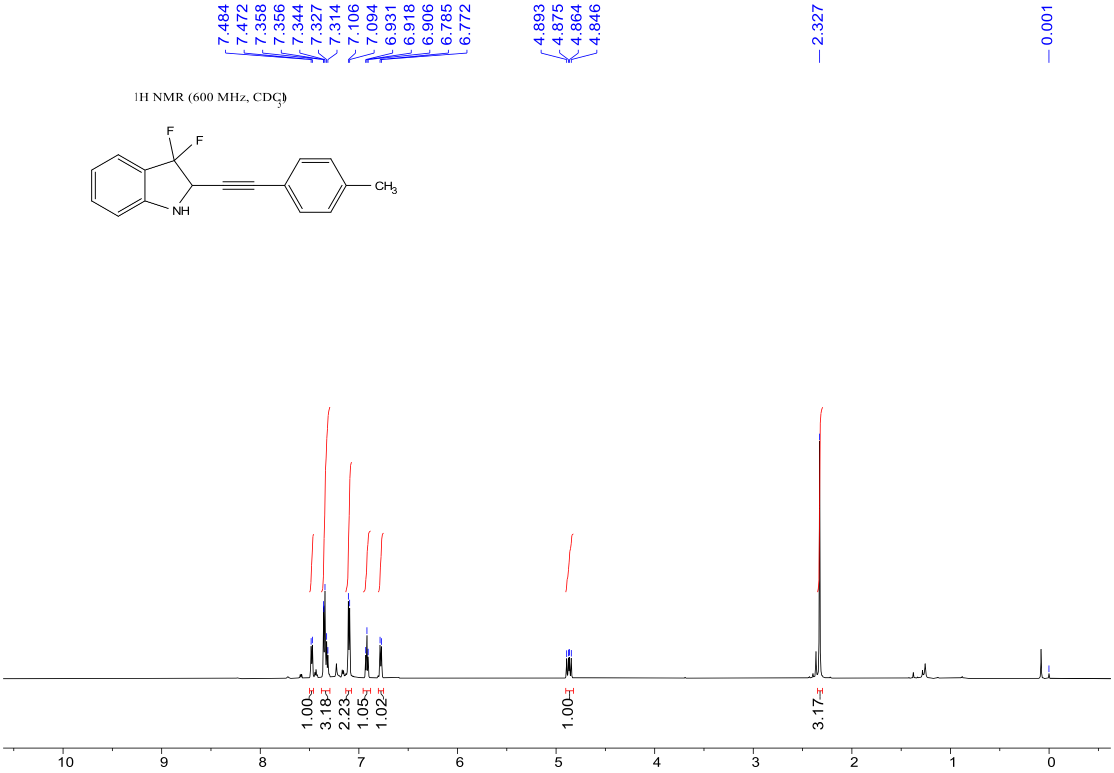

^{13}C NMR spectra of **2l**

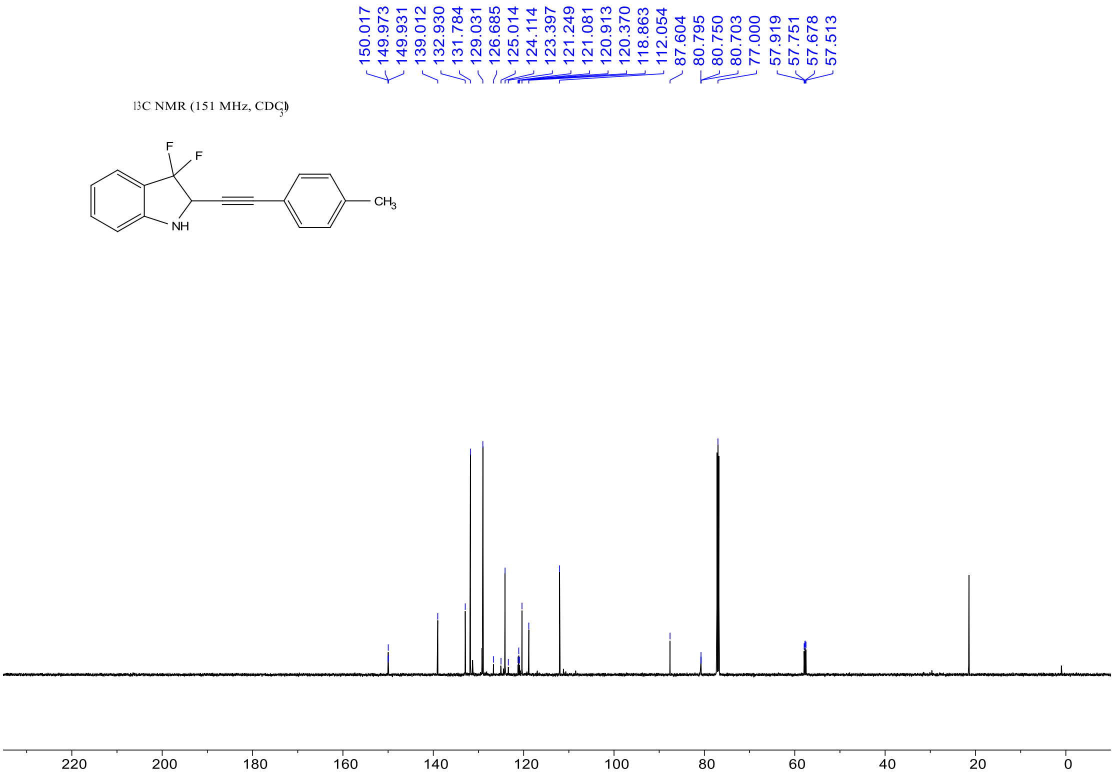

^{19}F NMR spectra of **2l**

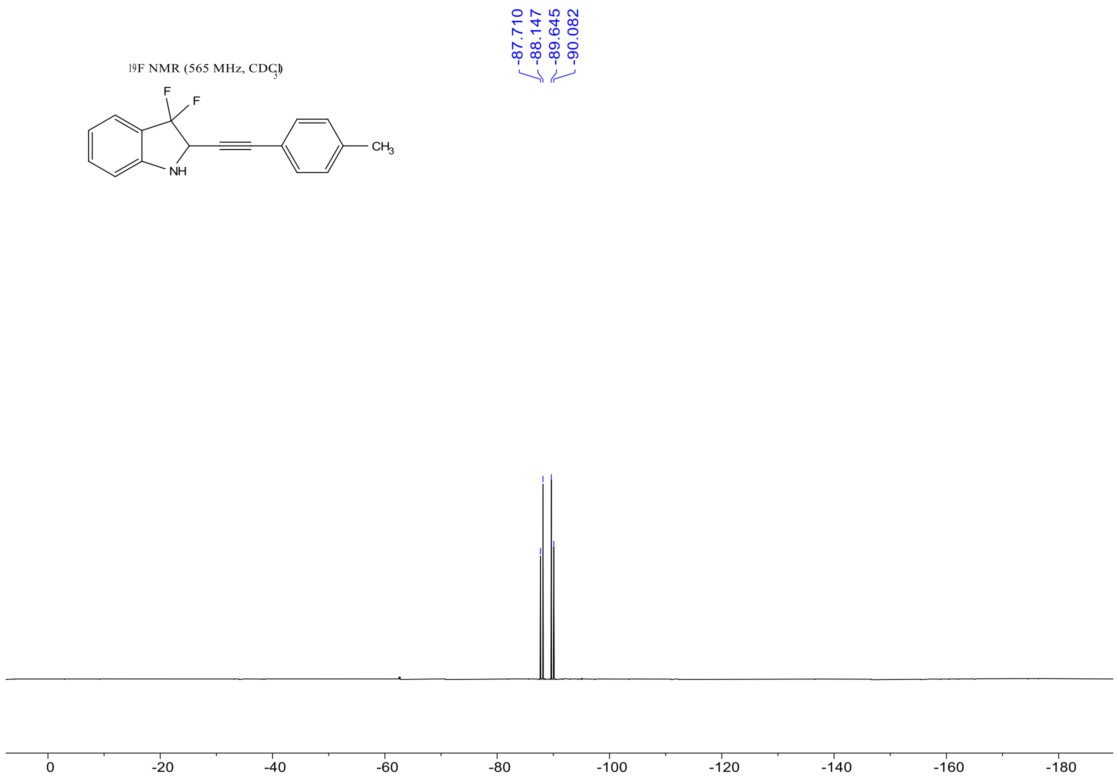

^{1}H NMR spectra of **2m**

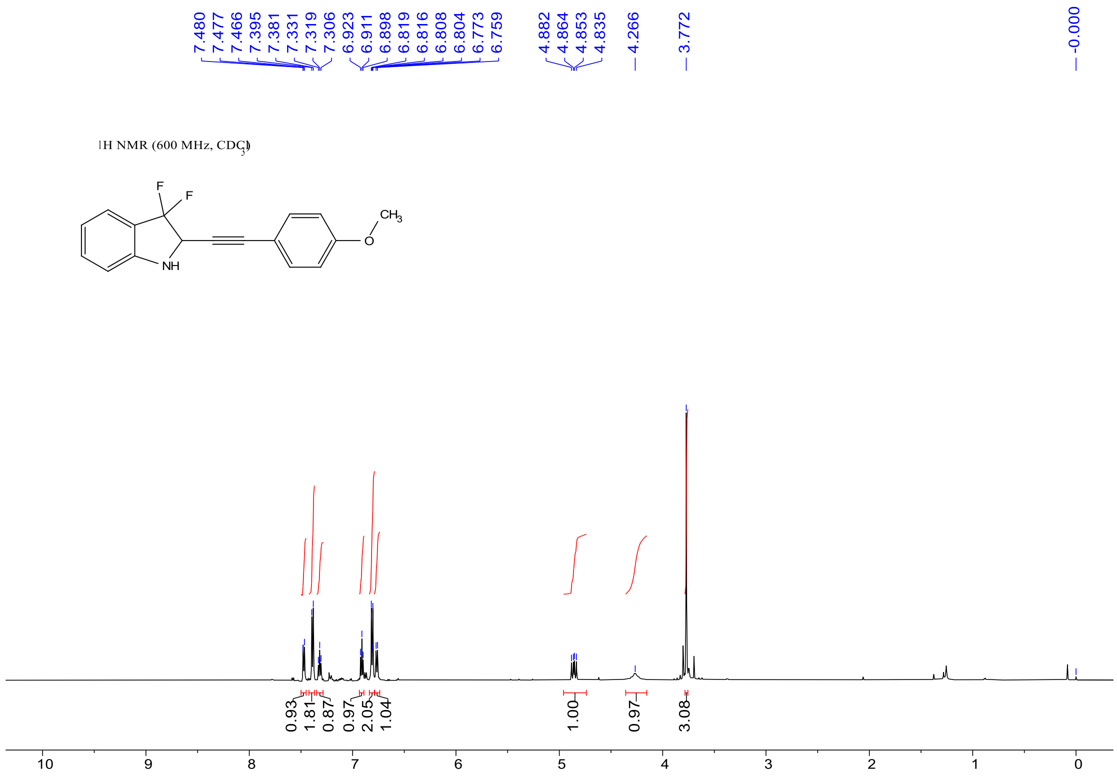

^{13}C NMR spectra of **2m**

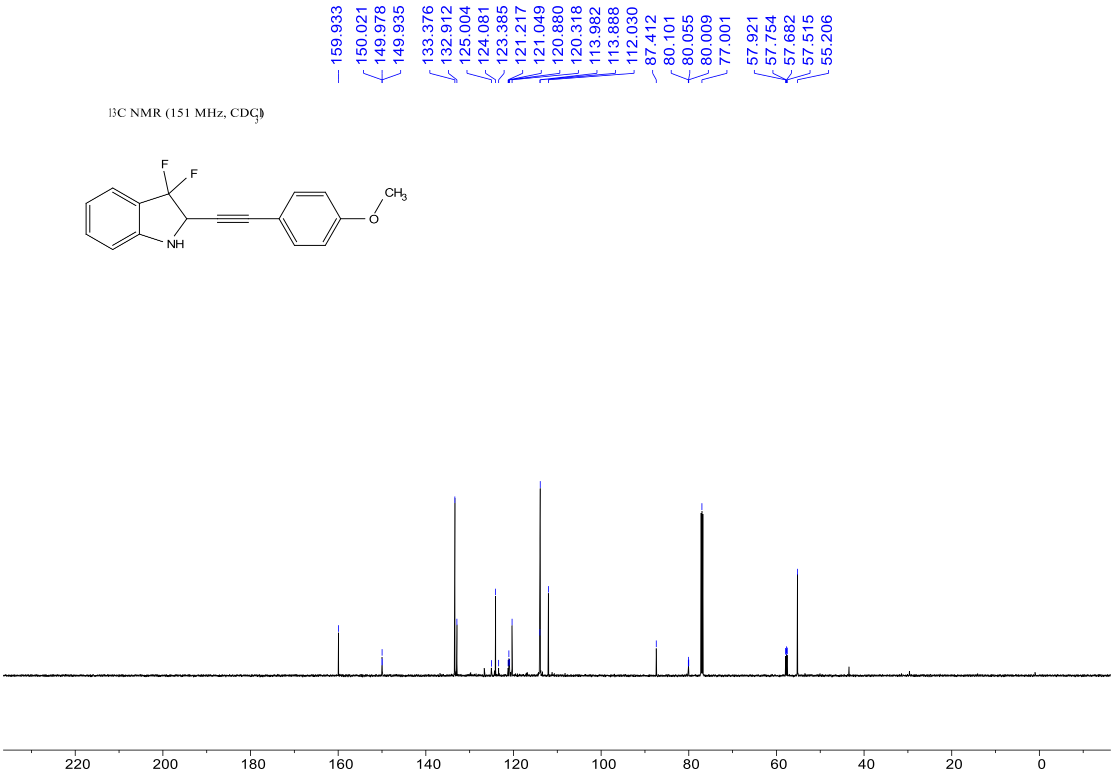

^{19}F NMR spectra of **2m**

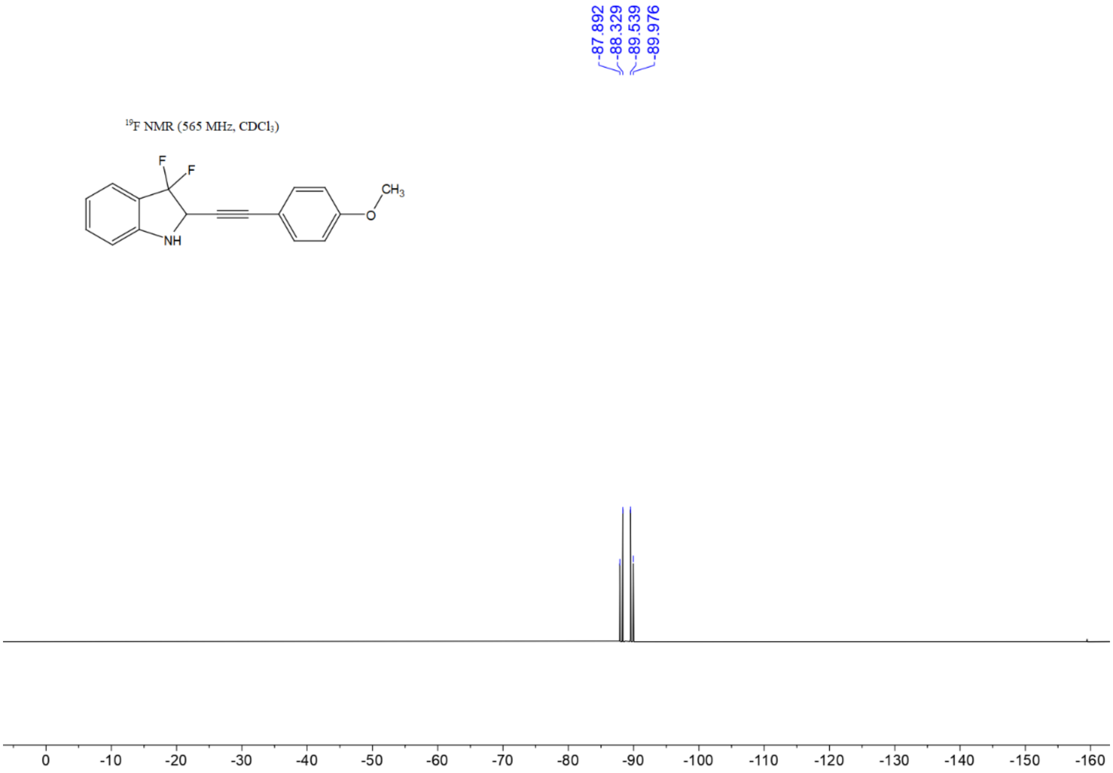

^{1}H NMR spectra of **2n**

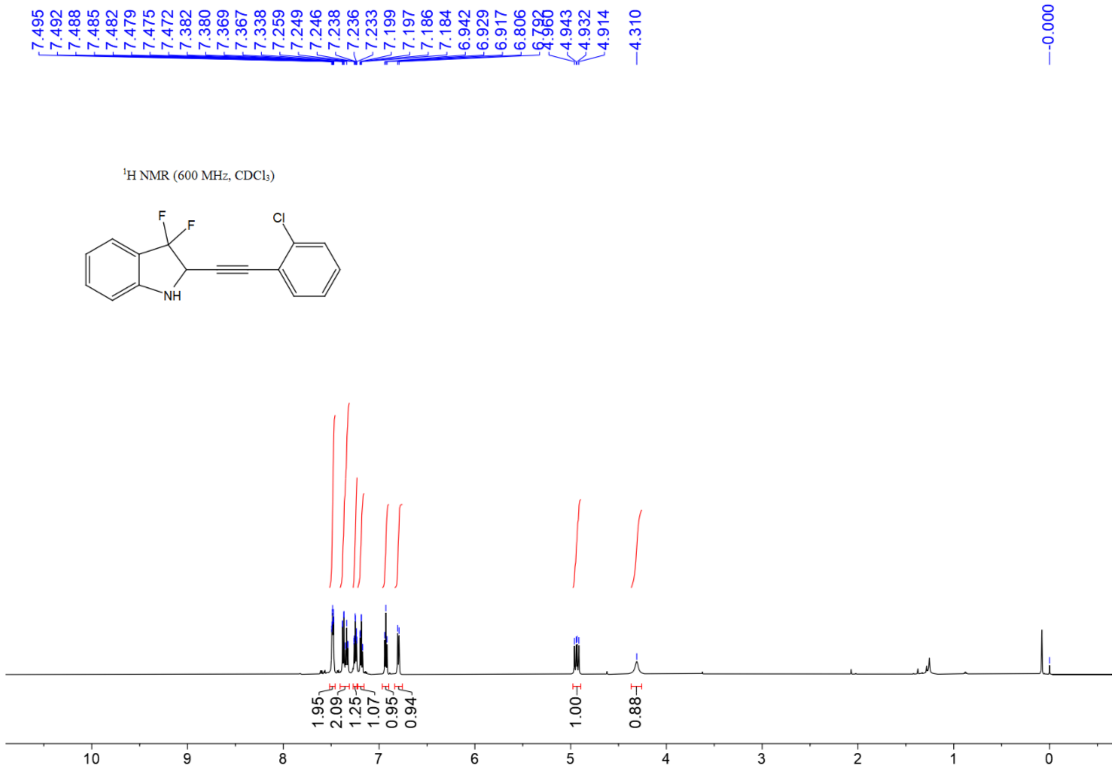

^{13}C NMR spectra of **2n**

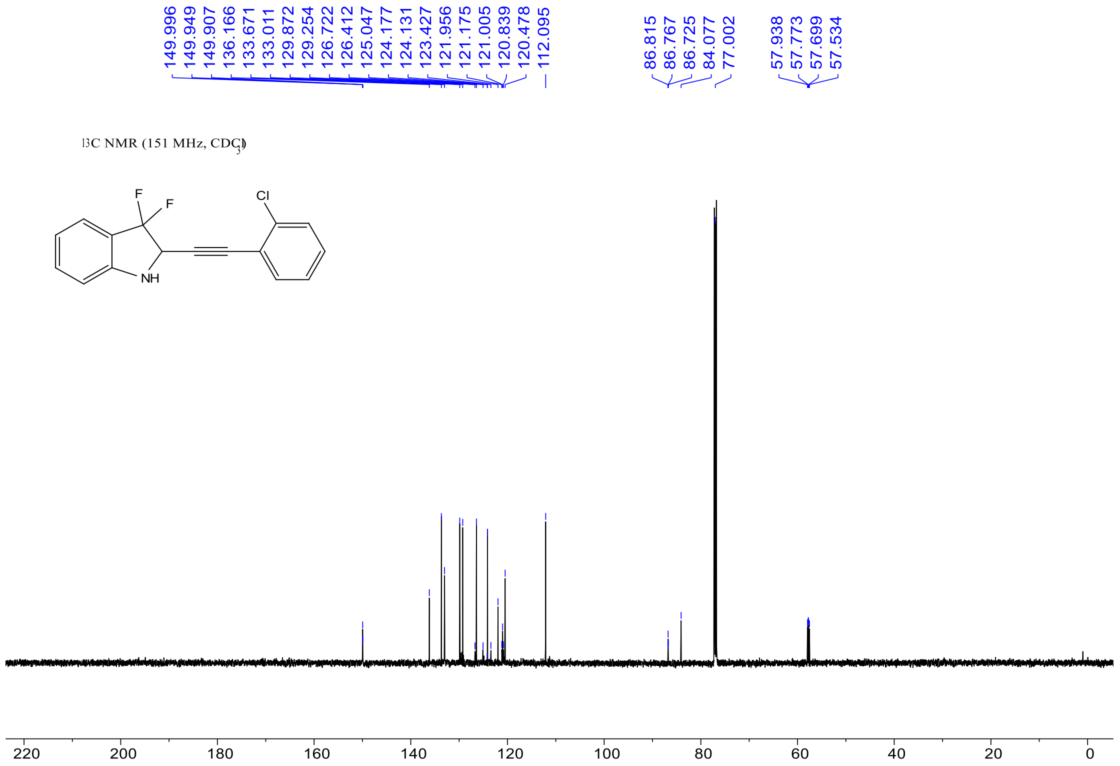

^{19}F NMR spectra of **2n**

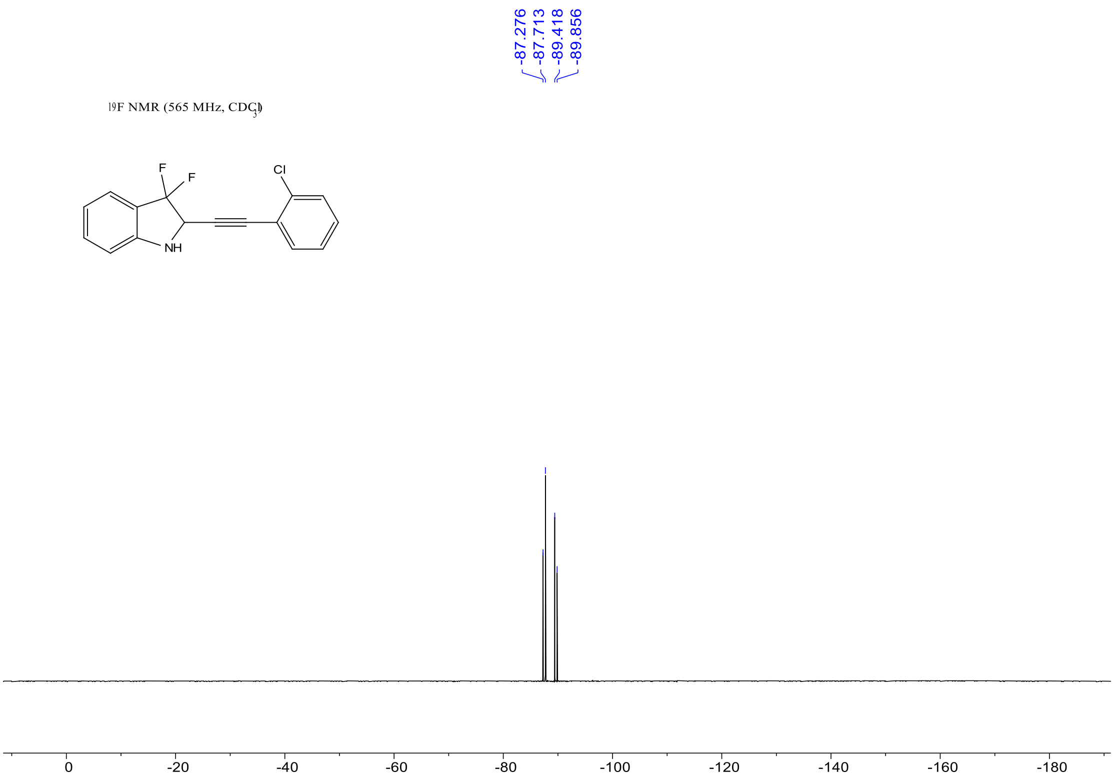

^{1}H NMR spectra of **2o**

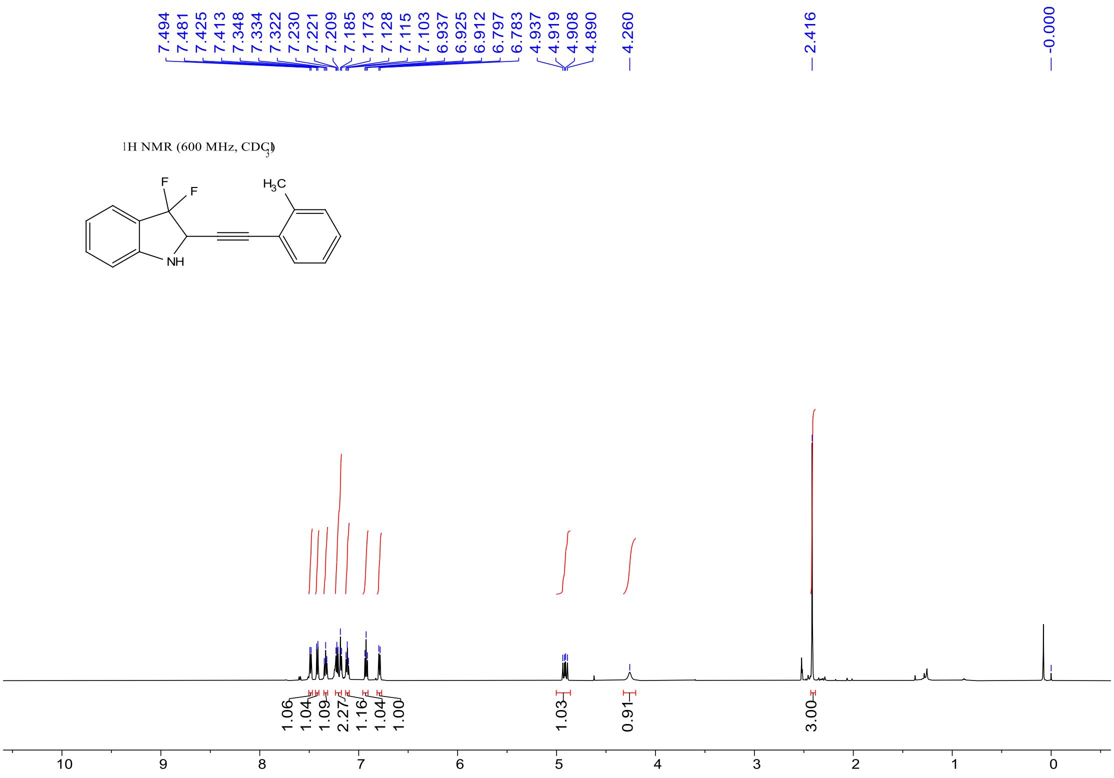

^{13}C NMR spectra of **2o**

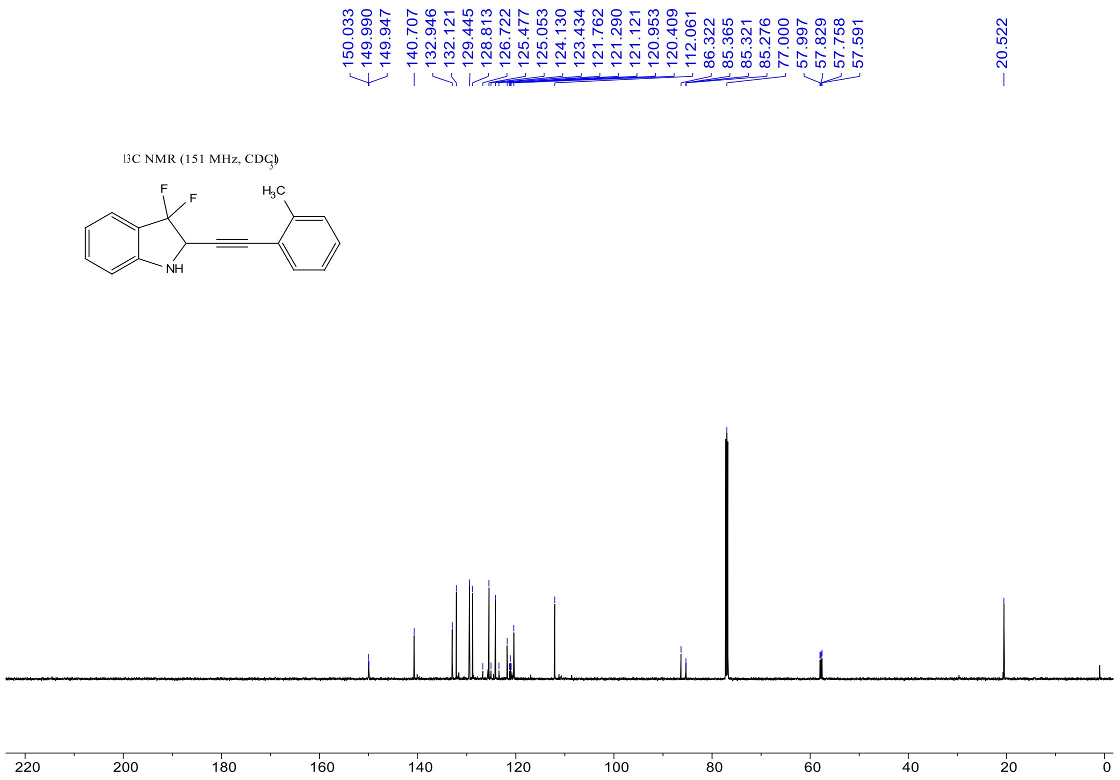

^{19}F NMR spectra of **2o**

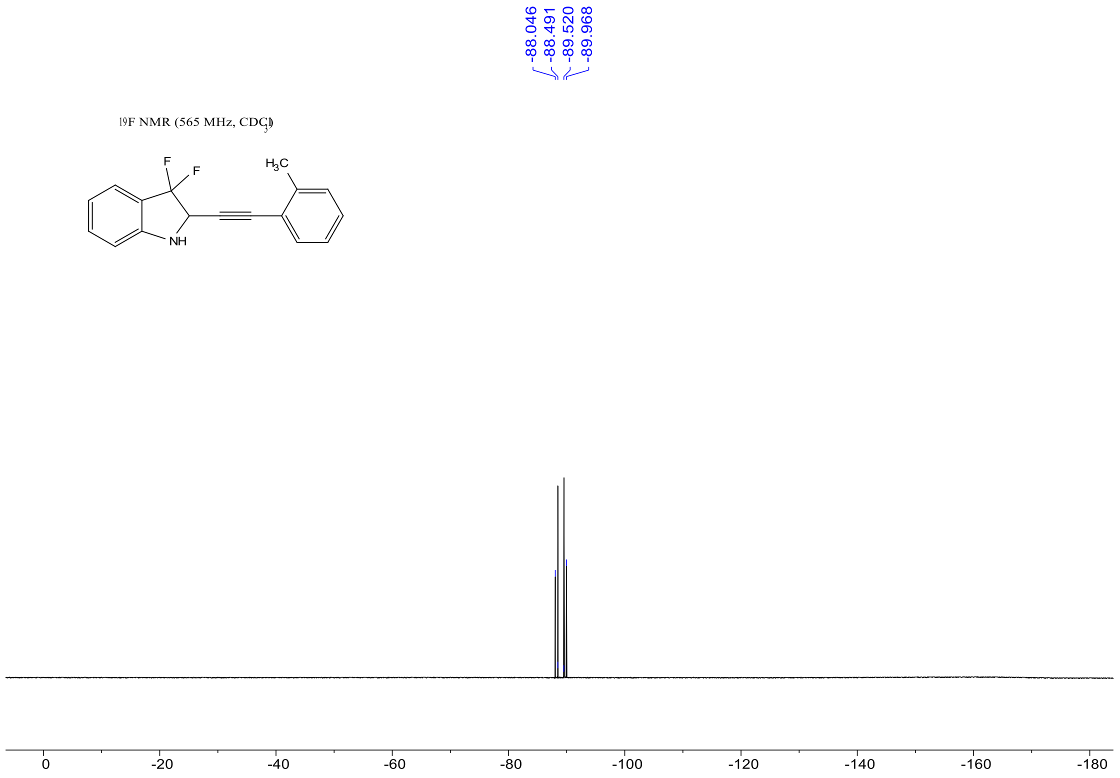

^{1}H NMR spectra of **2p**

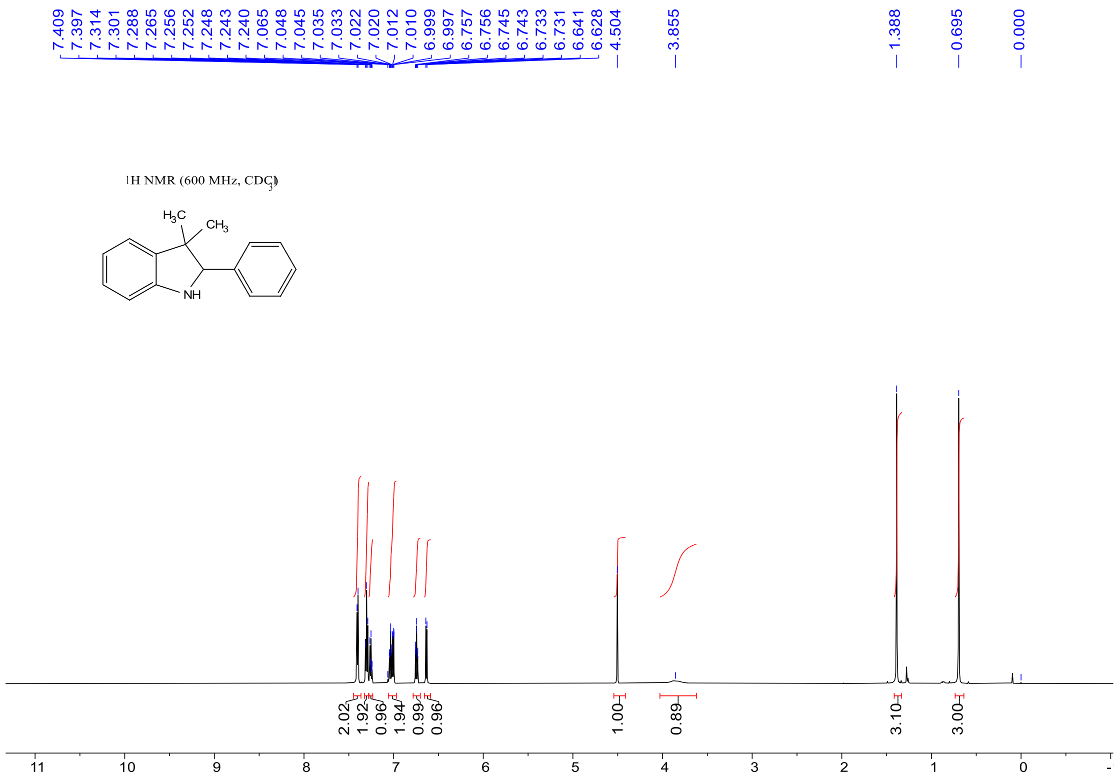

^{13}C NMR spectra of **2p**

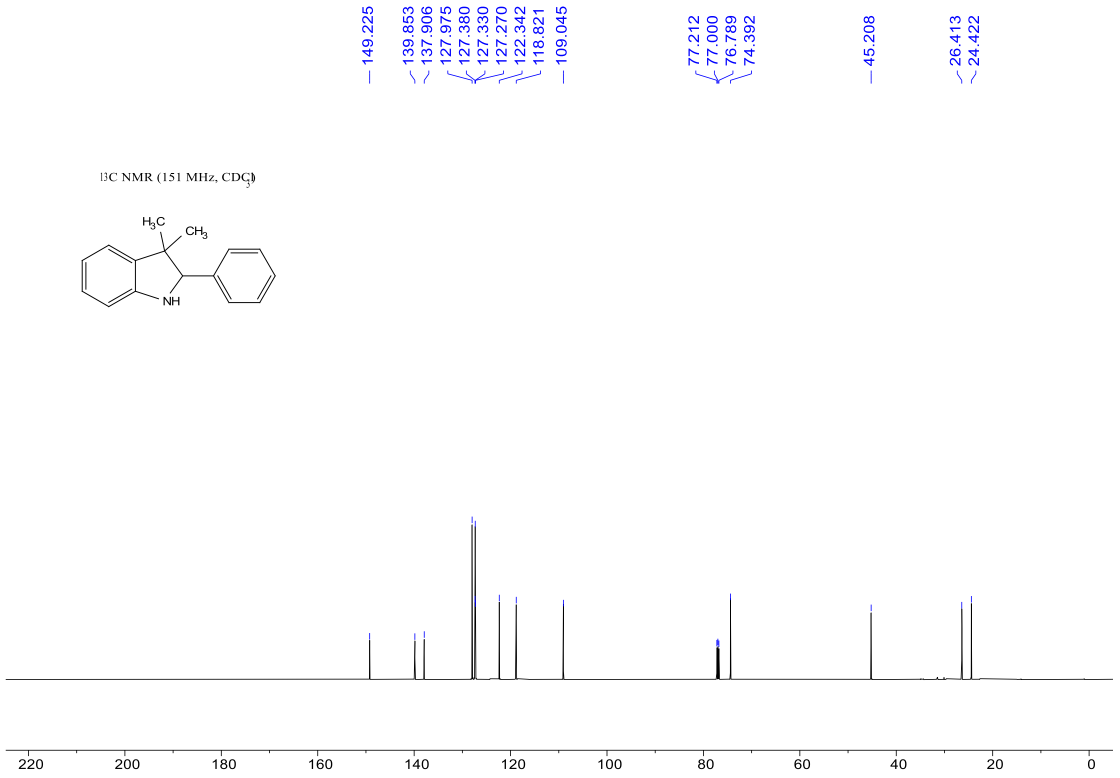

^{1}H NMR spectra of **2q**

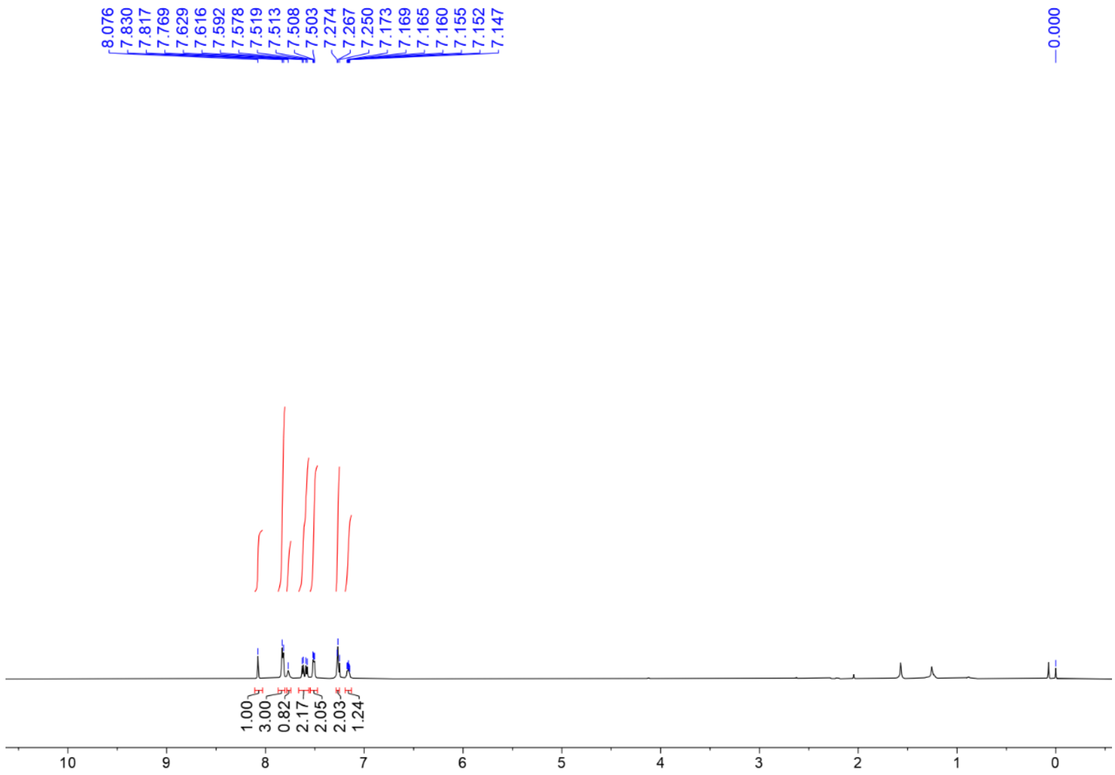

^{13}C NMR spectra of **2q**

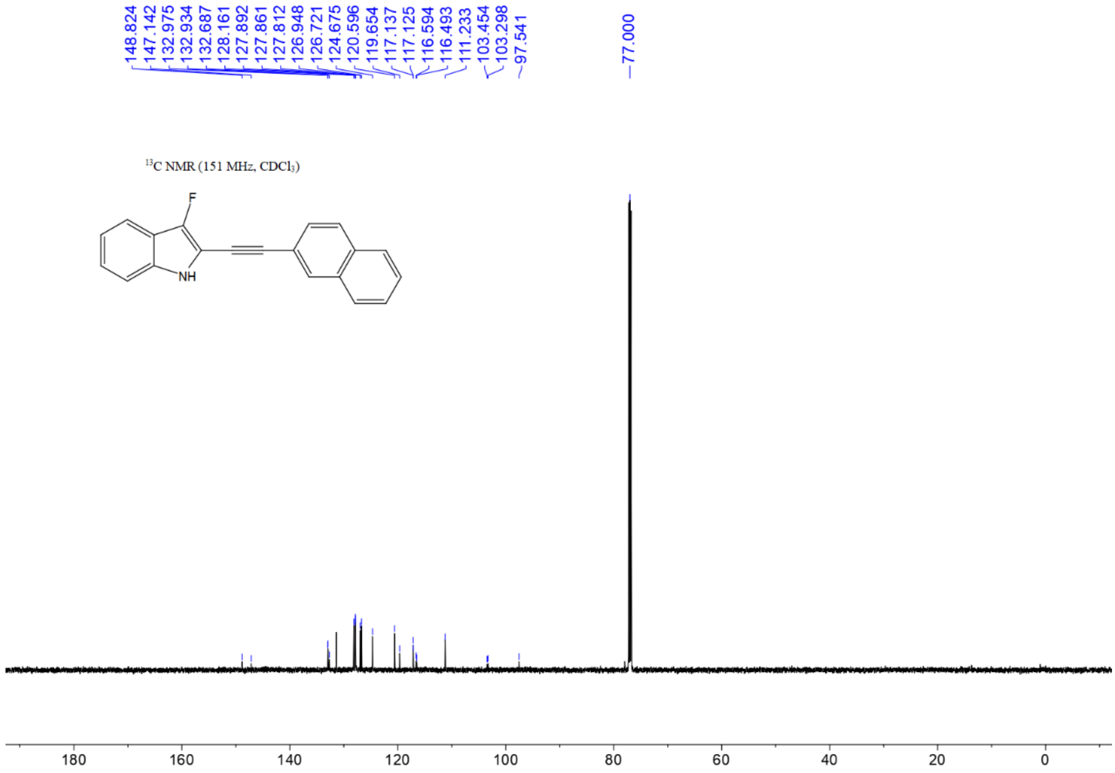

^{19}F NMR spectra of **2q**

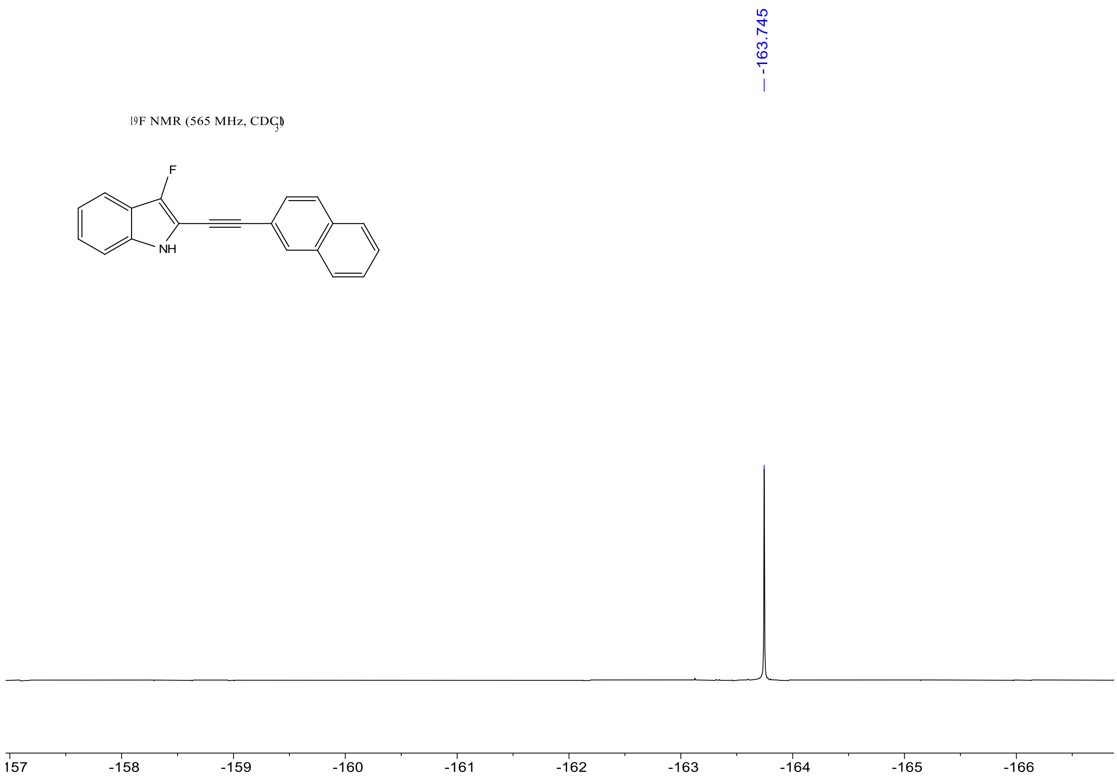

^{1}H NMR spectra of **2r**

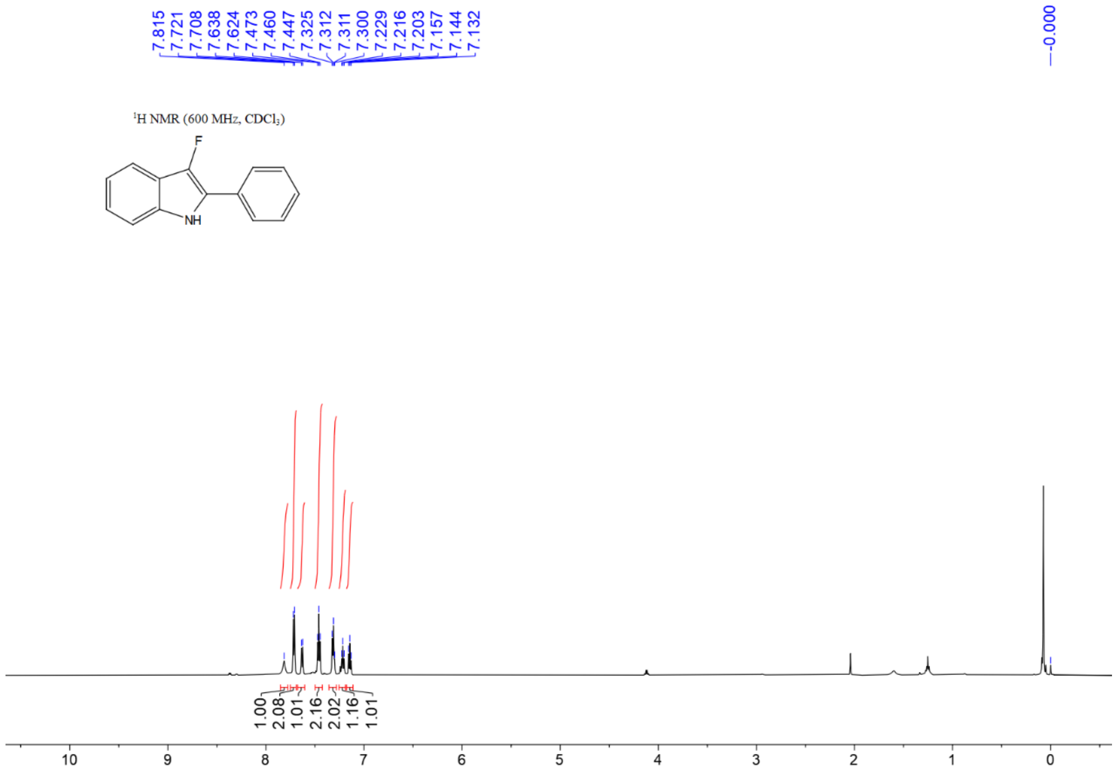

^{13}C NMR spectra of **2r**

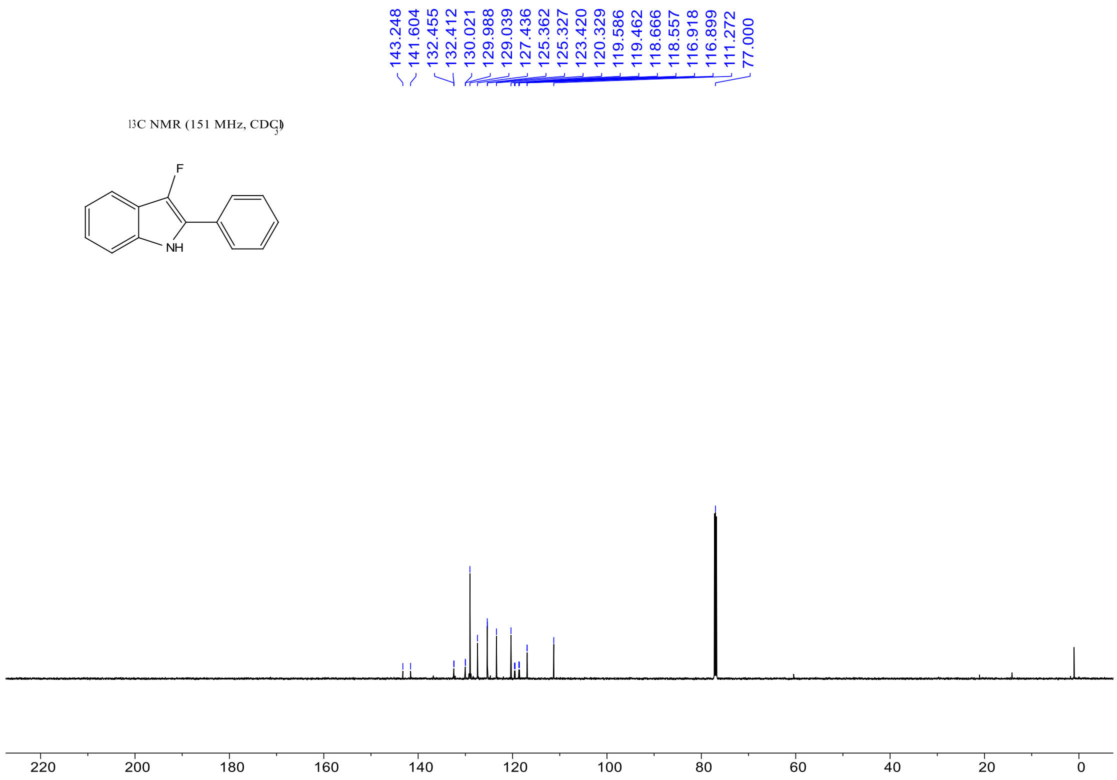

^{19}F NMR spectra of **2r**

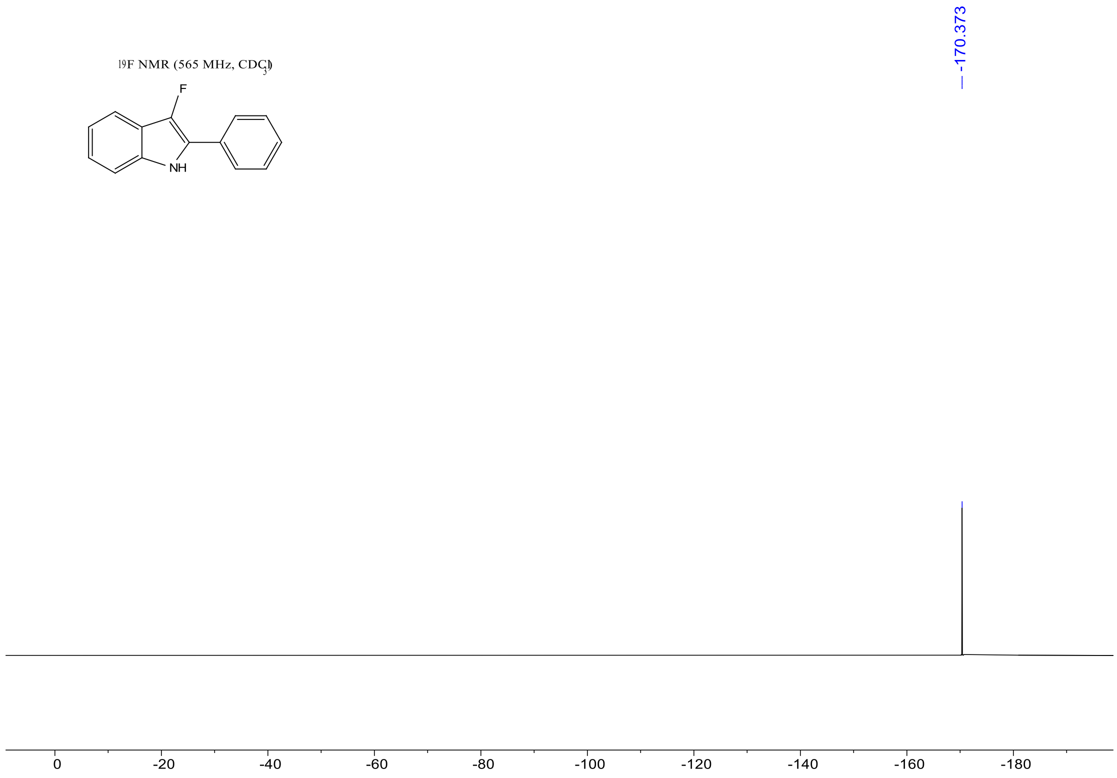

Supplement: File 1 — Full experimental details and characterization data of all compounds. [file Beilstein_J_Org_Chem-20-205-s001.pdf]
